# Neural responses to conflicting self- and partner-directed feedback correlate with trait emotion regulation and daily support

**DOI:** 10.1038/s44271-026-00493-w

**Published:** 2026-07-02

**Authors:** Shanshan Ma, Erin L. Maresh, Andrea M. Coppola, Katherine E. Richard, Leonie Koban, Diego Guevara Beltran, David A. Sbarra, Jessica R. Andrews-Hanna

**Affiliations:** 1 Department of Psychology, University of Arizona, Tucson, AZ USA; 2 Centre de Recherche en Neurosciences de Lyon (CRNL), CNRS, INSERM, Université Claude Bernard Lyon 1, Bron, France; 3 Le Vinatier - Psychiatrie Universitaire Lyon Métropole, Bron, France; 4 Cognitive Science, University of Arizona, Tucson, AZ USA; 5 Present Address: Center for the Study of Traumatic Stress, Department of Psychiatry, Uniformed Services University of the Health Sciences, Bethesda, MD USA; 6 Present Address: Department of Psychiatry, University of California San Diego, La Jolla, CA USA; 7 Present Address: VA San Diego Healthcare System, San Diego, CA USA; 8 Present Address: Department of Veterans Affairs, Desert-Pacific Mental Illness Research Education and Clinical Center, San Diego, CA USA

**Keywords:** Empathy, Human behaviour, Social behaviour

## Abstract

Empathy lies at the core of social interactions, particularly in intimate relationships, yet few laboratory tasks are designed to capture empathy in interpersonal contexts—especially those requiring individuals to simultaneously process social information directed toward both the self and a romantic partner. In this exploratory study, 131 adults (from 71 romantic couples) completed a neuroimaging paradigm that manipulated whether positive or negative partner-directed feedback was presented alone or alongside self-directed feedback. The partner-alone feedback contrasts (partner-directed feedback alone versus a neutral control condition) robustly engaged brain regions associated with empathic responding, including the dorsal anterior cingulate cortex, anterior insula, dorsomedial prefrontal cortex and temporoparietal junction. Notably, during the incongruent feedback contrasts—simultaneous conflicting self- and partner-directed feedback versus self-directed feedback alone—participants exhibited diminished affective and brain responses but evidenced unique brain activity in frontoparietal areas frequently associated with cognitive control (e.g., dorsolateral prefrontal cortex and supramarginal gyrus). Furthermore, when responding to positive partner-directed feedback in the presence of negative self-directed feedback, enhanced activity in the dorsomedial prefrontal cortex was associated with lower self-reported difficulties in emotion regulation and more partner-rated support provided in everyday life. Taken together, these findings suggest that salient self-focused information may dampen individuals’ emotional and neural responses to their partners’ uplifting and critical feedback, that emotion regulation tendencies may be an important between-subjects moderator of this effect, and that individual differences in spontaneous neural responses during this task were associated with meaningful real-world outcomes, although further validation and replication are warranted.

## Abstract (web summary)

This study suggests that self-focused information may dampen emotional and neural responses to partner feedback, that emotion regulation may moderate this effect, and that neural responses during the task relate to daily supportive behavior.

## Introduction

Empathy exerts a profound influence on prosocial behavior^1–3^, social connectedness^4^, and mental health^5^, particularly in the context of close relationships^6,7^. While definitions of empathy vary in the literature, recent interdisciplinary efforts describe empathy as the ability to experience others’ affective states while maintaining a distinction from one’s own emotional experiences^8^. Despite differences in terminology, there is broad agreement that empathy implies three processes: experience sharing, mentalizing, and prosocial concern^9,10^. Although many laboratory-based tasks succeed at eliciting empathy in controlled experimental settings^2,11–13^, capturing the spontaneous empathic responses that occur in everyday life has proven more challenging^14,15^. In daily life, acute stress and salient emotional states can make spontaneous empathy toward others difficult, if not impossible, in some circumstances^16–18^. In these situations, regulating self-focused thoughts and feelings to understand and share the experience of another may require notable cognitive effort. One understudied context in which these kinds of challenging situations often occur is in the context of close relationships. For example, imagine you receive negative feedback from your supervisor, but your partner tells you they received a wonderful promotion at work. How do you respond? In this manuscript, we use functional MRI to examine how participants in romantic relationships respond to their partners’ experiences in the presence of incongruent self-related social feedback (e.g., when they are rejected but their partner is liked). We hope to bridge the gap between controlled laboratory settings and the complexities of everyday social interactions that can interfere with empathic behavior.

A key starting point for our exploration is the challenge associated with managing self-relevant information and, at times, regulating self-relevant thoughts and feelings. Prioritizing self-relevant information is believed to be evolutionarily advantageous, and behavioral studies demonstrate that self-referential stimuli produce a stronger interference on the processing of other-related stimuli than the reverse situation^19,20^. Failing to regulate one’s emotions or inhibit self-relevant responses can lead to *egocentricity bias*, defined as the interference caused when one’s own thoughts or emotions influence judgments about another person, which may ultimately reduce empathic responses toward others^21,22^. For example, acute stress and negative mood can have detrimental consequences on one’s affective sharing and mentalizing^16,18,19^. Despite the central role of self- and emotion-regulatory processes in many models of empathy^23–26^, relatively little is known about how the brain processes other-relevant information in the presence of simultaneous self-relevant information, how these mechanisms vary across individuals in emotion regulation tendencies, and whether they are relevant for explaining real-world prosocial behavior.

Although a large body of research has established the role of several interacting brain regions in supporting the capacity to share others’ emotions and understand others’ mental states–including the anterior insula (AI), dorsal anterior cingulate cortex (dACC), dorsomedial prefrontal cortex (DMPFC), temporoparietal junction (TPJ), posterior cingulate cortex (PCC), and ventromedial prefrontal cortex^10–12^—relatively few studies have directly manipulated emotion- and self-regulatory demands during empathic processing. Many laboratory paradigms involve simply observing or processing stimuli being encountered by others^1,2,12^, which cannot fully capture the complexity of empathy in the real world where multiple sources of emotionally salient, self- and other-relevant information must be processed simultaneously. Silani et al. investigated the neural underpinnings of overcoming the egocentricity bias in the emotional domain, primarily under highly controlled and instructed judgment conditions^21^. The authors introduced a visuo-tactile paradigm, where participants were explicitly instructed to make judgments about either their own or a stranger’s affective state upon presumably interacting with the tactile stimuli, while simultaneously viewing self- and other-related stimuli that could be affectively congruent or incongruent. Their findings highlighted the role of the right supramarginal gyrus (SMG) in overcoming the emotional egocentricity bias^21^. Despite the contribution of this work, there remains a need for paradigms that capture spontaneous empathic responses, without explicitly instructing participants to empathize, especially in challenging interpersonal situations.

Empathy is often elicited in the context of close (especially romantic) relationships^15^ and is considered vital to relationship quality and mental well-being^6,7,27^. However, most of the existing social neuroscientific research focuses on understanding and empathizing with strangers^1–3,11^, which may diverge to some degree from the neural mechanisms in the context of relationships^28,29^. Although some studies have examined empathy within romantic relationships, they primarily focus on empathy for pain^30,31^, which overlaps with but may be different from empathic responses evoked by social negative states (e.g., anger or distress)^32^. Studying empathy in social contexts can therefore provide additional insights into how empathic responses unfold in everyday interpersonal situations. Furthermore, empathy for positive emotions, which also plays a functional role in prosocial behavior, social closeness, and well-being^33,34^, has received limited attention. Addressing these gaps will enhance our understanding of empathy within close relationships in everyday life.

**Fig. 1:**
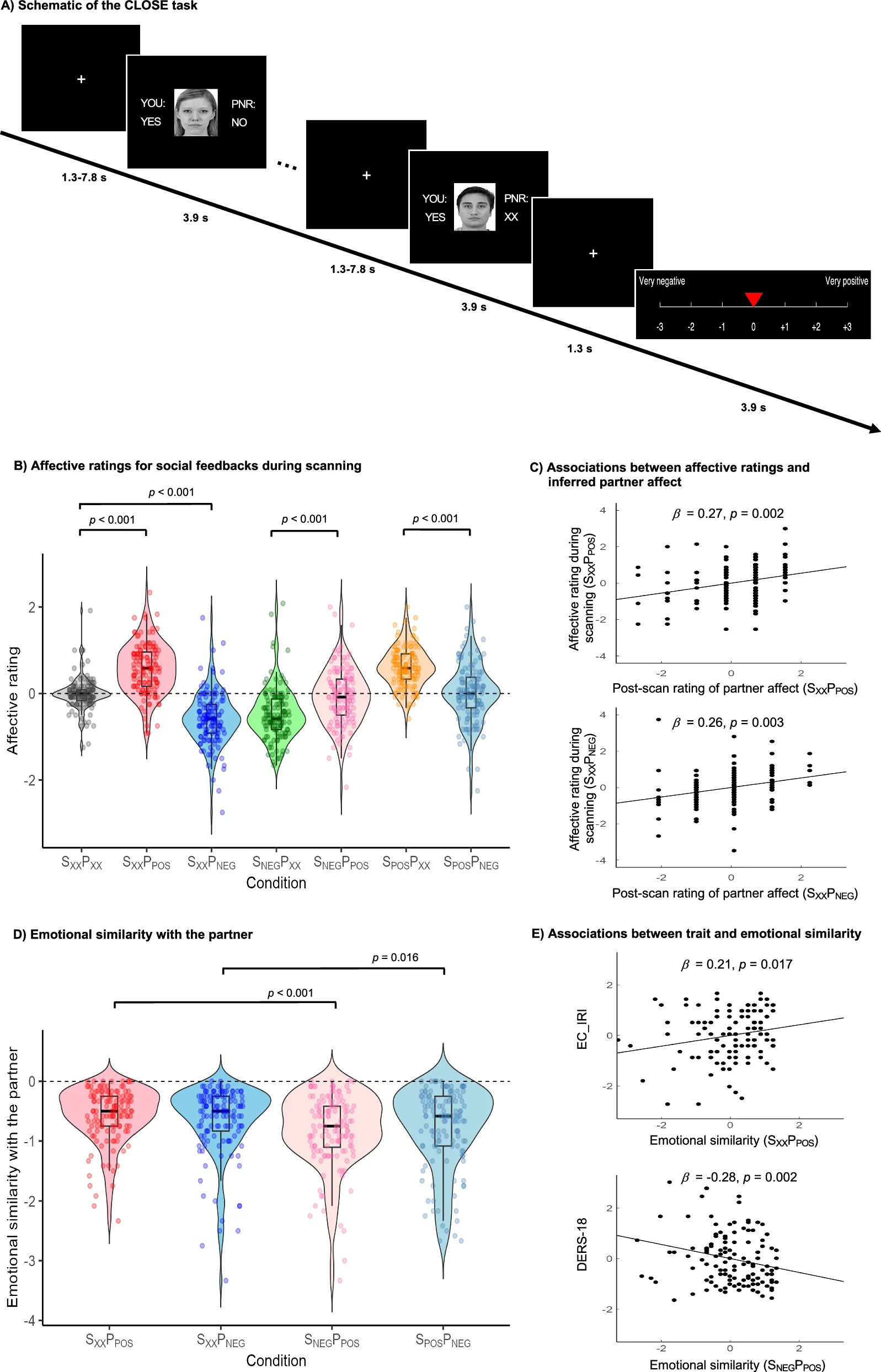
Schematic of the CLOSE task and affective ratings during the task. **A** The CLOSE task presents a rater face along with positive (YES), negative (NO), or no feedback (XX) directed toward both oneself (YOU) and one’s partner (PNR). Following half of the trials within each condition, participants were asked to rate their affective state from -3 (very negative) to 3 (very positive). The rater face images were obtained from the Chicago Face Database with permission of publication granted by the database administrators. **B** Participants reported higher positive or negative affective ratings when they received positive or negative partner-directed feedback alone, compared to when they received no feedback about their partner or themselves. Participants reported higher positive or negative affect in response to positive or negative partner-directed feedback, respectively, when simultaneously receiving incongruent self-directed feedback, compared with receiving self-directed feedback alone (*N* = 131). S = self-directed feedback, P = partner-directed feedback, POS = positive feedback, NEG = negative feedback, XX = no feedback. **C** Significant positive associations between participants’ affective ratings during scanning and their post-scan inferred partner affect in both positive and negative partner-directed feedback alone conditions (*N* = 129). The values of the horizontal and vertical coordinates have been standardized. **D** Emotional similarity with the partner was significantly lower in the S_NEG_P_POS_ or S_POS_P_NEG_ condition than S_XX_P_POS_ or S_XX_P_NEG_ condition (*N* = 128). Emotional similarity with the partner under each feedback condition was computed by comparing a participant’s self-reported affective rating with their partner’s self-reported affect in response to the matched feedback condition during scanning. Note that the *p* value displayed in the plot was derived via bootstrapping (5000 iterations) due to deviation from model residual assumptions. **E** Higher empathic concern (EC_IRI) was positively associated with emotional similarity with the partner in the S_XX_P_POS_ condition, while greater difficulties in emotion regulation (DERS-18) were negatively associated with emotional similarity with the partner in the S_NEG_P_POS_ condition (*N* = 128). The values of the horizontal and vertical coordinates have been standardized. The broader shape of the violin plots illustrates the data distribution, with wider sections indicating higher density. The nested box plots display the median line (center line), quartiles (box edges representing the 25th and 75th percentiles), and whiskers extending to 1.5 × the interquartile range (IQR).

To better approximate empathic contexts in everyday life and to investigate individuals’ spontaneous responses to their romantic partners’ social experiences during challenging interpersonal situations, the current study employed a dyadic functional magnetic resonance imaging paradigm—the Connected Lives: Overcoming the Self through Empathy (CLOSE) task^35^. The CLOSE task is adapted from social feedback paradigms, where participants receive positive, neutral, or negative feedback about their desirability or likability^36,37^, and empathy paradigms which involve observing stimuli being encountered by others^2,12^. The task displays a photograph of a “rater” along with the rater’s presumed likability ratings of the participant in the scanner (“YOU”) and/or the participant’s partner (“PNR”), who the participant believes will also view the same stimuli in another room, based on a cover story to facilitate spontaneous empathy. The rater’s likeability ratings vary in valence, consisting of positive (POS, indicated by “YES”), negative (NEG, indicated by “NO”), and absent (no ratings, indicated by “XX”) ratings (Fig. 1A). To create a more naturalistic context capturing spontaneous empathic responses while minimizing demand characteristics inherent to many existing paradigms, the task avoids explicit instructions to focus on oneself or to empathize with one’s partner. Rather, participants’ affective responses were assessed following half of the trials in each condition. When combined with post-task retrospective questionnaires evaluating the extent to which participants’ affective responses tracked how they believed their partner would feel, as well as dyadic analyses examining how participants’ responses aligned with their partners’ actual feelings, these measures provide behavioral validation that the paradigm evokes spontaneous empathy.

Although the CLOSE task yields several task conditions for analysis in other ongoing work, we isolated brain and behavioral contrasts linked to low versus high self-regulatory demands. Specifically, to measure spontaneous responses to partner-directed feedback under low demand for regulating responses to self-directed feedback, we compared *partner-only* conditions—where the self (S) received no feedback and the partner (P) received positive or negative feedback (S_XX_P_POS_ or S_XX_P_NEG_)—to a *neutral control condition* matched on visual properties but lacking self-directed or partner-directed feedback (S_XX_P_XX_) (see Methods for cover story behind feedback-absent conditions). These *partner-alone feedback contrasts* (S_XX_P_POS_-S_XX_P_XX_ and S_XX_P_NEG_-S_XX_P_XX_) are similar to conventional empathy contrasts used in previous research. Additionally, to evaluate spontaneous responses to partner-directed feedback under higher demand for regulating responses to self-directed feedback, we contrasted *simultaneous incongruent* conditions—where opposing positive and negative feedback was directed toward the self and partner (S_POS_P_NEG_ or S_NEG_P_POS_)—against *self-only* conditions involving positive or negative self-directed feedback (S_POS_P_XX_ or S_NEG_P_XX_). We refer to these contrasts (S_POS_P_NEG_-S_POS_P_XX_ and S_NEG_P_POS_-S_NEG_P_XX_) as *incongruent feedback contrasts* in our study.

In this report, we first examined whether the partner-alone feedback contrasts (S_XX_P_POS_-S_XX_P_XX_ and S_XX_P_NEG_-S_XX_P_XX_) elicited behavioral (self-reported affect) and BOLD activity consistent with prior empathy research. We hypothesized that we would observe activity in regions previously observed in empathy studies, such as the AI, dACC, DMPFC, TPJ, PCC, and ventromedial prefrontal cortex^10–12^. We then expanded our analysis to the incongruent feedback contrasts (S_POS_P_NEG_-S_POS_P_XX_ and S_NEG_P_POS_-S_NEG_P_XX_) to investigate participants’ spontaneous responses to partner-directed feedback in the presence of incongruent self-directed feedback. Since the task places high demands on regulating responses to self-directed feedback, we hypothesized that participants would also recruit regions associated with emotion regulation, such as the dorsolateral prefrontal cortex (DLPFC)^38,39^, and regions previously linked to overcoming the emotional egocentricity bias (such as the right SMG)^21^, beyond regions associated with empathy. Of particular importance, we aimed to test the hypothesis that between-person variability in BOLD activity during the incongruent feedback contrasts would be related to individual differences in emotion regulation tendencies and trait empathy, as well as with empathic behavior in everyday life, which we assessed via support provided to one’s partner using a week-long daily diary, completed by the partner to boost the validity of the findings. Our work was not preregistered, and we thus consider it exploratory and discovery-oriented despite our *a priori* hypotheses.

## Methods

### Participants

Seventy-three couples (*N* = 146 participants) in monogamous romantic relationship were recruited from the Tucson community using flyers and advertisements on social media. Couples were required to have been together for at least 18 months and to have lived together for at least 6 months. This study is part of a larger ongoing study involving the collection of neural, physiological, and behavioral data among romantic couples. It was approved by the Institutional Review Board of the University of Arizona and all participants provided written informed consent prior to participation. Fifteen participants were excluded from the subsequent analysis due to either the absence of task or structural neuroimaging scans (*n* = 10), or compromised neuroimaging data resulting from severe coverage loss or asynchrony between the experimental procedure and brain scanning (*n* = 5). Thus, the final neuroimaging sample contained 131 subjects from 71 couples. Among these participants, 76 individuals were married. The mean age of the final sample was 31.8 years (*SD* = 8.4, range = 19.1–56.1), the mean relationship duration was 8.3 years (*SD* = 6.3, range = 1.7–30.0), and the mean cohabiting duration was 6.5 years (*SD* = 6.2, range = 0.5–29.5). Detailed demographic characteristics are displayed in Supplementary Table 1.

### Experimental procedure

The experimental procedure included two laboratory sessions, a daily diary completed by participants’ partners each night between the two sessions, and a 6-month follow-up survey (not included in the current study). Participants were compensated $15 for completing the online interview, $35 for Session 1, $40 for completing the experience sampling between sessions, $70 for Session 2, and $40 for completing the 6-month follow-up survey, for a total of $200 per person ($400 per couple).

In Session 1, participants completed demographic questionnaires, three social interaction tasks not analyzed in this manuscript, and a face rating task designed to build a cover story to increase believability of the social feedback ratings in the CLOSE task. In the face rating task, participants rated the likability of 30 photos thought to be other participants who completed Session 1 (these photos differed from those shown in Session 2). Participants were told: “We have a separate ongoing study in which hundreds of participants have already had their pictures taken. The selection of people that you just rated were drawn from this other study”. Participants pressed the right arrow if the person appeared likable to them (displaying the word “YES” on the screen) and the left arrow if the person did not appear likable (displaying the word “NO”). If participants did not press an arrow key within 2 s, the text “XX” was displayed and the task advanced to the next trial. Additionally, an experimenter also took the participant’s own photo in Session 1 to convince them that their photos would be distributed to other participants to rate their likability.

In Session 2, both partners completed MRI scanning, including a resting state scan, a structural T1 scan, the CLOSE task, and a field map. While one participant was being scanned, their partner completed a battery of questionnaires in a different room assessing several constructs (see the measurement list available on https://osf.io/63vnc/files/c4t7r). These measurements included trait empathy (e.g., the Empathy Assessment Index and the Interpersonal Reactivity Index (IRI)), trait emotion regulation (e.g., the Difficulties in Emotion Regulation Scale (DERS-18)), relationship quality (e.g., the Revised Dyadic Adjustment Scale and the Couples Satisfaction Index), psychological well-being and symptoms of mental health disorders (e.g., the Patient-Reported Outcomes Measurement Information System—Depression and Anxiety, and the UCLA Loneliness Scale), and trauma exposure (e.g., the Adverse Childhood Experiences scale). While several of these metrics will be the focus of future analyses, the IRI and DERS-18 were selected for the purposes of current study as commonly used metrics of trait empathy and emotion regulation tendencies (see below).

The CLOSE task—a social feedback task–was used to assess spontaneous empathy as demands to regulate the self-focused thoughts and feelings vary across conditions. Participants were informed that the task involves viewing likability ratings made by other participants in the study about themselves and their partner. Critically, participants were told that “You and your partner will each be viewing feedback from the same participants during your scans”, setting up a context to facilitate a sense of their partner’s presence and promote spontaneous empathy. Prior studies using the CLOSE paradigm on different subsamples have provided initial support for its validity, but they did not examine neural responses under incongruent feedback conditions. Specifically, previous work has demonstrated behavioral associations between relational interdependence and the subjective valuation of partner-related feedback^35^, as well as task-related engagement of self- and other-mentalizing neural signatures during feedback processing compared to a no-feedback control condition^40^.

### The CLOSE task

#### Experimental conditions and trial structure

In each trial of the CLOSE task, a different photograph of a “rater” was displayed for 3.9 s, along with the rater’s presumed likability ratings of the participant in the scanner (“YOU”) and the participant’s partner (“PNR”). The rater’s likeability ratings varied in valence, consisting of positive (POS, rating = “YES”), negative (NEG, rating = “NO”), and absent (rating = “XX”) ratings. For absent ratings, which served as a control condition for the CLOSE task, participants were told that the rater did not view their photo or failed to make a response to their photo fast enough (Fig. 1A). The CLOSE task included the following task conditions: a) Self-directed feedback but no partner-directed feedback (S_NEG_P_XX_ and S_POS_P_XX_), b) Partner-directed feedback but no self-directed feedback (S_XX_P_NEG_ and S_XX_P_POS_), c) Simultaneous congruent feedback (S_NEG_P_NEG_ and S_POS_P_POS_), d) Simultaneous incongruent feedback (S_NEG_P_POS_ and S_POS_P_NEG_), and e) No feedback for either self or partner (S_XX_P_XX_). Although we told participants they would receive likability ratings made by other participants, all of the “raters” were photos taken from the Chicago face database^41^. All photographs were black and white headshots of individuals making a neutral expression, and conditions were matched on perceived attractiveness, age, and race/ethnicity of the photos (using normed ratings of the Chicago face database).

#### Task timing and affective ratings

To create a more naturalistic context that minimizes demand characteristics and captures participants’ propensity to evoke empathy in a spontaneous fashion, we avoided explicitly instructing individuals to empathize with their partner. Following half of the trials within each condition, participants were asked to rate their current affective state by referring to the question “Right now, how do you feel, with -3 being very negative and 3 being very positive?”. There was a 1.3-second interval following the presentation of stimuli presenting likability ratings, and then participants were then given 3.9 s to supply their response with a button press placed in their dominant hand.

Participants completed 6 runs of the CLOSE task, and each run consisted of 36 trials with 4 trials per condition. The trial order was counterbalanced with a 1.3–7.8 s jittered fixation inter-trial interval, which was determined using the fMRI trial timing optimization program optseq2^42^. In total, the duration of each run was approximately 5 min and 35 s. Two runs from one participant and one run from another were removed due to scanning issues.

#### Post-scan ratings and funnel debriefing

After the scan, participants completed separate retrospective questionnaires in which they rated their emotions and speculated about their partner’s emotions during each condition in the CLOSE task (from -3 (very negative) to 3 (very positive)). Two participants didn’t complete this questionnaire (*N* = 129).

Because of the deception in this study, participants also completed a funnel debriefing procedure after scanning in Session 2. They were asked to rate the following questions on a scale from 1 to 5: (1) “During the task, to what degree did you feel like the feedback you and your partner received about your likability was based on the photos taken of you and your partner in the first study session?” and (2) “During the task, to what degree did you feel like the feedback you and your partner received about your likability was made by other study participants?” Two participants did not report on these two questions (*N* = 129). The mean values of these two belief ratings were 3.6 (*SD* = 1.2) and 3.6 (*SD* = 1.3), respectively.

### Trait and daily diary measures

#### Interpersonal reactivity index and difficulties in emotion regulation scale

On the scanning day, participants completed selected subscales from the IRI and DERS-18 to assess participants’ self-reported trait empathy and emotion regulation tendencies. Specifically, we administered the 21-item IRI, which includes three of the four standard subscales: (a) Empathic Concern, (b) Perspective Taking, and (c) Personal Distress. Items are rated on a 5-point Likert scale from “Does not describe me well” to “Describes me very well.” In the current study, we focused on two key subscales most relevant to empathic responding: Empathic Concern (e.g., “I am often quite touched by things that I see happen”) and Perspective Taking (e.g., “I try to look at everybody’s side of a disagreement before I make a decision”)^43^.

The DERS-18 is a validated brief version of the original 36-item Difficulties in Emotion Regulation Scale^44^. It includes 18 items spanning six subscales: (1) Awareness, (2) Clarity, (3) Goals, (4) Impulse, (5) Nonacceptance, and (6) Strategies. Each item is rated on a 5-point Likert scale ranging from “Almost Never” to “Almost Always” (e.g., “When I’m upset, I have difficulty getting work done”). Sum scores range from 18 to 90 with higher total scores indicate greater difficulties in emotion regulation^44^.

#### Partner-rated daily support provided in a daily diary survey

Participants completed an end-of-day online survey for the consecutive days spanning from Session 1 to Session 2, including questions assessing aspects of empathy and relationship characteristics. For the present study, to index daily support processes within couples, we assessed support provided to the partner using partner reports, an approach that helps increase accuracy and reduce self-reported biases^45,46^. Specifically, participants’ daily support provided to their partner was indexed by their partner’s responses to the item: “How much support did you receive from your partner today?” (0 = none, 4 = a great deal). Responses were averaged across daily surveys to obtain each participant’s mean daily supportive behaviors as reported by their partner. We excluded surveys that were not completed within 24 h of participants receiving the survey email to enhance the reliability of the daily diary data. In cases where ratings for certain items were missing, the mean scores were computed using only the available, non-missing data. On average, the time interval for this survey was 7.8 days *(SD* = 1.4), and participants submitted a mean of 7.2 diary entries during this period (*SD* = 1.5).

### MRI data acquisition

Functional MR imaging was conducted with a 3-Tesla Siemens MRI scanner. Forty-two interleaved axial slices were acquired using a gradient-echo echo-planar imaging sequence with repetition time = 1300 ms, echo time = 30 ms, flip angle = 68°, field of view = 252 mm × 252 mm, voxel size = 3 × 3 × 3 mm^3^, matrix size = 84 × 84, and acceleration factor = 2. One hundred seventy-six high-resolution slices were obtained using a T1-weighted sagittal 3-D magnetization prepared rapid gradient echo sequence: repetition time = 2300 ms, echo time = 2.95 ms, inversion time = 900 ms, flip angle = 9°, field of view = 270 × 270 mm, voxel size = 1.1 × 1.1 × 1 mm^3^, and matrix size = 256 × 256. Forty-two axial field map slices were acquired using a gradient-echo sequence: repetition time = 467 ms, echo time1 = 4.92 ms, echo time2 = 7.38 ms, flip angle = 60°, field of view = 224 × 224 mm, voxel size = 3 × 3 × 3 mm^3^, and matrix size = 74 × 74.

### Data analysis

#### Behavioral analyses

At first, we examined participants’ affective ratings during scanning. Specifically, we tested the affect changes associated with the partner-alone feedback contrasts involving viewing partner-directed feedback alone (i.e., S_XX_P_NEG_-S_XX_P_XX_ and S_XX_P_POS_-S_XX_P_XX_), as well as the affective responses to incongruent feedback contrasts involving social feedback directed at one’s partner when receiving simultaneously incongruent social feedback about the self (i.e., S_NEG_P_POS_-S_NEG_P_XX_ and S_POS_P_NEG_-S_POS_P_XX_). Next, we conducted additional analyses to investigate the interference effect of incongruent self-directed feedback on spontaneous responses to partner-directed feedback. Specifically, we compared affective ratings between the S_NEG_P_POS_-S_NEG_P_XX_ and S_XX_P_POS_-S_XX_P_XX_ contrasts, as well as between S_POS_P_NEG_-S_POS_P_XX_ and S_XX_P_NEG_-S_XX_P_XX_ contrasts.

Additionally, to assess affective experience sharing, we computed emotional similarity with one’s partner under each feedback condition by comparing a participant’s self-reported affective rating with their partner’s self-reported affect under the corresponding feedback condition during scanning. Specifically, emotional similarity with one’s partner in the S_XX_P_POS_ condition was computed as the negative value of absolute differences between a participant’s affective rating in the S_XX_P_POS_ condition with their partner’s self-reported affective rating in the S_POS_P_XX_ condition. A similar approach was taken with the S_XX_P_NEG_ condition. Emotional similarity with one’s partner in the S_NEG_P_POS_ condition was computed as negative value of absolute differences between a participant’s affective rating in the S_NEG_P_POS_ condition with their partner’s self-reported affective rating in the S_POS_P_XX_ condition. Finally, emotional similarity with one’s partner in the S_POS_P_NEG_ condition was computed as negative value of absolute differences between a participant’s affective rating in the S_POS_P_NEG_ condition with their partner’s self-reported affective rating in the S_NEG_P_XX_ condition. Next, we examined the associations between scores on the empathic concern subscale of the Interpersonal Reactivity Index (EC_IRI), reflecting the affective component of empathy, and emotional similarity with the partner in partner-alone and incongruent feedback conditions, as well as the associations between DERS-18 scores and emotional similarity with the partner in incongruent feedback conditions.

Furthermore, to test the cognitive related process during the task, we investigated whether participants’ affective responses during scanning were associated with how they believed their partner felt in response to the positive or negative feedback, which assessed using retrospective questionnaires after scanning (i.e., inferred partner affect in the S_XX_P_POS_ or S_XX_P_NEG_ condition). To account for the nested structure of contrasts within couples, linear mixed-effects models with a random intercept were employed to analyze all affective ratings described above, implemented using R software (R Core Team, version 4.4.0, https://www.r-project.org), with the lmerTest package.

#### Neuroimaging preprocessing

Functional imaging data were preprocessed using the robust open-source preprocessing pipeline, fMRIPrep (v23.0.1)^47^. Preprocessing steps included field map-based distortion correction (FUGUE), motion correction (MCFLIRT), slice timing correction (3dTShift), co-registration to T1-weighted structural images (bbregister), normalization to the Montreal Neurological Institute 152NLin6Asym template with a 2-mm isotropic resolution through nonlinear registration (antsRegistration), spatial smoothing using an isotropic Gaussian kernel with a full-width at half-maximum (FWHM) of 6 mm, and automatic removal of motion artifacts using independent component analysis (ICA-AROMA)^48^.

#### General linear model analyses of empathy-related neural responses

The cleaned data from fMRIPrep were passed to FSL’s fMRI Expert Analysis Tool (FEAT, version 6.00, FMRIB’s Software Library, www.fmrib.ox.ac.uk/fsl) for task-related general linear model analyses. The BOLD signal was modeled by convolving the onset and duration of 3.9-s feedback presentation phases for each of the 9 experimental conditions with a double-gamma hemodynamic response function. A separate regressor was also modeled to account for the affect ratings. Then, a second-level fixed-effects analysis was used to compute the average of the parameter estimates across the runs within each subject, and a third-level analysis with mixed effects (FLAME1) was used to test generalization to the population from which our sample was drawn.

We performed a series of whole-brain analyses to examine the BOLD correlates of empathy. First, we performed whole-brain analyses for contrasts approximating those used in previous research (i.e., S_XX_P_POS_-S_XX_P_XX_ and S_XX_P_NEG_-S_XX_P_XX_) to examine whether viewing positive and negative feedback directed toward one’s partner was associated with activations in canonical empathy regions. Next, we conducted whole-brain analyses to investigate the BOLD responses to social feedback directed at the partner in the presence of simultaneous incongruent social feedback about the self (i.e., S_POS_P_NEG_-S_POS_P_XX_ and S_NEG_P_POS_-S_NEG_P_XX_ contrasts). Finally, we further investigated the disparities between the incongruent feedback contrast vs. partner-alone feedback contrasts (i.e., [S_POS_P_NEG_-S_POS_P_XX_]-[S_XX_P_NEG_-S_XX_P_XX_] or [S_NEG_P_POS_-S_NEG_P_XX_]-[S_XX_P_POS_-S_XX_P_XX_]) to investigate the effect of incongruent self-directed feedback on spontaneous responses to partner-directed feedback. For these analyses, a voxel-wise threshold of *z* > 3.1 (*p* < 0.001) combined with a cluster level threshold of *p*_*GRF*_ < 0.05 was used. As a post-hoc control analysis, considering variability across participants in belief in our cover story, or age and relationship duration, we reran the whole-brain analyses using participants’ reported belief, or age and relationship duration as covariates.

#### Relationships between brain activity, affective responses, trait empathy, difficulties in emotion regulation, and daily empathic behavior

We next investigated whether variability in brain activity during the incongruent feedback contrast involving the greatest demand for regulating responses to self-directed feedback (S_NEG_P_POS_-S_NEG_P_XX_) was related to individual differences in affective ratings, emotional similarity with the partner, inferred partner-affect, trait empathy, difficulties in emotion regulation, and real-world empathic behavior. Primary analyses focused on activity in spherical regions of interest (ROIs) (radius = 6 mm), masked to include voxels within the brain, spanning the AI, dACC, TPJ, and DMPFC, which are typically found in empathy literature^10–12^, the middle frontal gyrus (MFG), associated with emotion regulation^39^, and right SMG, identified as crucial for overcoming emotional egocentricity bias^21^. ROIs within the brain were defined based on the peak Montreal Neurological Institute coordinates obtained using the terms “empathy” and “emotion regulation” from Neurosynth (https://neurosynth.org), as well as the coordinates (68, -38, 36) reported by Silani et al. for overcoming emotional egocentricity bias^21^. We extracted values from individualized z-statistic maps from these ROIs and then standardized BOLD activity and behavioral data across subjects. Linear mixed-effect models (participants nested within couples) with a random intercept yielded standardized coefficients predicting affective responses, trait empathy, difficulties in emotion regulation, and partner-rated daily support provided to the partner. Model assumptions were evaluated using simulation-based residual diagnostics implemented in the DHARMa package, including tests of residual uniformity and dispersion. While most models showed no major violations of model assumptions, deviations from residual uniformity were observed in several cases. For these models, bootstrap-based confidence intervals and *p* values (5000 iterations) were additionally estimated to assess the robustness of the results.

Secondary exploratory analyses involving whole-brain multiple regression analyses allowed us to examine whether regions beyond our hypothesized ROIs were associated with affect-, empathy- and emotion regulation-related measurements. To provide more comprehensive information, we reported results using two multiple-comparison correction approaches: (1) a voxel-wise threshold of *z* > 2.58 (*p* < 0.005) combined with a cluster size threshold of ≥ 15 voxels, and (2) a voxel-wise threshold of z > 3.1 (*p* < 0.001) combined with cluster-level GRF correction (*p*_*GRF*_ < 0.05), given that statistical models estimating between-person effects are generally more underpowered than those used for within-subject contrasts based on group-averaged responses^49^.

### Preregistration statement

This study was not preregistered.

### Reporting summary

Further information on research design is available in the Nature Portfolio Reporting Summary linked to this article.

## Results

### Behavioral results

#### Affective ratings were associated with inferred partner affect and trait empathy

Behavioral data acquired from the CLOSE task provides converging evidence that the task was successful at evoking spontaneous empathy. We first examined participants’ self-reported affect associated with partner-alone feedback contrasts commonly used in previous research. As expected, linear mixed-effects models showed that participants reported more positive affect in the partner-positive feedback condition than the no feedback condition (S_XX_P_POS_-S_XX_P_XX_, *B* = 0.58, *SE* = 0.05*, t*_(130)_ = 12.91, 95% CI: [0.494, 0.671], *p* < 0.001, *d* = 1.19), and more negative affect in the partner-negative condition than the no feedback condition (S_XX_P_NEG_-S_XX_P_XX_*, B* = -0.57, *SE* = 0.05*, t*_(130)_ = -11.69, bootstrapped 95% CI: [-0.670, -0.479], bootstrapped *p* < 0.001, *d* = -1.12) (Fig. 1B).

Next, we investigated how individuals responded emotionally to feedback directed at their partner, while simultaneously receiving conflicting feedback about themselves. Participants reported feeling more positive when self-negative feedback was accompanied by partner-positive feedback versus experienced alone (S_NEG_P_POS_-S_NEG_P_XX_: *B* = 0.40, *SE* = 0.05, *t*_(130)_ = 8.41, 95% CI: [0.310, 0.498], *p* < 0.001, *d* = 0.64) and more negative when self-positive feedback was accompanied by partner-negative feedback versus experienced alone (S_POS_P_NEG_-S_POS_P_XX_: *B* = -0.59, *SE* = 0.05, *t*_(130)_ = -11.22, 95% CI: [-0.695, -0.488], *p* < 0.001, *d* = -1.03) (Fig. 1B).

As a preliminary test of cognitive process during the paradigm, we also investigated whether participants’ affective responses during the task were associated with how they believed their partner felt when observing their own feedback (i.e., post-scan inferred partner affect; Supplementary Fig. 1). Self-reported affect in the S_XX_P_POS_ and S_XX_P_NEG_ condition during scanning was significantly positively correlated with post-scan inferred partner affect in the S_XX_P_POS_ and S_XX_P_NEG_ condition, respectively (S_XX_P_POS_: *β* = 0.27, *SE* = 0.09, *t*_(127)_ = 3.20, 95% CI = [0.106, 0.441], *p* = 0.002, partial *η²* = 0.07; S_XX_P_NEG_: *β* = 0.26, *SE* = 0.09, *t*_(127)_ = 3.06, 95% CI = [0.095, 0.430], *p* = 0.003, partial *η²* = 0.07; Fig. 1C). In other words, when participants felt that their partner felt worse (or better) across trials, participants felt correspondingly worse (or better). However, consistent with the hypothesized interference effect of simultaneous feedback trials, we didn’t find significant associations between affective responses in the S_NEG_P_POS_ (*β* = 0.07, *SE* = 0.09, *t*_(126.29)_ = 0.84, 95% CI = [-0.098, 0.247], *p* = 0.400, partial *η²* = 0.006) or S_POS_P_NEG_ condition (*β* = 0.11, *SE* = 0.09, *t*_(127)_ = 1.23, 95% CI = [-0.065, 0.281], *p* = 0.221, partial *η²* = 0.01) during scanning and post-scan inferred partner affect in the S_XX_P_POS_ or S_XX_P_NEG_ condition.

Finally, we computed a metric of affective sharing (or emotional similarity) for each participant, defined as the negative absolute difference between participants’ own affect ratings and their partner’s affect ratings for the corresponding partner-directed feedback condition. For example, we calculated the differences between participants’ own ratings for S_XX_P_NEG_ and S_POS_P_NEG_ (or S_XX_P_POS_ and S_NEG_P_POS_) and their partner’s ratings when their partner observes S_NEG_P_XX_ or S_POS_P_XX_, respectively (Fig. 1D). This metrics reflects the degree to which participants’ feelings during the CLOSE task aligns with their partner’s *actual feelings* during corresponding feedback conditions. Relatedly, we examined whether individual differences in emotional similarity with one’s partner were associated with scores on EC_IRI, reflecting the affective component of empathy^43^. Results showed that emotional similarity with one’s partner in the S_XX_P_POS_ condition (*β* = 0.21, *SE* = 0.09, *t*_*(*126)_ = 2.42, 95% CI = [0.040, 0.382], *p* = 0.017, partial *η²* = 0.04; Fig. 1E), but not the S_XX_P_NEG_ condition (*β* = 0.01, *SE* = 0.09, *t*_(126)_ = 0.16, 95% CI = [-0.160, 0.189], *p* = 0.874, partial *η²* < 0.001), was positively associated with scores on EC_IRI. That is, individuals with higher empathic concern tended to show greater emotional similarity with their partner’s feelings when their partner received positive feedback. We also conducted exploratory analyses examining the relationship between scores on perspective taking subscale of the Interpersonal Reactivity Index (PT_IRI) and emotional similarity. These analyses did not reveal significant associations for both S_XX_P_POS_ (*β* = 0.06, *SE* = 0.09, *t*_(126)_ = 0.66, 95% CI = [−0.115, 0.233], *p* = 0.508, partial *η²* = 0.003) and S_XX_P_NEG_ condition (*β* = 0.06, *SE* = 0.09, *t*_(126)_ = 0.710, 95% CI = [−0.111, 0.237], *p* = 0.479, partial *η²* = 0.004).

#### Self-directed feedback reduced affective responses to partner-directed feedback

We further examined whether simultaneous incongruent self-directed feedback interfered with spontaneous responses to partner-directed feedback, by comparing incongruent feedback contrasts with partner-alone feedback contrasts. Participants’ affect was less improved by positive partner-directed feedback when participants themselves received negative feedback, compared to when negative self-directed feedback was absent ([S_NEG_P_POS_-S_NEG_P_XX_]-[S_XX_P_POS_-S_XX_P_XX_], *B* = −0.18, *SE* = 0.04, *t*_(130)_ = −4.10, 95% CI: [−0.264, −0.093], *p* < 0.001, *d* = −0.34). However, participants reported similar decreases in affect when their partners received negative feedback, irrespective of whether they received positive or no feedback ([S_POS_P_NEG_-S_POS_P_XX_]-[S_XX_P_NEG_-S_XX_P_XX_], *B* = -0.02, *SE* = 0.04, *t*_(130)_ = −0.39, 95% CI: [−0.103, 0.068], *p* = 0.695, *d* = −0.03). These findings suggest that the presence of negative self-relevant feedback may be particularly disruptive for empathy.

Supporting these findings, emotional similarity with participants’ partners was significantly lower in the S_NEG_P_POS_ than the S_XX_P_POS_ condition (*β* = −0.26, *SE* = 0.05, *t*_(127)_ = −4.89, bootstrapped 95% CI = [−0.357, −0.154], bootstrapped *p* < 0.001, *d* = −0.46), and in the S_POS_P_NEG_ than the S_XX_P_NEG_ condition (*β* = −0.12, *SE* = 0.05, *t*_(127)_ = −2.38, bootstrapped 95% CI = [−0.223, −0.022], bootstrapped *p* = 0.016, *d* = −0.19; Fig. 1D). As a final analysis, we investigated whether affective sharing with a partner during incongruent feedback conditions was linked to self-reported emotion regulation tendencies. Results showed that emotional similarity with one’s partner in the S_NEG_P_POS_ condition was negatively associated with total score on DERS-18 (*β* = −0.28, *SE* = 0.09, *t*_(126)_ = −3.19, 95% CI = [−0.448, −0.107], *p* = 0.002, partial *η²* = 0.07; Fig. 1E), indicating that individuals with higher difficulties in emotion regulation showed lower affective sharing with their partner during the S_NEG_P_POS_ condition. To further clarify which aspects of emotion regulation were most closely linked to this effect, we conducted exploratory analyses on DERS-18 subscales. Significant associations emerged for the Goals (*β* = −0.29, *SE* = 0.09, *t*_(116.08)_ = −3.36, 95% CI = [−0.455, −0.120], *p* = 0.001, *p*_FDR_ = 0.006, partial *η²* = 0.09), Impulse (*β* = −0.24, *SE* = 0.09, *t*_(126)_ = −2.70, bootstrapped 95% CI = [−0.409, −0.066], bootstrapped *p* = 0.007, *p*_FDR_ = 0.014, partial *η²* = 0.05), and Strategies subscales (*β* = −0.25, *SE* = 0.09, *t*_(119.96)_ = −2.84, bootstrapped 95% CI = [−0.419, −0.075], bootstrapped *p* = 0.004, *p*_FDR_ = 0.012, partial *η²* = 0.06), but not the Awareness (*β* = −0.04, *SE* = 0.09, *t*_(124)_ = −0.43, 95% CI = [−0.215, 0.137], *p* = 0.666, *p*_FDR_ = 0.686, partial *η²* = 0.002), Clarity (*β* = −0.04, *SE* = 0.09, *t*_(125.61)_ = −0.43, bootstrapped 95% CI = [−0.213, 0.141], bootstrapped *p* = 0.686, *p*_FDR_ = 0.686, partial *η²* = 0.001), and Nonacceptance subscales (*β* = −0.17, *SE* = 0.09, *t*_(125.05)_ = −1.93, 95% CI = [−0.337, 0.002], *p* = 0.056, *p*_FDR_ = 0.083, partial *η²* = 0.03).

### fMRI results

#### Partner-alone feedback contrasts recruited regions canonically implicated in empathy

**Fig. 2:**
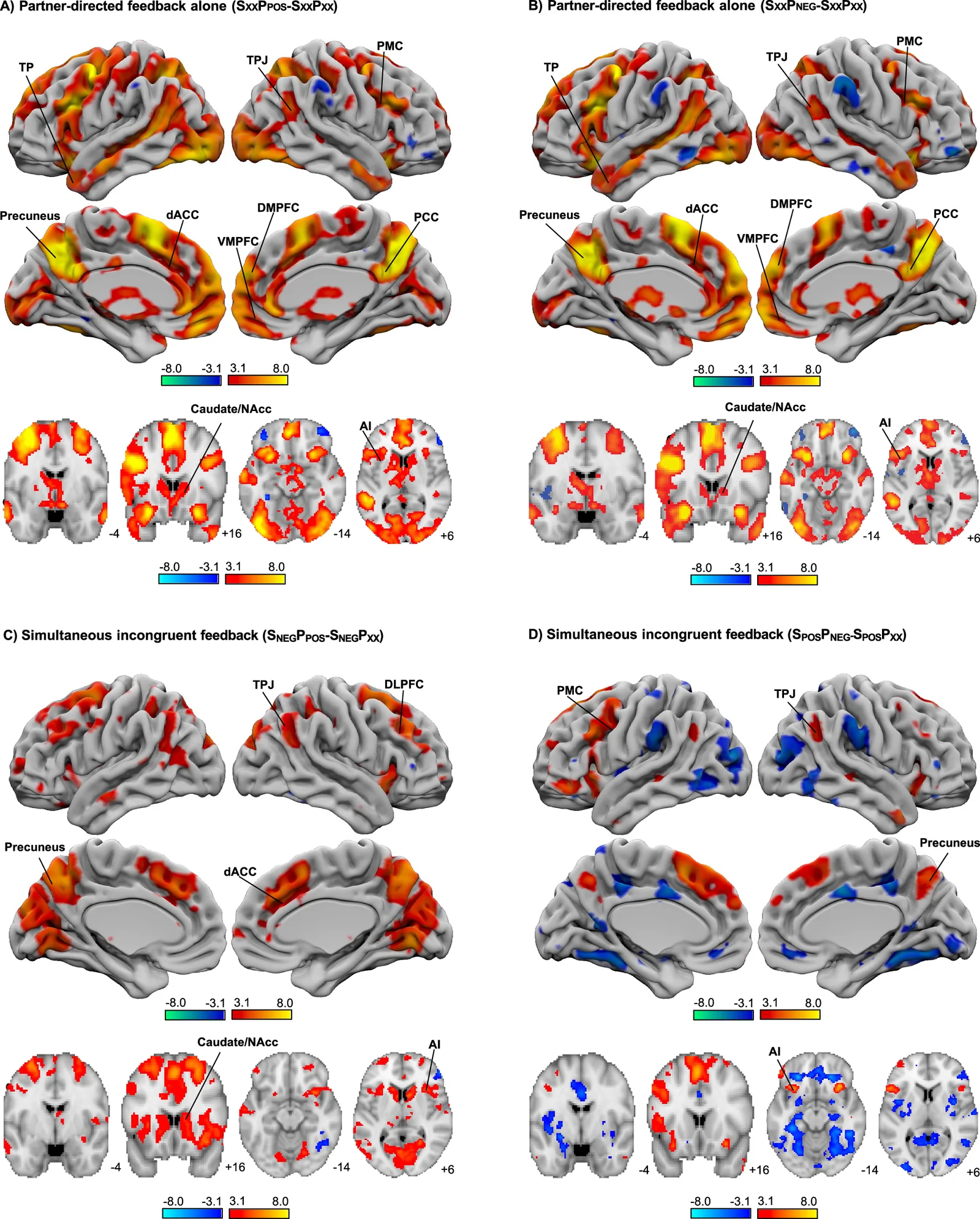
Spontaneous brain responses to partner-directed feedback in the absence and presence of simultaneous incongruent self-directed feedback. **A** BOLD responses to positive partner-directed feedback alone (S_XX_P_POS_-S_XX_P_XX_ contrast). **B** BOLD responses to negative partner-directed feedback alone (S_XX_P_NEG_-S_XX_P_XX_ contrast). **C** BOLD responses to positive partner-directed feedback, in the presence of negative self-directed feedback (S_NEG_P_POS_-S_NEG_P_XX_ contrast). **D** BOLD responses to negative partner-directed feedback, in the presence of positive self-directed feedback (S_POS_P_NEG_-S_POS_P_XX_ contrast). Per recommendation from Surf Ice (https://www.nitrc.org/projects/surfice/), inflated brain images are shown at a voxel-wise threshold of *z* > 3.1 without cluster correction, whereas slice views additionally employ a cluster level threshold of *p*_*GRF*_ < 0.05. All images are displayed in neurological orientation. TP: temporal pole; dACC: dorsal anterior cingulate cortex; TPJ: temporoparietal junction; PMC: premotor cortex; DMPFC: dorsomedial prefrontal cortex; VMPFC: ventromedial prefrontal cortex; PCC: posterior cingulate cortex; NAcc: nucleus accumbens; AI: anterior insula; DLPFC: dorsolateral prefrontal gyrus.

Beyond behavioral evidence that the CLOSE task elicited empathy, we also conducted preliminary analyses to examine whether observing feedback about one’s partner alone elicited brain patterns observed in prior empathy research, including those canonically associated with affective sharing, mentalizing, and empathic concern. Consistent with our hypotheses, both S_XX_P_POS_-S_XX_P_XX_ and S_XX_P_NEG_-S_XX_P_XX_ contrasts yielded BOLD activity in broad regions found in the empathy literature, including the dACC, AI, premotor cortex, TPJ, DMPFC, PCC, ventromedial prefrontal cortex, and temporal pole (*z* > 3.1 and cluster level *p*_GRF_ < 0.05; Fig. 2A–B, and Supplementary Table 2).

#### Simultaneous incongruent feedback contrasts additionally recruited frontoparietal regions with attenuated activity in regions canonically associated with empathy

Next, we examined spontaneous BOLD activity patterns in incongruent feedback contrasts, when partner-directed feedback was accompanied by simultaneous incongruent self-directed feedback (i.e., S_NEG_P_POS_-S_NEG_P_XX_ and S_POS_P_NEG_-S_POS_P_XX_). Although we observed less widespread BOLD activity in these contrasts in many regions mentioned above, the incongruent feedback contrasts yielded additional BOLD activity in regions throughout the frontoparietal control network, including the MFG/DLPFC and SMG/anterior inferior parietal lobule (*z* > 3.1 and cluster level *p*_*GRF*_ < 0.05; Table 1 and Fig. 2C–D). To control for individual differences in participants’ belief in our cover story, we conducted another analysis including this metric from the study debriefing as a covariate. The main results remained unchanged after regressing the ratings of belief in our cover story (Supplementary Table 3). Similarly, these results remained unchanged after controlling for participants’ age and relationship duration (Supplementary Table 4).

**Table 1:** Brain responses to partner-directed feedback in the presence of simultaneous incongruent self-directed feedback

Contrast	Voxels	*Z value*	X	Y	Z	*p*_(cluster-GRF)_	Brain region
S_NEG_P_POS_ > S_NEG_P_XX_	25984	7.47	−4	−58	50	< 0.001	Precuneus Cortex
		7.41	−4	−60	54		Precuneus Cortex
		7.16	−2	−70	56		Precuneus Cortex
		7.03	−2	−62	58		Precuneus Cortex
		7.02	−2	−66	48		Precuneus Cortex
		6.58	2	14	54		Superior Frontal Gyrus
	2802	5.72	36	14	−12	< 0.001	Insular Cortex
		5.31	10	12	6		Right Caudate
		5.27	38	18	−6		Insular Cortex
		5.16	48	20	0		Frontal Operculum Cortex
		5.14	42	20	−4		Frontal Orbital Cortex
		5.08	−18	20	−2		Left Caudate
	2480	5.41	42	−44	42	< 0.001	Posterior Supramarginal Gyrus
		5.15	60	−60	28		Superior Lateral Occipital Cortex
		4.96	56	−60	30		Superior Lateral Occipital Cortex
		4.88	52	−58	28		Angular Gyrus
		4.82	38	−40	34		Posterior Supramarginal Gyrus
		4.80	56	−64	34		Superior Lateral Occipital Cortex
	418	4.69	−10	−24	−4	< 0.001	Left Thalamus
		4.34	−14	−20	14		Left Thalamus
		4.27	4	−22	0		Right Thalamus
		4.23	−14	−32	8		Left Thalamus
		4.15	−22	−28	0		Left Cerebral White Matter
		4.05	12	−18	14		Right Thalamus
	339	4.38	−26	56	16	< 0.001	Frontal Pole
		4.37	−30	64	12		Frontal Pole
		4.26	−28	58	8		Frontal Pole
		4.14	−22	64	2		Frontal Pole
	317	4.77	−60	−8	−16	< 0.001	Anterior Middle Temporal Gyrus
		4.48	−64	−12	−20		Posterior Middle Temporal Gyrus
		4.06	−62	−2	−22		Anterior Middle Temporal Gyrus
		3.96	−66	−10	−8		Anterior Middle Temporal Gyrus
		3.79	−64	−12	−26		Posterior Middle Temporal Gyrus
		3.63	−66	−16	−8		Posterior Middle Temporal Gyrus
	177	4.69	2	40	0	0.003	Anterior Cingulate Gyrus
		4.03	−2	30	12		Anterior Cingulate Gyrus
		3.86	4	34	10		Anterior Cingulate Gyrus
	129	4.19	64	−14	10	0.014	Central Opercular Cortex
		3.96	52	−4	8		Central Opercular Cortex
		3.94	52	−8	6		Central Opercular Cortex
		3.61	64	−4	8		Central Opercular Cortex
		3.26	56	−12	4		Heschl’s Gyrus (includes H1 and H2)
		3.21	62	6	12		Precentral Gyrus
	122	4.22	−50	44	−12	0.019	Frontal Pole
		3.93	−40	42	−8		Frontal Pole
		3.73	−46	44	−6		Frontal Pole
		3.58	−36	46	−2		Frontal Pole
	115	4.45	6	62	2	0.024	Frontal Pole
		4.06	4	68	2		Frontal Pole
S_NEG_P_POS_ < S_NEG_P_XX_	244	4.71	40	−42	−18	< 0.001	Temporal Occipital Fusiform Cortex
		4.42	46	−48	−22		Temporal Occipital Fusiform Cortex
		4.27	46	−44	−22		Temporal Occipital Fusiform Cortex
		4.18	46	−56	−16		Temporal Occipital Fusiform Cortex
		4.17	50	−56	−16		Inferior Temporal Gyrus
		4.08	40	−58	−16		Temporal Occipital Fusiform Cortex
	173	5.41	52	44	8	0.003	Frontal Pole
		3.95	54	36	18		Frontal Pole
		3.80	52	36	−2		Frontal Pole
		3.77	46	36	8		Frontal Pole
S_POS_P_NEG_ > S_POS_P_XX_	3369	6.09	−52	20	2	< 0.001	Inferior Frontal Gyrus
		5.95	−30	20	−12		Frontal Orbital Cortex
		5.92	−40	20	30		Middle Frontal Gyrus
		5.85	−46	46	−8		Frontal Pole
		5.82	−50	20	36		Middle Frontal Gyrus
		5.71	−36	22	−14		Frontal Orbital Cortex
	2892	6.79	−2	14	52	< 0.001	Paracingulate Gyrus
		6.36	−8	36	56		Superior Frontal Gyrus
		5.43	−4	28	40		Paracingulate Gyrus
		5.38	−2	56	28		Superior Frontal Gyrus
		5.27	−6	48	50		Frontal Pole
		5.24	−8	22	42		Paracingulate Gyrus
	972	5.37	−6	−62	46	< 0.001	Precuneus Cortex
		5.11	2	−66	48		Precuneus Cortex
		4.39	4	−60	38		Precuneus Cortex
		3.83	−10	−50	28		Posterior Cingulate Gyrus
		3.81	−10	−54	34		Precuneus Cortex
	780	5.22	56	−58	38	< 0.001	Angular Gyrus
		4.79	44	−58	44		Angular Gyrus
		4.67	38	−48	42		Superior Parietal Lobule
		4.61	46	−58	32		Angular Gyrus
		3.84	52	−64	46		Superior Lateral Occipital Cortex
		3.63	34	−44	36		Posterior Supramarginal Gyrus
	608	6.85	34	24	−12	< 0.001	Frontal Orbital Cortex
		4.86	32	28	0		Frontal Orbital Cortex
		4.68	46	20	8		Inferior Frontal Gyrus
		4.44	44	20	0		Frontal Operculum Cortex
		3.69	56	28	−6		Inferior Frontal Gyrus
	504	4.66	−40	−50	48	< 0.001	Superior Parietal Lobule
		4.62	−34	−60	44		Superior Lateral Occipital Cortex
		3.81	−34	−46	40		Superior Parietal Lobule
		3.45	−44	−40	42		Anterior Supramarginal Gyrus
		3.44	−48	−40	44		Anterior Supramarginal Gyrus
		3.41	−42	−62	58		Superior Lateral Occipital Cortex
	289	5.54	54	10	−30	< 0.001	Temporal Pole
		4.49	58	0	−30		Anterior Middle Temporal Gyrus
	284	4.51	−54	−58	30	< 0.001	Angular Gyrus
		4.39	−46	−58	34		Angular Gyrus
		3.85	−56	−60	40		Superior Lateral Occipital Cortex
		3.71	−52	−60	50		Superior Lateral Occipital Cortex
		3.60	−50	−58	54		Angular Gyrus
	241	4.82	−56	−32	0	< 0.001	Posterior Superior Temporal Gyrus
		4.57	−52	−30	−4		Posterior Middle Temporal Gyrus
		3.51	−48	−38	−4		Posterior Middle Temporal Gyrus
	226	6.09	56	−32	−4	0.001	Posterior Middle Temporal Gyrus
		3.64	50	−22	−12		Posterior Middle Temporal Gyrus
	215	4.84	48	30	38	0.001	Middle Frontal Gyrus
		3.94	52	22	40		Middle Frontal Gyrus
		3.63	52	34	28		Middle Frontal Gyrus
	123	4.28	−48	2	−36	0.022	Temporal Pole
		4.15	−44	2	−38		Temporal Pole
		3.75	−50	6	−30		Temporal Pole
S_POS_P_NEG_ < S_POS_P_XX_	4453	6.54	30	−58	−8	< 0.001	Temporal Occipital Fusiform Cortex
		6.36	30	−38	−18		Posterior Temporal Fusiform Cortex
		5.98	26	−66	−12		Occipital Fusiform Gyrus
		5.95	50	−44	−18		Inferior Temporal Gyrus
		5.85	38	−84	16		Superior Lateral Occipital Cortex
		5.85	30	−44	−10		Temporal Occipital Fusiform Cortex
	2948	6.43	−48	−60	0	< 0.001	Middle Temporal Gyrus
		6.21	−32	−34	−16		Posterior Parahippocampal Gyrus
		5.76	−56	−66	−4		Inferior Lateral Occipital Cortex
		5.46	−30	−46	−8		Temporal Occipital Fusiform Cortex
		5.45	−28	−46	−20		Temporal Occipital Fusiform Cortex
		5.42	−26	−64	−10		Occipital Fusiform Gyrus
	1955	6.21	−8	−34	44	< 0.001	Posterior Cingulate Gyrus
		5.45	16	−36	42		Precuneus Cortex
		5.38	6	−36	42		Posterior Cingulate Gyrus
		5.26	8	−38	52		Precuneus Cortex
		5.18	−10	−54	68		Superior Parietal Lobule
		4.99	10	−32	44		Posterior Cingulate Gyrus
	1573	5.80	−66	−30	30	< 0.001	Anterior Supramarginal Gyrus
		5.48	−60	−30	22		Parietal Operculum Cortex
		5.07	−34	6	10		Insular Cortex
		4.90	−36	2	10		Insular Cortex
		4.89	−58	−16	16		Central Opercular Cortex
		4.84	−42	2	6		Central Opercular Cortex
	1174	5.64	64	−22	30	< 0.001	Anterior Supramarginal Gyrus
		5.39	62	−22	22		Anterior Supramarginal Gyrus
		5.31	64	−28	46		Anterior Supramarginal Gyrus
		5.29	62	−30	40		Anterior Supramarginal Gyrus
		4.73	64	−18	46		Postcentral Gyrus
		4.56	66	−14	8		Planum Temporale
	1173	6.22	−34	−88	16	< 0.001	Superior Lateral Occipital Cortex
		5.15	−26	−88	16		Superior Lateral Occipital Cortex
		4.78	−32	−90	2		Inferior Lateral Occipital Cortex
		4.53	−42	−78	24		Superior Lateral Occipital Cortex
		4.37	−20	−74	44		Superior Lateral Occipital Cortex
		4.34	−38	−82	26		Superior Lateral Occipital Cortex
	977	6.57	−26	36	−14	< 0.001	Frontal Orbital Cortex
		5.88	22	36	−14		Frontal Pole
		5.56	−32	38	−6		Frontal Pole
		5.12	2	44	−12		Frontal Medial Cortex
		4.73	−6	38	−14		Frontal Medial Cortex
		4.66	32	36	−16		Frontal Pole
	905	6.01	−8	−58	12	< 0.001	Precuneus Cortex
		4.78	12	−50	10		Precuneus Cortex
		4.71	8	−58	16		Precuneus Cortex
		4.45	6	−56	4		Lingual Gyrus
		4.32	18	−44	2		Posterior Cingulate Gyrus
		4.17	−4	−46	6		Posterior Cingulate Gyrus
	625	6.78	2	4	38	< 0.001	Anterior Cingulate Gyrus
		3.79	−16	10	32		Left Cerebral White Matter
	298	4.68	22	0	−12	< 0.001	Right Cerebral Cortex
		4.38	36	−16	4		Insular Cortex
		4.20	40	−8	−4		Insular Cortex
		3.89	26	−10	4		Right Putamen
		3.88	30	−16	−2		Right Putamen
		3.68	30	−6	−8		Right Putamen
	215	4.67	−46	−28	64	0.001	Postcentral Gyrus
		4.09	−38	−24	68		Postcentral Gyrus
		3.64	−30	−28	74		Postcentral Gyrus
		3.56	−32	−26	58		Precentral Gyrus
		3.49	−40	−40	66		Postcentral Gyrus
	199	4.76	32	−42	74	0.002	Superior Parietal Lobule
		4.43	50	−28	62		Postcentral Gyrus
		4.11	30	−46	66		Superior Parietal Lobule
		3.96	28	−38	60		Postcentral Gyrus
		3.63	40	−32	66		Postcentral Gyrus
		3.60	24	−42	74		Superior Parietal Lobule
	194	4.31	28	−20	−12	0.002	Right Hippocampus
		4.23	42	−12	−28		Posterior Temporal Fusiform Cortex
		4.11	34	−14	−20		Anterior Parahippocampal Gyrus
		4.04	22	−20	−14		Posterior Parahippocampal Gyrus
		3.67	36	−8	−38		Anterior Temporal Fusiform Cortex
		3.37	18	−22	−20		Anterior Parahippocampal Gyrus
	191	5.03	38	2	4	0.002	Insular Cortex
		4.18	34	10	10		Insular Cortex
	169	5.23	52	40	12	0.004	Frontal Pole
	144	4.70	18	−54	64	0.010	Superior Parietal Lobule
		4.08	16	−50	64		Superior Parietal Lobule
		4.02	18	−46	64		Superior Parietal Lobule
	121	4.93	22	−74	46	0.023	Superior Lateral Occipital Cortex
		3.92	26	−72	38		Superior Lateral Occipital Cortex

**Fig. 3:**
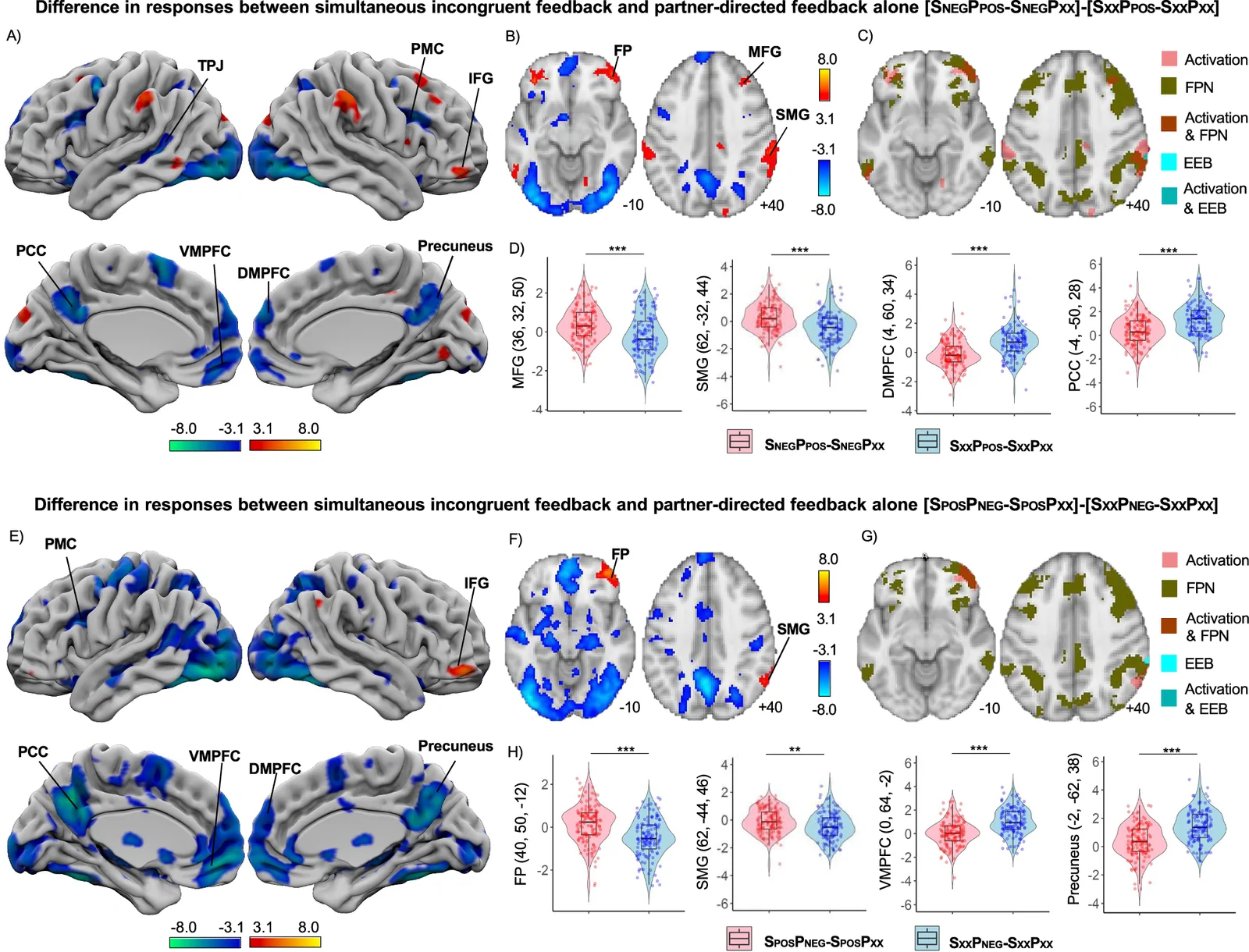
Observing partner-directed feedback while simultaneous receiving incongruent self-directed feedback elicits less activation in canonical empathy regions but greater activation in frontoparietal regions, compared to when no simultaneous incongruent self-directed feedback is present. **A**–**D** Differences between BOLD responses to positive partner-directed feedback in the presence of negative self-directed feedback (S_NEG_P_POS_-S_NEG_P_XX_) and BOLD responses to positive partner-directed feedback alone (S_XX_P_POS_-S_XX_P_XX_). **E**–**H** Differences between BOLD responses to negative partner-directed feedback in the presence of positive self-directed feedback (S_POS_P_NEG_-S_POS_P_XX_) and BOLD responses to negative partner-directed feedback alone (S_XX_P_NEG_-S_XX_P_XX_). **A**, **E** Inflated brain images are displayed at a voxel-wise threshold of z > 3.1 without cluster correction, as recommended by Surf Ice (https://www.nitrc.org/projects/surfice/). **B**, **F** Slice views are presented at a voxel-wise threshold of z > 3.1, with a cluster-level threshold of *p*_GRF_ < .05. All images are shown in neurological orientation. **C**, **G** Comparisons between activation patterns and Yeo’s frontoparietal network^44^, as well as the supramarginal gyrus implicated in overcoming emotional egocentricity bias (x = 68, y = -38, z = 36, radius = 6 mm) from Silani et al.’s work^21^. **D**, **H** Plots visualize extracted signals from spherical ROIs based on the identified coordinates of representative brain regions (*N* = 131). The broader shape of the violin plots illustrates the data distribution, with wider sections indicating higher density. The nested box plots display the median line (center line), quartiles (box edges representing the 25th and 75th percentiles), and whiskers extending to 1.5 × the interquartile range (IQR). ***p < .001; **p < .01. PCC: posterior cingulate cortex; TPJ: temporoparietal junction; DMPFC: dorsomedial prefrontal cortex; VMPFC: ventromedial prefrontal cortex; PMC: premotor cortex; IFG: inferior frontal gyrus; FP: frontal pole; MFG: middle frontal gyrus; SMG: supramarginal gyrus; FPN: Frontoparietal network; EEB: overcoming emotional egocentricity bias.

As a follow-up analysis to better understand the unique activity pattern observed in the incongruent feedback contrasts, we compared BOLD activity between incongruent feedback and partner-alone feedback contrasts. The incongruent feedback contrasts, compared to partner-alone feedback contrasts, yielded statistically greater BOLD activity in frontoparietal areas including right frontal pole, inferior frontal gyrus, MFG/DLPFC and bilateral SMG (*z* > 3.1 and cluster level *p*_*GRF*_ < 0.05; Fig. 3 and Supplementary Table 5). This activity pattern overlaps with Yeo et al.’s frontoparietal control network^50^ and the right SMG that Silani et al. identified in overcoming emotional egocentricity bias^21^ (Figs. 3C and 3G). Notably, this pattern was more pronounced in the S_NEG_P_POS_-S_NEG_P_XX_ compared to the S_XX_P_POS_-S_XX_P_XX_ contrast. Conversely, activity in regions including left TPJ, bilateral PCC, bilateral DMPFC, bilateral AI, bilateral premotor cortex/precentral gyrus, and bilateral pre-supplementary motor area were reduced in incongruent feedback compared to partner-alone feedback contrasts (Fig. 3 and Supplementary Table 5).

#### BOLD activity in the S_NEG_P_POS_-S_NEG_P_XX_ contrast was associated with trait emotion regulation, inferred partner affect, and empathic concern

To examine sources of between-subject variability in BOLD activity during the incongruent feedback contrast, we conducted exploratory analyses examining relationships between BOLD activity in the S_NEG_P_POS_-S_NEG_P_XX_ contrast involving the greatest demand for regulating responses to self-directed feedback and multiple measures: affective ratings in S_NEG_P_POS_-S_NEG_P_XX_ contrast, inferred partner affect for positive partner-directed feedback, emotional similarity in S_NEG_P_POS_ condition, as well as trait scores assessing perspective taking (PT_IRI), empathic concern (EC_IRI), and emotion regulation (DERS-18). We first conducted a region of interest (ROI) analysis focusing on four ROIs (DMPFC, right TPJ, left ACC, right AI) identified using the search term “empathy,” one ROI in the left MFG identified using the search term “emotion regulation” from a Neurosynth meta-analysis (https://neurosynth.org), and one ROI in the right SMG, identified as crucial for overcoming emotional egocentricity bias^21^ (Supplementary Table 6).

**Fig. 4:**
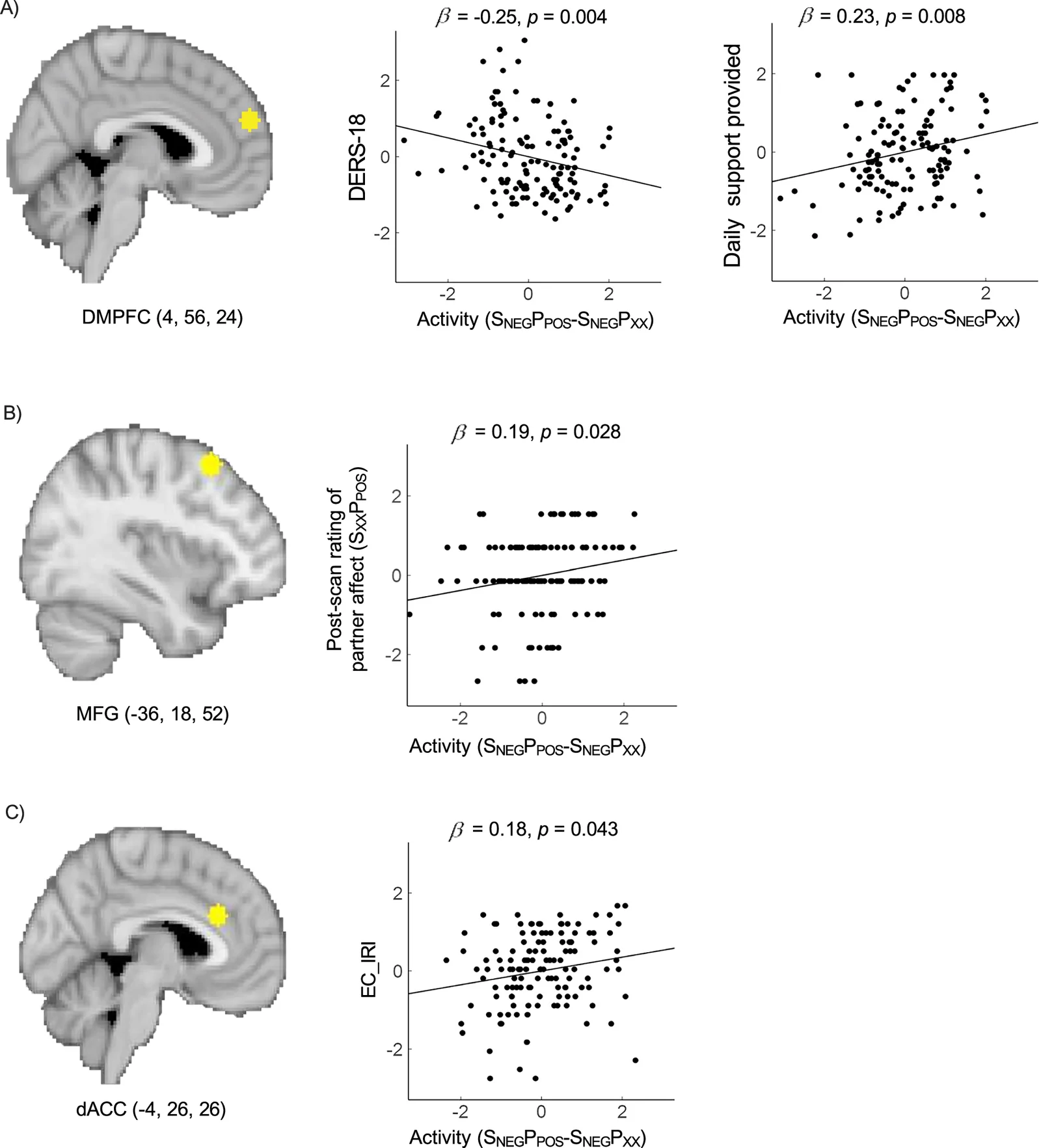
Relationships between spontaneous brain responses to positive partner-directed feedback in the presence of negative self-directed feedback (S_NEG_P_POS_-S_NEG_P_XX_), emotion regulation tendencies, daily support provided to the partner, inferred partner affect, and trait empathic concern. **A** Greater activity in the dorsomedial prefrontal cortex (DMPFC) during the S_NEG_P_POS_-S_NEG_P_XX_ contrast was associated with lower scores on difficulties in emotion regulation scale (DERS-18; left) and higher partner-rated daily support provided to the partner (right; *N* = 131). **B** Greater activity in the middle frontal gyrus (MFG) during the S_NEG_P_POS_-S_NEG_P_XX_ contrast was associated with higher post-scan inferred partner affect for positive partner-directed feedback (S_XX_P_POS_) (*N* = 129). Note that the *p* value displayed in the plot was derived via bootstrapping (5000 iterations) due to deviation from model residual assumptions. **C** Greater activity in the dorsal anterior cingulate cortex (dACC) during the S_NEG_P_POS_-S_NEG_P_XX_ contrast was associated with higher scores on empathic concern subscale of the Interpersonal Reactivity Index (EC_IRI) (*N* = 131).

Higher scores on the DERS-18 were associated with lower DMPFC activity (*β* = −0.25, *SE* = 0.08, *t*_(123.92)_ = −2.97, 95% CI = [−0.410, -0.084], *p* = 0.004, partial *η²* = 0.07; Fig. 4A left), an effect that survived FDR correction (*p*_FDR_ = 0.022). Said differently, participants with lower difficulties in emotion regulation engaged the DMPFC to a greater degree during the S_NEG_P_POS_-S_NEG_P_XX_ contrast. Of note, the Empathy Assessment Index (EAI) was also used for its conceptual alignment with our hypotheses. The associations between scores on the emotion regulation subscale of the EAI and DMPFC activity paralleled the findings reported here; the details are available in the Supplementary Note 1.

To further clarify which aspects of emotion regulation were most closely linked to this effect, we conducted exploratory analyses on DERS-18 subscales. Significant associations emerged for the Goals (*β* = -0.20, *SE* = 0.09, *t*_(129)_ = -2.34, 95% CI = [−0.371, −0.033], *p* = 0.021, *p*_FDR_ = 0.041, partial *η²* = 0.04), Awareness (*β* = −0.22, *SE* = 0.08, *t*_(126.33)_ = −2.59, 95% CI = [−0.384, −0.053], *p* = 0.011, *p*_FDR_ = 0.032, partial *η²* = 0.05), Strategies subscales (*β* = −0.22, *SE* = 0.09, *t*_(129)_ = −2.55, bootstrapped 95% CI = [−0.387, −0.049], bootstrapped *p* = 0.009, *p*_FDR_ = 0.032, partial *η²* = 0.05), but not the Nonacceptance (*β* = −0.17, *SE* = 0.08, *t*_(112.98)_ = −2.14, 95% CI = [−0.329, −0.015], *p* = 0.034, *p*_FDR_ = 0.052, partial η² = 0.04), Impulse (*β* = −0.14, *SE* = 0.09, *t*_(129)_ = −1.64, bootstrapped 95% CI = [−0.315, 0.030], bootstrapped *p* = 0.101, *p*_FDR_ = 0.102, partial *η²* = 0.02) and Clarity subscales (*β* = −0.14, *SE* = 0.08, *t*_(121.81)_ = −1.63, bootstrapped 95% CI = [−0.297, 0.029], bootstrapped *p* = 0.102, *p*_FDR_ = 0.102, partial *η²* = 0.02).

Additional results described below do not survive multiple comparisons correction but may nonetheless warrant further investigation in future studies. Specifically, higher inferred partner affect for positive partner-directed feedback was associated with greater activity in the left MFG (*β* = 0.19, SE = 0.09, *t*_(125.31)_ = 2.19, bootstrapped 95% CI = [0.023, 0.366], bootstrapped *p* = 0.028, *p*_FDR_ = 0.156, partial *η*^*2*^ = 0.04; Fig. 4B). In other words, when individuals showed stronger MFG engagement to positive feedback directed at their partner—while receiving negative feedback about themselves—they tended to infer more positive affect in their partner after the partner received positive feedback. Additionally, higher scores on the EC_IRI were associated with higher activity in the dACC (*β* = 0.18, *SE* = 0.09, *t*_(129)_ = 2.05, 95% CI = [0.007, 0.347], *p* = 0.043, *p*_FDR_ = 0.256, partial *η²* = 0.03; Fig. 4C). See Supplementary Table 7 for statistics associated with all analyses.

For completeness, ROI analyses were followed by whole-brain exploratory analyses, which are displayed in Supplementary Table 8–9 and Supplementary Fig. 2.

#### BOLD activity in the S_NEG_P_POS_-S_NEG_P_XX_ contrast was associated with real-world supportive behavior

As a final analysis, we examined whether S_NEG_P_POS_-S_NEG_P_XX_ activity in the above ROIs was related to real-world empathic behavior, operationalized as support provided to one’s partner over 1-week daily diary completed by the *participants’ partners*. Higher daily support provided to the partner was associated with higher DMPFC activity (*β* = 0.23, *SE* = 0.09, *t*_(128.31)_ = 2.70, 95% CI = [0.063, 0.396], *p* = 0.008, partial *η²* = 0.05), an effect that survived FDR correction (*p*_FDR_ = 0.047; Fig. 4A right). Participants who recruited the DMPFC to a greater degree when positive feedback was directed at their partner—while receiving negative feedback about themselves—provided more support to their partners in everyday life, as reported by their partner. As with the prior set of analyses, we also conducted whole-brain exploratory analyses (Supplementary Tables 8–9 and Supplementary Fig. 2).

As an exploratory analysis, we further leveraged the dyadic nature of the study to examine the associations between BOLD responses during incongruent feedback situations and romantic relationship satisfaction. Detailed descriptions of the background, analytic approach, results, and interpretation of the Actor–Partner Interdependence Models in S_NEG_P_POS_-S_NEG_P_XX_ contrast, as well as inter-partner representational similarity analyses in S_NEG_P_POS_ condition, are provided in the Supplementary Note 2 and Supplementary Fig. 3.

## Discussion

Responding empathically in everyday life can be challenging, particularly when others’ emotional experiences conflict with one’s own mood or emotional state. These situations raise questions that are fundamental to a deeper understanding of our sociality: how do individuals navigate conflicting self- and other-related emotional information, and how does this process shape daily empathic responding? This exploratory study extends prior research by investigating spontaneous neural responses to partner-directed feedback within romantic relationships, when accompanied by incongruent self-directed feedback, and their associations with daily support provided to the partner. Notably, our study makes several contributions by examining empathy for both positive and negative partner’s social experiences, integrating laboratory-based neuroimaging with real-world behavioral assessments, and leveraging a diverse and relatively large sample of romantic partners. Results showed that when responding to partner-directed feedback paired with simultaneous incongruent self-directed feedback, participants evidenced activity in regions typically observed in empathy research, as well as frontoparietal regions frequently implicated in cognitive control, emotion regulation, and the mitigation of egocentric bias—especially when participants receive negative self-directed feedback. Recruitment of these regions was associated with greater affective empathy, higher trait empathy and lower difficulties in emotion regulation, and more frequent partner-rated daily support. These preliminary results are consistent with prior research reflecting engagement of empathic and emotion-regulatory processes^10,12,38,39^ and indicate that individual differences in emotion regulation tendencies relate to variability in spontaneous responses under conflicting social feedback situations. Notably, such spontaneous responses were also associated with real-world variability in empathic responding in romantic relationships.

During partner-alone feedback contrasts, we observed robust behavioral changes and BOLD activity in broad brain regions found in prior empathy-related investigations^10,12^. At the behavioral level, we found corresponding changes in self-reported affect for partner-directed feedback, and individuals with higher empathic concern exhibited greater alignment of emotional experiences with their partner, consistent with patterns commonly discussed in the context of empathic responding^10,12^. In addition, participants’ self-reported affective responses in the S_XX_P_POS_ or S_XX_P_NEG_ condition during scanning were positively correlated with post-scan inferred partner affect in the S_XX_P_POS_ or S_XX_P_NEG_ condition. This finding suggests that participants’ reactions to partner-directed feedback were influenced, at least in part, by inferences about their partner’s responses. Alternatively, individuals may use their own affective responses to simulate their inferred partner’s experiences, in line with self-projection^51^. At the neural level, the findings are consistent with the behavioral observations. The observed brain activity aligns with prior empathy research, which has shown that observing others’ pain or emotional experiences recruits not only brain regions involved in affective state sharing including the dACC and AI, but also regions involved in mentalizing including the TPJ, temporal pole, DMPFC, PCC, and precuneus^10,12,52^. Importantly, prior literature indicates that vicarious pain may rely upon imagining the state of the other, especially in cue-based paradigms where visual depictions of the body are absent^53,54^. Within this framework, mentalizing is widely recognized as a key pathway to empathy, as it enables individuals to imagine the condition of others, infer others’ internal states and, in turn, support empathic responses^54,55^. Taken together, while additional processes may be at play (see Limitations), these behavioral and neural findings provide strong converging evidence that our experimental task engages empathic processes within the context of social feedback^35,40^ and replicates similar neural activations observed in alternative contexts, such as those involving pain and social exclusion^2,10,12^.

During the incongruent feedback contrasts, when partner-directed feedback was accompanied by incongruent self-directed feedback, BOLD activity patterns also encompassed many of the regions mentioned above. However, recruitment of these regions was statistically weaker than in the partner-alone feedback contrasts, accompanied by lower affective responses and emotional similarity with the partner. Drawing on prior research, one interpretation of these findings is that incongruent self-directed feedback may interfere with spontaneous responses to partner-directed feedback, thereby resulting in attenuated empathic responses. Such incongruent conditions may impose greater cognitive and emotional demands, requiring participants to process two streams of information that evoke potentially conflicting affective states. This added complexity may further constrain the capacity for empathic engagement. For instance, immersing one arm into ice water tends to enhance self-focused attention^56^, which may inhibit affective sharing of others’ pain as individuals pay less attention to others’ feelings^13^. Likewise, self-relevant negative mood and stress can suppress empathic resonance for others’ pain^16^, and dampen the mentalizing process^17,18^. On the other hand, we observed activity in the DLPFC and right SMG, particularly when participants received negative self-directed feedback. These regions have previously been associated with cognitive control, emotion regulation^38,39^, and overcoming emotional egocentricity bias^21^. The recruitment of activity in these areas may reflect increased regulatory and attentional demands to direct attention, interpret or elaborate on emotional states when processing incongruent self-directed feedback. Furthermore, our results revealed that incongruent negative self-directed feedback primarily disrupted participants’ affective responses to partner-directed feedback. Moreover, higher emotional similarity with the partner in the S_NEG_P_POS_ condition was associated with lower self-reported difficulties in emotion regulation, particularly in goal-directed emotion regulation, impulse control, and access to adaptive regulation strategies. These preliminary findings are consistent with the notion, though speculative, that effective empathic engagement in this context may rely on individuals’ capacity to regulate their own emotional and behavioral responses, thereby allowing greater attunement to others’ experiences and feelings. This finding also aligns with prior evidence showing that regulating negative emotions is typically more challenging than regulating positive emotions^38^. Together, these findings suggest that the task manipulation effectively modulates demand for control, including emotion regulation, by varying the presence of incongruent self-directed feedback. Particularly, negative self-directed feedback may place higher cognitive and emotional demands on individuals’ responses to positive partner-directed feedback.

Our sample was relatively large for a brain imaging study, thus enabling us to explore sources of between-subject variability in BOLD activity during the incongruent-feedback situations. Results from both ROI and whole-brain analyses demonstrated that the scores on EC_IRI were associated with activity in brain regions commonly reported involved in affective sharing in empathy literature, especially the dACC^10,12,52^. These findings suggest that individual differences in empathic concern may contribute to between-subject variability in spontaneous responses in these conflicting self- versus partner-directed feedback situations. Furthermore, we also observed that, during the S_NEG_P_POS_-S_NEG_ contrast, greater self-reported difficulties in emotion regulation—particularly in awareness, goal-directed regulation, and access to adaptive strategies—were associated with lower activity in the DMPFC. This finding suggests that individual differences in self-reported emotion regulation tendencies are associated with variability in spontaneous responding to partner-directed feedback, when navigating incongruent feedback situations. In prior literature, the DMPFC has been linked to mentalizing^10,57^ and prosocial behaviors^2,3,9,10^. One possible interpretation is that individuals with greater difficulties representing internal affective states, regulating their own emotions, and maintaining goal-directed control under emotional conflict may show reduced engagement of higher-order integrative processes required for shifting between self- and other-oriented perspectives. This, in turn, may attenuate DMPFC recruitment and constrain the adaptive interpretation of partner-directed feedback. Additionally, DMPFC may also reflect emotion-regulatory control processes when managing affectively conflicting self-referential information^58,59^, suggesting a potential link to individual differences in emotion regulation difficulties. Converging with this neural finding, we also observed that when responding to positive feedback directed at their partner while simultaneously receiving negative feedback about themselves, individuals who showed stronger MFG engagement—a region involved in emotion regulation—tended to infer that their partner felt more positive affect following the partner’s positive feedback. Together, these findings align with prior research highlighting the link between self-reported emotion regulation and perspective-taking or empathy^60,61^. Therefore, we speculate that, when navigating incongruent feedback situations, individuals with stronger emotion regulation abilities may be more capable of managing their response tendencies to negative self-directed feedback, adopting their partner’s perspective, and showing affective resonance with their partner.

Of particular note, higher BOLD activity in the DMPFC was also associated with real-world supportive behavior, as reported by their partners in the daily diaries completed over the course of 7–10 days outside of the laboratory. This result is consistent with previous studies highlighting the role of MPFC during empathy in predicting prosocial behaviors^2,3,9,10^. Moreover, BOLD activity emerging in the S_NEG_P_POS_-S_NEG_P_XX_ contrast showed markedly stronger associations with daily support provided to the partner than the S_XX_P_POS_-S_XX_P_XX_ or S_XX_P_NEG_-S_XX_P_XX_ contrast (Supplementary Table 10). Together, these findings suggest that the brain activity in this more challenging and ecologically valid context may be informative for understanding individual variability in empathic behavior in daily life.

### Limitations

Although this study has several strengths—including a dyadic fMRI paradigm simulating everyday empathic contexts, a diverse and sizable sample, and partner-reported daily data—its findings should be interpreted in light of several limitations and opportunities for extension in future studies. First, although covariate analyses did not show evidence for a significant effect of participants’ belief in the cover story, future studies could adopt strategies to further enhance believability and minimize potential confounding processes, such as confusion or skepticism about the validity of the feedback received. Second, while the task aimed to elicit spontaneous empathic responses, the simultaneous feedback may have evoked other psychological states. Accordingly, our interpretation of the neuroimaging results is guided by the task design and observed behavioral patterns within an empathy framework, rather than assuming that each activated region reflects a single, process-specific function. We further note that these neural circuits, while commonly recruited during empathic engagement, are multifunctional and may also support broader social and cognitive processes^62^. For instance, engagement of the DMPFC may reflect not only mentalizing about the partner’s emotions, but also judgments about the rater’s intentions, or contempt toward someone rejecting one’s partner. Third, although we measured trial-by-trial variation in participants’ affective responses and post-experimental self-report measures, these can provide only indirect and potentially inaccurate accounts of participants’ experiences and therefore should be interpreted with appropriate caution. Future studies could include direct self-report measures capturing different facets of empathy and participants’ perceptions of the difficulty of expressing empathy to better understand and interpret the empathic processes engaged during the task. Fourth, as daily support ratings were collected one week prior to scanning, it is possible that neural responses were shaped by recent interpersonal experiences. This interpretive ambiguity underscores the need for tighter temporal alignment between behavioral and neural measures, and ideally, the use of causal or longitudinal designs to assess directionality. Fifth, whether these findings extend beyond intimate relationships in social contexts remains an important avenue for future work. Incorporating additional conditions, such as familiar or neutral others, as well as non-social conditions would allow future work to disentangle partner-specific effects from broader social-cognitive or more domain general executive processes. Future studies could also employ real-time reciprocal interactions to better illuminate how empathy unfolds in dyadic contexts. Finally, it is worth noting that consistently suppressing one’s own needs in favor of supporting others may have negative mental health consequences^63,64^, while meeting one’s own needs may enhance one’s capacity to support others. Future research should explore how individuals navigate this balance, to inform a more nuanced understanding of how to sustain both well-being and effective interpersonal support.

## Conclusions

In summary, this paper investigated spontaneous empathic responses during challenging social situations within romantic relationships and their associations with trait empathy, emotion regulation tendencies, and daily empathic behaviors. Using the CLOSE task, we found that responding to partner-directed feedback in conflicting social contexts, especially in the presence of incongruent negative self-related experience, was associated with regions within the frontoparietal areas (e.g., DLPFC, frontal pole, and SMG) beyond those typically observed in the empathy literature. When responding to positive partner-directed feedback in the presence of negative self-directed feedback, individual differences in self-reported difficulties in emotion regulation were related to BOLD activity in the DMPFC. These findings suggest that individual differences in self-reported emotion regulation tendencies may contribute to variability in empathic responding under incongruent feedback situations. Moreover, greater engagement of the DMPFC was associated with greater partner-rated daily supportive behaviors assessed outside the laboratory. Collectively, these findings shed light on within and between-person factors behind everyday empathy in close relationships, with implications for interventions targeting emotion regulation and empathic processes in romantic relationships. Future studies should further validate the present paradigm and replicate these findings.

## Supplementary information

Transparent Peer Review file

Supplementary Information

Reporting summary

## Data Availability

The minimal dataset necessary to interpret and replicate the findings reported in this article has been publicly deposited. The intermediate behavioral measures, ROI-extracted values, and whole-brain activity maps generated during this study have been deposited in the Open Science Framework and are publicly accessible at https://doi.org/10.17605/OSF.IO/63VNC^65^.

## Code availability

The analysis was conducted using a combination of publicly available toolboxes and custom code. The custom analysis code supporting the core findings of this study (utilizing R packages) has been deposited in the Open Science Framework and is publicly accessible at https://doi.org/10.17605/OSF.IO/63VNC^65^.
