# Supplementary material for: Neural responses to conflicting self- and partner-directed feedback correlate with trait emotion regulation and daily support
Source: Commun Psychol. 2026 Jul 2;4:147. doi: 10.1038/s44271-026-00493-w (PMC13690568; doi:10.1038/s44271-026-00493-w)
Supplement: Supplementary file 1 [file 44271_2026_493_MOESM1_ESM.pdf]

nature portfolio

Peer Review File

When Empathy Gets Tough: Neural Responses to Conflicting Self- and Partner-Directed Feedback Correlate with Trait Emotion Regulation and Daily Support

Corresponding Author: Dr Jessica Andrews-Hanna

This file contains all editorial decision letters in order by version, followed by all author rebuttals in order by version.

This manuscript has been previously reviewed at another Nature Portfolio journal. This document only contains reviewer comments and rebuttal letters for versions considered at Communications Psychology.

Version 0:

Decision Letter:

** Please ensure you delete the link to your author homepage in this e-mail if you wish to forward it to your coauthors **

Dear Dr Andrews-Hanna,

Thank you for submitting your manuscript titled "When Empathy Gets Tough: Neural Responses to Conflicting Self- and Partner-Directed Feedback in a Novel Paradigm" to Communications Psychology. We have given the paper our careful consideration and find it of potential interest. However, due to certain shortcomings we are concerned that sending the current manuscript out to review could lead to unnecessary delays and quite possibly an undesirable outcome of the review process.

In particular, please address the concerns raised by Reviewer 3 regarding the transparent reporting of all pertinent analyses (e.g., additional analyses run in response to their third comment). Additional exploratory analyses can be reported in the Supplement. Please note that differences in statistical significance across two tests are not themselves evidence for a statistically significant difference across contrasts; this must be demonstrated with appropriate statistics (see Gelman, A. & Stern, H. The difference between “significant” and “not significant” is not itself statistically significant. Am. Stat. 60, 328–331 (2006)).

Please also provide additional information regarding all measures collected (Reveiwer 3, Comment 2). This can be via a link to an OSF or similar project page.

Editorially, we strongly recommend the inclusion of behavioural work in the revision to satisfy the concerns raised by Reviewer 4 regarding lack of validation.

Please do not remove results from the paper, although exploratory results may be moved to the Supplement.

We would therefore like to invite you to revise your manuscript to address these concerns before we make a final determination on whether to send your manuscript for external review.

We shall hope to receive your revised version as soon as you are able to complete the suggested revisions. If something similar is published in the interim we will have to consider the impact it has on the novelty of a revised manuscript.

If you anticipate a delay of more than four weeks, please let us know. Should your manuscript be substantially delayed without notifying us in advance and your article is eventually published, the received date may be that of the revised, not the original, version.

We also ask that you ensure your manuscript complies with our editorial policies and reporting requirements.

If you are not interested in submitting a suitably revised manuscript in the future please let me know immediately so we can close your file. If you have any questions, please contact me.

Please use the link below when you are prepared to resubmit.

Link Redacted

Thank you for your interest in Communications Psychology.

Best regards,
Jennifer Bellingtier

Jennifer Bellingtier, PhD
Senior Editor
Communications Psychology

Version 1:

Decision Letter:

** Please ensure you delete the link to your author homepage in this e-mail if you wish to forward it to your coauthors **

Dear Dr Andrews-Hanna,

Your manuscript titled "When Empathy Gets Tough: Neural Responses to Conflicting Self- and Partner-Directed Feedback in a Novel Paradigm" has now been seen by our reviewers, whose comments appear below. In light of their advice I am delighted to say that we are happy, in principle, to publish a suitably revised version in Communications Psychology.

We therefore invite you to revise your paper one last time to address the remaining concerns of our reviewers and a list of editorial requests. Please note that we require revisions that address the referee's lingering concerns, i.e., more careful language, limitations and caveats not just in the discussion but also in the abstract. At the same time we ask that you edit your manuscript to comply with our format requirements and to maximise the accessibility and therefore the impact of your work.

EDITORIAL REQUESTS:

Please review our specific editorial comments and requests regarding your manuscript in the attached "Editorial Requests Table". Please outline your response to each request in the right hand column. Please upload the completed table with your manuscript files as a Related Manuscript file.

If you have any questions or concerns about any of our requests, please do not hesitate to contact me.

SUBMISSION INFORMATION:

In order to accept your paper, we require the files listed here https://www.nature.com/documents/commsj-file-checklist.pdf .

OPEN ACCESS:

Communications Psychology is a fully open access journal. Articles are made freely accessible on publication. For further information about article processing charges, open access funding, and advice and support from Nature Research, please visit https://www.nature.com/commspsychol/open-access

At acceptance, you will be provided with instructions for completing the open access licence agreement on behalf of all authors. This grants us the necessary permissions to publish your paper. Additionally, you will be asked to declare that all required third party permissions have been obtained, and to provide billing information in order to pay the article-processing charge (APC).

* TRANSPARENT PEER REVIEW: Communications Psychology uses a transparent peer review system. On author request, confidential information and data can be removed from the published reviewer reports and rebuttal letters prior to publication. If you are concerned about the release of confidential data, please let us know specifically what information you would like to have removed. Please note that we cannot incorporate redactions for any other reasons.

* CODE AVAILABILITY: All Communications Psychology manuscripts must include a section titled "Code Availability" at the end of the methods section. We require that the custom analysis code supporting your conclusions is made available in a publicly accessible repository at this stage; please choose a repository that generates a digital object identifier (DOI) for the code; the link to the repository and the DOI must be included in the Code Availability statement. Publication as Supplementary Information will not suffice.

* DATA AVAILABILITY:
All Communications Psychology manuscripts must include a section titled "Data Availability" at the end of the Methods section. More information on this policy, is available in the Editorial Requests Table and at <a

href="http://www.nature.com/authors/policies/data/data-availability-statements-data-citations.pdf">http://www.nature.com/authors/policies/data/data-availability-statements-data-citations.pdf.

Please use the following link to submit the above items:
Link Redacted
** This url links to your confidential home page and associated information about manuscripts you may have submitted or be reviewing for us. If you wish to forward this email to co-authors, please delete the link to your homepage first **

We hope to hear from you within two weeks; please let us know if you need more time.

Best regards,

Jennifer Bellingtier

Jennifer Bellingtier, PhD
Senior Editor
Communications Psychology

REVIEWERS' COMMENTS:

Reviewer #1 (Remarks to the Author):

The authors have been very responsive to the reviewer comments, so overall,
this paper seems "good to go"; what remains is some skepticism regarding the still very large number of analyses and analytic flexibility (in part a result of the extensive addressing of several rounds of reviewer comment), which - in the absence of preregistration - prompts me to add to title and abstract that this work should be regarded as preliminary and exploratory (I appreciate including the one sentence in the main text re: this aspect)

** Visit Nature Research's author and referees' website at www.nature.com/authors for information about policies, services and author benefits**

Open Access This Peer Review File is licensed under a Creative Commons Attribution 4.0 International License, which

permits use, sharing, adaptation, distribution and reproduction in any medium or format, as long as you give appropriate credit to the original author(s) and the source, provide a link to the Creative Commons license, and indicate if changes were made.
In cases where reviewers are anonymous, credit should be given to 'Anonymous Referee' and the source.
The images or other third party material in this Peer Review File are included in the article’s Creative Commons license, unless indicated otherwise in a credit line to the material. If material is not included in the article’s Creative Commons license and your intended use is not permitted by statutory regulation or exceeds the permitted use, you will need to obtain permission directly from the copyright holder.
To view a copy of this license, visit https://creativecommons.org/licenses/by/4.0/

Dear reviewers,

We sincerely thank the reviewers for their thorough and thoughtful feedback. We greatly appreciate the time and effort invested in evaluating our manuscript and for raising a number of important conceptual, methodological, and interpretive issues. These comments have been invaluable in helping us refine our framing and improve the clarity and interpretation of our findings.

In response, we have substantially revised the manuscript to enhance its framing and clarity, methodological rigor, and interpretability. Key revisions include: (1) clarifying the empirical scope of spontaneous responses to partner-related social experiences under conditions involving conflicting self-related information; (2) integrating multiple lines of converging evidence supporting spontaneous empathy processing in the CLOSE paradigm; (3) improving methodological transparency by reporting additional results and sharing a list of measures; (4) applying more stringent statistical corrections for exploratory whole-brain regression analyses; (5) adding analyses of DERS subscales and relocating dyadic analyses to the Supplementary Information to streamline the main text and improve focus; and (6) tempering interpretive claims in the Discussion, acknowledging limitations of the current design, and expanding directions for future research. All major changes made in direct response to the reviewers' comments have been highlighted in red in the revised manuscript.

We believe these revisions substantially strengthen the manuscript and address the key concerns raised during the review process. We respectfully submit the revised manuscript for consideration in *Communications Psychology*. Please see our point-by-point responses below.

Reviewers' comments:

Reviewer #3 (Remarks to the Author):

Comment 1: This appeal does a good job of bringing new evidence and interpretation to bear in addressing the concerns raised in prior reviews.

The new imaging analyses and affect rating similarity analyses are interesting and improve the paper significantly. In particular, one specific analysis provides the best evidence that empathy is at play: "As shown in Fig. 1C, self-reported affect in the SXXPPOS and SXXPNEG conditions positively correlates with post-scan inferred partner-affect in each corresponding condition, suggesting that such mentalizing contributed to their empathic emotional responses in the CLOSE task."

Response: We thank the reviewer for the positive feedback. We are glad that the new analyses effectively demonstrate the evidence of empathy and mentalizing in our task.

Comment 2: That said, before commenting in more detail on some aspects of changes, there needs to be discussion of the fact that the authors have introduced data from two new individual difference scales – scales that this reviewer mentioned in a prior review

that it would have been good for the paper to have used. In the prior review, I noted that these measures are in common usage in the literature, and could be useful for shedding light on what is happening in this study, but they appeared to have been left out and/or substituted for less common measures by the authors. The prior submission did not mention that two new scales that appear in this revision – the IRI and DERS – had been completed by participants. This revision doesn't provide much, if any, information about where these measures have come from or why they were not mentioned in the prior submission. To be sure, many studies include more questionnaire measures than end up being included in a given ms., but it is customary to at least state in the procedures that other measures were included – but are not discussed because they were not of interest in the present ms. The present ms. states briefly that 'demographic measures' were completed. Is this a reference to when the IRI and DERS were completed? It isn't clear as written.

The bottom line is that if new measures are introduced in a revision, the authors should explain why they were initially omitted, and how / when the data were collected. At present, the ms. simply describes the new analyses without any context, except for a passing mention that these measures, 'were also included.' But there is a larger issue here: Based on the way the information has been presented, it feels somewhat convenient that the authors of a ms. happen to have data from the two measures that a Reviewer mentions would have been good to have been included. It is not that I think authors are being intentionally deceptive, but there is a concern about completeness and transparency. I went back to the prior submissions to be sure that these measures were not mentioned – I didn't see evidence that they were. I also didn't see any clear description in the procedure – either in the prior revision or in this appeal - of exactly when any of the individual difference measures were completed. When were the individual difference measures (e.g. IRI, DERS and other measures mentioned in the paper) completed? Were all such measures collected at the same time or at some other time? Could the authors provide a list of what other measures were collected that these were a part of, assuming there was a larger battery of measures included? It would be desirable for the authors to provide more information here.

Response: We appreciate the reviewer's call for transparency. This study was part of a larger ongoing project in which we administered a broad battery of psychometric instruments to participants assessing sociodemographic characteristics, wellbeing, relationship health and social functioning. To clarify, the demographic measures were collected in Session 1, while the trait questionnaires (including the IRI and DERS, along with the EAI) were administered in Session 2, on the same day as the fMRI task. We have now updated the Methods section (p. 22) to provide a more detailed overview of the primary measurement domains and the key scales collected during Session 2. We also provide a full list of questionnaires collected in Session 1 and Session 2 via the OSF repository, where we have shared the relevant data and code for the current study. In our initial submission, we initially prioritized the EAI due to its conceptual alignment with our hypotheses and its inclusion of emotion regulation within empathy. However, following feedback from Reviewer 3, we switched analyses to the more widely used IRI and DERS to strengthen the methodological rigor and interpretability of the study. Importantly, results yielded similar patterns across the

different questionnaires used. For transparency, we now also report EAI-related findings in a footnote on p. 13 and in the Supplementary Information.

Comment 3: In the response letter the authors state that, "....to assess affective experience sharing, we computed emotional similarity with the partner under each feedback condition by comparing participants' self-reported affective ratings with their partner's self-reported affective ratings under the corresponding condition during scanning (Fig. 1D). Fig. 1E shows that individuals with higher trait empathic concern (measured by the score on empathic concern subscale of Interpersonal Reactivity Index (EC_IRI)) show higher alignment of emotional experiences with their partner (emotional similarity)." These data are interesting. The IRI has other subscales, however, as noted by the authors in other parts of the ms., e.g. when they report brain-behavior correlations with each separate subscale. For the behavioral similarity analyses, did the authors look for correlations with the other subscales (e.g. perspective taking) as well? Assuming the authors hypothesized that the EC subscale was the relevant one for this analysis, could the authors state their logic for why they expected the empathic concern subscale to show the reported effects and not the other subscales?

Response: We thank the reviewer for this insightful question. Our rationale for focusing on the EC_IRI is that the emotional similarity measure is intended to capture affective sharing with the partner. As such, it is conceptually closely aligned with the affective component of empathy, which is more indexed by EC, rather than the cognitive or mentalizing components assessed by PT_IRI. We have clarified this rationale result in the revised manuscript (p.9). We also conducted exploratory analyses examining the relationship between PT_IRI and emotional similarity. These analyses did not reveal significant associations, further supporting the specificity of the observed effect to the EC subscale. For transparency, we have also added this finding to the Results (p. 10).

Comment 4: It could be noted that the participant-partner affect rating correlations would logically be expected to be lower in the incongruent conditions than in the conditions where only the partner got feedback, given that the 'incongruent' conditions require participants to respond to two kinds of stimuli, with potentially mixed emotions, rather than one stimulus that might elicit less complex emotional states.

Response: We thank the reviewer for this helpful suggestion. We have now incorporated this interpretation into the Discussion section (p.16).

Comment 5: The paper reports that, "...participants' self-reported affective responses in the SXXPPOS or SXXPNEG condition during scanning were positively correlated with post-scan inferred partner-affect in the SXXPPOS or SXXPNEG condition. This finding suggests that participants' emotional reactions to partner-directed feedback were shaped by inferences about their partner's responses." The interpretation offered by the authors is plausible, as in the reverse possibility: one could use their own emotional reaction as basis for inferring other's reactions. This is often called projection and it was a focus of social neuroscience studies on person perception about 15 years ago, as well as in other behavioral literatures. Correlational similarity - by itself - doesn't provide

information about direction of causation. It could be acknowledged by both explanations are possible.

Response: We thank the reviewer for this helpful suggestion. We have now incorporated the alternative interpretation of “self-projection” into the Discussion section (p. 16).

Comment 6: On. P. 15, the discussion states, “These regions have been linked to cognitive control, emotion regulation42, 43, and overcoming emotional egocentricity bias21, suggesting that participants may have engaged regulatory processes to manage the emotions induced by incongruent self-directed feedback.” Perhaps this interpretation is correct. Alternatively, participants could simply be trying to make sense of a more emotionally complex situation and it is more cognitively demanding to figure out what one is feeling. This begs question as to what the term “regulation” means. If regulation means something narrow – an individual explicitly attempting to change their emotions – then I would again caution against that interpretation in the present data, as I do not believe they support such a conclusion. But if there is a more general interpretation at play – where cognitive control processes are deployed to direct attention to, make sense of, or interpret or elaborate on emotional states, then that interpretation is consistent with the present data.

Response: We thank the reviewer for this thoughtful and important clarification. We agree that our original interpretation may have been overly specific. As the reviewer suggests, we have adopted a more general interpretation, framing DLPFC and right SMG activity as reflecting broader cognitive control processes that could include a more specific capacity to regulate one’s emotions (p. 17).

Comment 7: The discussion continues.... “Furthermore, affective ratings revealed that incongruent negative—but not positive—self-directed feedback disrupted participants’ affective responses to partner-directed feedback. Moreover, emotional similarity with the partner in the SNEGPPOS condition was negatively associated with difficulties in emotion regulation, reinforcing the idea that successful empathic engagement under this condition required more effortful regulation.” Here, I would note that these data come from the overall score on the DERS, which includes multiple subscales, some of which concern awareness of and clarity of emotional states. See comment 4 above w/RE how to interpret what regulation means.

Response: We thank the reviewer for this important point that the total DERS score reflects multiple dimensions. To better address this issue, we have conducted follow-up analyses on the DERS-18 subscales. The result revealed that higher emotional similarity with the partner in the SNEGPPOS condition was specifically associated with lower difficulties in the Goal, Impulse, and Strategies subscales, rather than dimensions related to emotional Awareness, Clarity, and Nonacceptance subscales. These results help refine our interpretation by suggesting that effective empathic engagement in this conflicting context may be related to individual differences in goal-directed emotion regulation, impulse control, and access to adaptive regulation

strategies. We have incorporated these analyses into the Results (p. 11) and corresponding interpretations into the revised Discussion (p. 17). For consistency, the analyses relating DMPFC activity to DERS scores were updated in the same manner (p.13 and p. 18).

Comment 8: In this discussion that authors cite a Hamann paper in this way, “This interpretation aligns with prior evidence showing that regulating negative emotions is more challenging than regulating positive emotions, requiring greater recruitment of prefrontal cortex42.” The data in this paper can not be interpreted in terms of PFC activity reflecting something being more ‘challenging’ per se. Put another way, magnitude of PFC activity does not = effort or challenge. There can be greater or lesser PFC activity for various reasons in a given study.

Response: We thank the reviewer for this important clarification. We have revised the discussion and no longer refer to PFC activation in terms of “challenge,” to avoid such reverse inferences. Instead, our revised discussion focuses on the behavioral findings—namely that incongruent negative self-directed feedback reduced participants’ affective responses to positive partner-directed feedback, and that higher emotional similarity in the SNEGPPOS condition was associated with lower self-reported emotion regulation difficulties. We relate these findings to prior literature suggesting that negative emotions may place greater demands on emotion regulation capacity than positive emotions (p. 17).

Comment 9: On p. 16, the discussion states, “Among these associations, one particularly notable finding is that greater engagement of the DMPFC, during the SNEGPPOS-SNEGPXX contrast was related to higher emotion regulation.” This statement should be revised to reflect that fact that the authors observed a correlation with individual differences in self-reported emotion regulation tendencies. As written, the text could be interpreted as if the present paper directed measured emotion regulation ability. Ditto for this sentence on p. 18, “When responding to positive partner-directed feedback in the presence of negative self-directed feedback, activity in the prefrontal regions was linked to emotion regulation, suggesting that emotion regulation plays an important role in navigating incongruent feedback situations.” Instead of stating that PFC activity was linked to emotion regulation, the paper should say that is was linked to Individual differences in self-reported ER tendencies.

Response: The Reviewer raises a good point. We have revised the manuscript to consistently describe all results of the DERS as reflecting individual differences in self-reported emotion regulation difficulties or self-reported emotion regulation tendencies, rather than directly measured emotion regulation ability.

Comment 10: Finally, I would note again that this finding still seems ambiguous and unclear.... “when overall DMPFC activity was low, couples with higher inter-partner representational similarity reported lower relationship satisfaction, whereas when overall

DMPFC activity was high, couples with higher inter-partner representational similarity reported greater relationship satisfaction."

Response: We have tried to reword this section to increase clarity. More specifically, when couples' overall (mean) DMPFC activity was low, those with higher inter-partner representational similarity—indicating that both partners showed lower DMPFC activity—reported lower relationship satisfaction. In contrast, when couples' overall (mean) DMPFC activity was high, those with higher inter-partner representational similarity—indicating that both partners showed higher DMPFC activity—reported greater relationship satisfaction.

Our findings may suggest that similarity in how partners' respond neurally to the incongruent social experience is not always related to better relationship outcomes (Dryer & Horowitz, 1997; Kardum et al., 2018; Zhou et al., 2017), but rather depends on the overall level of neural activity. Specifically, high pattern similarity paired with low overall DMPFC activity may reflect a mutual disengagement or reduced mentalizing effort or empathic motivations, potentially indicating that both partners are less sensitive or attuned to each other's mental states. This may be particularly associated with lower relationships satisfaction. In contrast, high pattern similarity coupled with high overall activation may reflect active, reciprocal engagement in mentalizing or empathic motivations—suggesting not only alignment, but effortful, socially meaningful processing, which could support relationship quality.

We also moved the background, analyses, results, and discussions regarding examining the interaction between overall DMPFC activity, inter-partner representational similarity, and relationship satisfaction into the Supplementary Information (p. 52–56) to improve clarity and focus of the main manuscript, given that these results were overly complex.

Reviewer #4 (Remarks to the Author):

Comment 1: I read the paper and the reviews of Ma et al. with great interest (I was called in to mainly evaluate whether previous concern of another reviewer have been addressed).

In all brevity, my analysis of the work is mixed. Generally, the field of empathy research needs new approaches such as the one presented here; aspects such as investigating incongruency between feeling states and focusing on persons with grown relationships rather than more abstract "third anonymous person" approaches are steps into the right direction. The sample size for a neuroimaging study is impressive as well, and it is clear a lot of thought and effort has gone into this work, and I truly acknowledge that.

Response: We thank the reviewer for the careful evaluation of our manuscript and for recognizing the strengths of our work.

Comment 2: However, I am not convinced that the evidence presented in this paper fulfills the high standards of a journal such as Nature Comms. For one, I find it somewhat premature to use a new task without more extensive validation in a

neuroimaging study. Many aspects of how the task works, which cognitive-emotional processes it ties into, and how confounds or unspecific processes can be controlled for would need further and more specific investigation. I especially agree in this respect with the reviewer who dropped out. The authors did their best to address that reviewer's concerns using the data at hand, but I would consider those attempts as preliminary, post-hoc, and lacking proper control to be convincing. For instance, in the absence of anticipating concerns related to whether the task measures processes related to mentalizing, empathic concern or affective sharing/resonance, the authors need to rely on post-experimental interviews and dispositional self-report data. Such data suffer from numerous shortcomings, such as retrospective "rationalizations" or from the general shortcomings known for self-report and questionnaires.

Response: We thank the reviewer for this careful and critical comments. We agree that the current task does not yet provide exhaustive validation of all underlying cognitive and affective processes. While we agree that making claims based on results from a single variable may be too much of an overreach, in this case, multiple sources of behavioral and neuroimaging data converge to suggest that the CLOSE paradigm evokes spontaneous empathy and that control-related processes (e.g. emotion regulation) may play an important role (see Figure 1C and 1E). In addition, the new analyses for DERS subscales, suggested by Reviewer 3, found that participants' emotional similarity with their partner in the SNEGPPOS condition was negatively associated with the Goals, Impulse, and Strategies subscales (p. 11 and p. 17). These findings highlight the important role of emotion regulation in navigating incongruent interpersonal feedback, particularly in goal-directed regulation, impulse control, and access to adaptive regulatory strategies.

Moreover, we also now reference two recently published studies involving the CLOSE paradigm that further support the paradigm's validity (p. 22). One study involves a different subsample of the same project and focuses solely on behavioral findings to show that participants reporting higher positive relational interdependence were more likely to experience their partner's social feedback as a personal reward or loss (Guevara Beltran et al., 2026). The other study uses an independent fMRI dataset collected with the CLOSE paradigm and demonstrates that whole-brain predictive fMRI signatures of self-mentalizing and other-mentalizing—developed in the first part of the manuscript—show significant pattern expression during self-directed and partner-directed feedback conditions, respectively, compared to a no-feedback control condition (Açıl et al., 2025). Please note that although these studies employed the same paradigm, they used different subsamples and did not examine brain activity under incongruent feedback conditions.

In the revised manuscript, we have substantially toned-down mechanistic claims and more clearly delineate the empirical scope of what the task directly measures—namely spontaneous responses to a romantic partner's social experiences under incongruent feedback conditions, as well as their exploratory associations with daily empathic behaviors outside the laboratory (p. 6 and p. 8). We acknowledged that we didn't utilize explicit task instructions to empathize with one's partner, but this was a choice we deliberately made because we believe this design more closely reflects the complexity of empathic processes in everyday life. This paradigm is consistent with prior

research on empathy for pain, where participants are typically asked to observe others' emotional or sensory states and make judgments about both others' and their own feelings without extensive prescriptive guidance.

We have also refined the Discussion to provide more cautious interpretations, eliminate writing that could be perceived as overextended reverse inference, provide a more integrated synthesis of the converging findings, and more explicitly raise alternative explanations for the observed findings (p. 15–18). We have also acknowledged that simultaneous social feedback may elicit additional psychological processes and clarified that alternative functional interpretations of the observed neural activations are possible (p. 19). We have further clarified that post-experimental self-report measures provide only indirect and potentially biased accounts of participants' experiences and therefore should be interpreted with appropriate caution. We note that future studies could include direct self-report measures of participants' own empathy levels and their perceptions of the difficulty of expressing empathy (p. 19). Despite the issues raised and in light of our revisions, we believe that this study significantly advances our understanding of how empathy operates in real-world interpersonal contexts and highlights individual differences in empathic responding and supportive behavior.

Comment 3: Moreover, the sheer number of statistical analyses for subscales and added measures, especially when considering the absence of family-wise error corrections, render the conclusions the field can draw from the work very preliminary and in need of further validation.

Response: We appreciate the concern regarding multiple testing across subscale and additional behavioral measures. We now clarify which analyses were guided by specific hypotheses and which analyses were exploratory post-hoc analyses. Furthermore, we report FDR-corrected *p-values* for brain–behavior analyses and present more conservative thresholds for whole-brain regression analyses (p. 14).

Comment 4: Second, and this is a general concern that I do not think has been raised yet, I' d argue that the experimental design suffers from a lack control for two key aspects. First, it lacks a condition that allows to disentangle domain-general processes related to the (resolution or perception of) incongruency from processes specific for "social" (other-person oriented) incongruency processes. This is why previous work that the authors seem to base their task on (Silani et al.) has used both an "other" and a "self" condition (where everything is constant, but participants need to either report in their own or the other person's emotions) as well as congruent other and congruent self-conditions. Second, the design would benefit from a neutral or familiar other person as well. Otherwise, any claims related to the brain and cognitive processes being specifically connected to one's romantic partner remain premature and an assumption that may be plausible, but in need of validation. Finally, while the authors in their intro write to aim for higher ecological validity, and while I acknowledge their design takes some steps into the right direction, the task itself is still rather abstract and could possibly designed in a way that is more directly tied to "real" feedback (e.g. on task

performance, claims made or attitudes held, etc.); it could also be interesting to consider that couples will not always agree or have the same perspectives, and in some topics or issues may even have opposing views or a competitive conflict. In such cases, incongruency may have different implications as in those where views align.

Response: When designing the study, we considered adding a stranger-directed and a non-social feedback condition raised by the reviewer, but worried about the demands of the task as it already includes 9 conditions, including condition-specific control conditions. Instead, we reasoned that follow-up studies could be designed to address the questions raised by the reviewer. We outline these additional conditions as future directions in the discussion, and more ecologically valid interactive paradigms that may better capture how empathic processes unfold in real-time dyadic contexts (p. 20).

Comment 5: I regret my analysis is not more positive, and I am sorry they come in only after what must have been a rather lengthy and laborious review process; but I hope my comments are perceived as constructive to further improve the potential this line of work has for the field.

Response: We thank the reviewer for the thoughtful and constructive evaluation of our manuscript, and we appreciate the reviewer's helpful suggestions for improving this line of work. We hope that the revisions strengthen the manuscript and address the key concerns raised.

We thank the reviewers again for their time, thoughtful comments, and constructive feedback.

References

Açıl, D., Andrews-Hanna, J. R., Lopez-Sola, M., van Buuren, M., Krabbendam, L., Zhang, L., van der Meer, L., Fuentes-Claramonte, P., Pomarol-Clotet, E., Salvador, R., Debbané, M., Vrticka, P., Vuilleumier, P., Sbarra, D. A., Coppola, A. M., White, L. O., Wager, T. D., & Koban, L. (2025). Brain neuromarkers predict self- and other-related mentalizing across adult, clinical, and developmental samples. *bioRxiv*, 2025.03.10.642438. https://doi.org/10.1101/2025.03.10.642438

Dryer, D. C., & Horowitz, L. M. (1997). When do opposites attract? Interpersonal complementarity versus similarity. *Journal of Personality and Social Psychology*, *72*(3), 592–603. https://doi.org/10.1037/0022-3514.72.3.592

Guevara Beltran, D., Ma, S.-S., Maresh, E. L., Coppola, A. M., Mehl, M. R., Andrews-Hanna, J. R., & Sbarra, D. A. (2026). Interdependent Partners Experience Stronger Affective Responses to Simultaneous Positive and Negative Social Feedback. *Affective Science*. https://doi.org/10.1007/s42761-026-00353-0

Kardum, I., Hudek-Knezevic, J., Mehic, N., & Pilek, M. (2018). The effects of similarity in the dark triad traits on the relationship quality in dating couples. *Personality and Individual Differences*, *131*, 38–44. https://doi.org/10.1016/j.paid.2018.04.020

Zhou, Y., Wang, K., Chen, S., Zhang, J., & Zhou, M. (2017). The Actor, Partner, Similarity Effects of Personality, and Interactions with Gender and Relationship Duration among Chinese Emerging Adults. *Frontiers in Psychology*, *8*. https://doi.org/10.3389/fpsyg.2017.01698

Response to Reviewer #1

Comment: *The authors have been very responsive to the reviewer comments, so overall, this paper seems "good to go"; what remains is some skepticism regarding the still very large number of analyses and analytic flexibility (in part a result of the extensive addressing of several rounds of reviewer comment), which - in the absence of preregistration - prompts me to add to title and abstract that this work should be regarded as preliminary and exploratory (I appreciate including the one sentence in the main text re: this aspect).*

Response: We sincerely thank the reviewer for the positive evaluation of our manuscript and for recognizing our responsiveness throughout the revision process.

We appreciate the reviewer's concern regarding analytic flexibility and the absence of preregistration. As noted in the Introduction, although we had numerous *a priori* hypotheses, this fMRI study was not pre-registered. The reviewer also correctly points out that some of our analyses were inspired by prior reviews. Beyond the sentence we initially included in the Introduction "Our work was not preregistered, and we thus consider it exploratory and discovery-oriented despite our *a priori* hypotheses," we now temper language throughout the manuscript and more explicitly clarify the exploratory and preliminary nature of the present findings in the Abstract, Results, and Discussion sections. Edits made in response to the reviewer are highlighted in red (p. 3, p. 20, p. 22, p.24–27, and p. 30). Additional edits that the editor requested in line with copyright / formatting requirements are highlighted in the manuscript in blue font.

We have also revised the title to "When Empathy Gets Tough: Neural Responses to Conflicting Self- and Partner-Directed Feedback Correlate with Trait Emotion Regulation and Daily Support" to better reflect the central findings of the paper. While we fully appreciate the reviewer's rationale for suggesting that this framing also be incorporated into the title, we respectfully hope that the reviewer and Editor will agree that explicitly embedding terms such as "preliminary" and "exploratory" in the main title may unintentionally diminish the broader conceptual contribution and visibility of this study.

Again, we are deeply grateful for the reviewer’s exceptionally constructive feedback and guidance throughout the review process, which have substantially strengthened the rigor and clarity of the manuscript.
