# Supplementary material for: Neural responses to conflicting self- and partner-directed feedback correlate with trait emotion regulation and daily support
Source: Commun Psychol. 2026 Jul 2;4:147. doi: 10.1038/s44271-026-00493-w (PMC13690568; doi:10.1038/s44271-026-00493-w)
Supplement: Supplementary file 2 [file 44271_2026_493_MOESM2_ESM.pdf]

Supplementary Materials for

Neural Responses to Conflicting Self- and Partner-Directed Feedback Correlate with Trait Emotion Regulation and Daily Support

Shanshan Ma *et al.*

*Corresponding author: shanshanma@arizona.edu; sbarra@arizona.edu; jandrewshanna@arizona.edu

This PDF file includes:

Supplementary Figures 1 to 3
Supplementary Tables 1 to 10
Supplementary Notes 1 to 2

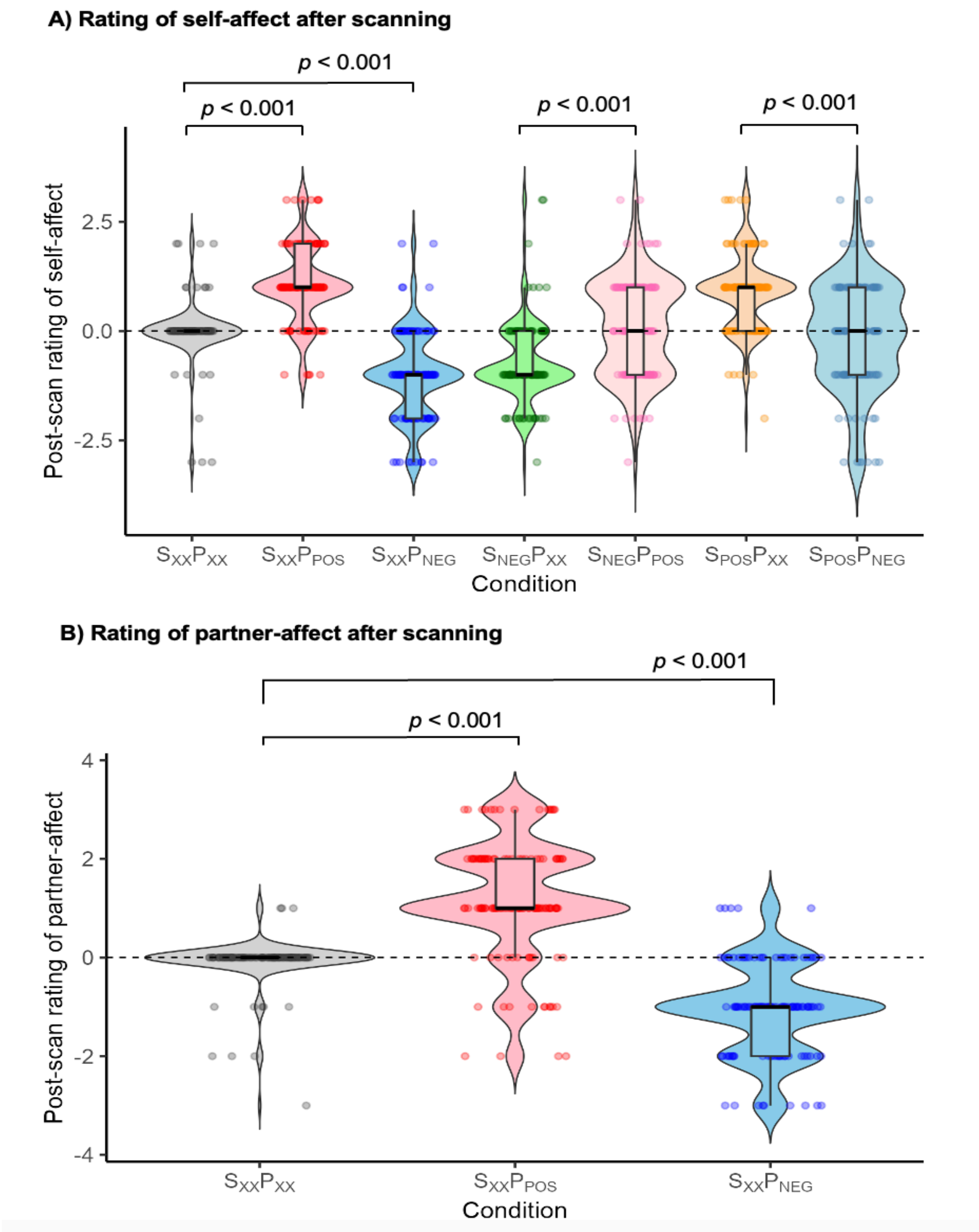

Supplementary Figure 1. Affective ratings completed retrospectively in questionnaires after scanning.

Participants (*N*=129) rated their emotions and speculated about their partner's emotions during each condition in the CLOSE task (from -3 (very negative) to 3 (very positive)).

(A) Ratings of the self-affect after scanning. Participants reported more positive or negative affective ratings when they received positive or negative partner-directed feedback alone, compared to when they received no feedback about their partner or themselves. Participants reported more positive or negative affective ratings when they received simultaneous incongruent feedback, compared to when they received self-directed feedback alone.

(B) Ratings of the partner affect after scanning. The post-task ratings of partner affect revealed that participants reported significantly higher or lower inferred partner affect in the $S_{XX}P_{POS}$ or $S_{XX}P_{NEG}$ condition than $S_{XX}P_{XX}$ condition.

Note that the *p* values displayed in the plot were derived via bootstrapping (5,000 iterations) due to deviation from model residual assumptions.

The broader shape of the violin plots illustrates the data distribution, with wider sections indicating higher density. The nested box plots display the median line (center line), quartiles (box edges representing the 25th and 75th percentiles), and whiskers extending to 1.5 x the interquartile range (IQR).

$S_{XX}P_{XX}$: Condition when participants received no feedback about their partner or themselves.

$S_{XX}P_{POS}$: Condition when participants received positive partner-directed feedback alone.

$S_{XX}P_{NEG}$: Condition when participants received negative partner-directed feedback alone.

$S_{NEG}P_{XX}$: Condition when participants received negative self-directed feedback alone.

$S_{NEG}P_{POS}$: Condition when participants received negative self-directed feedback and positive partner-directed feedback.

$S_{POS}P_{XX}$: Condition when participants received positive self-directed feedback alone.

$S_{POS}P_{NEG}$: Condition when participants received positive self-directed feedback and negative partner-directed feedback.

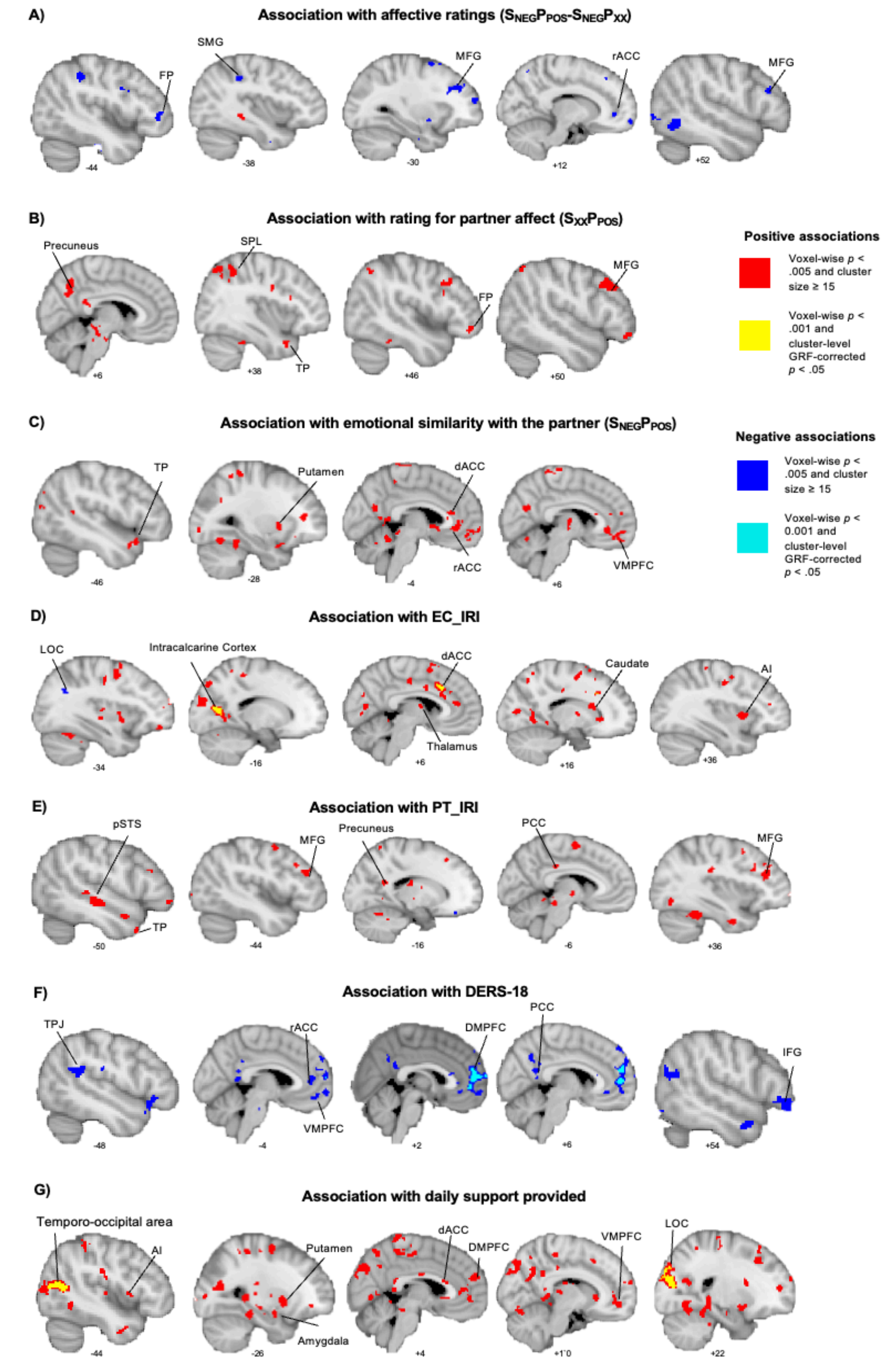

A)
Association with affective ratings (SNEGPPOS-SNEGPXX)
SMG
FP
MFG
rACC
MFG
-44
-38
-30
+12
+52
B)
Association with rating for partner affect (SXXPPOS)
Precuneus
SPL
MFG
FP
TP
+6
+38
+46
+50
Positive associations
Voxel-wise p < .005 and cluster size ≥ 15
Voxel-wise p < .001 and cluster-level GRF-corrected p < .05
C)
Association with emotional similarity with the partner (SNEGPPOS)
TP
Putamen
dACC
rACC
VMPFC
-46
-28
-4
+6
Negative associations
Voxel-wise p < .005 and cluster size ≥ 15
Voxel-wise p < 0.001 and cluster-level GRF-corrected p < .05
D)
Association with EC_IRI
LOC
Intracalcarine Cortex
dACC
Caudate
AI
Thalamus
-34
-16
+6
+16
+36
E)
Association with PT_IRI
pSTS
MFG
Precuneus
PCC
MFG
TP
-50
-44
-16
-6
+36
F)
Association with DERS-18
TPJ
rACC
DMPFC
PCC
IFG
VMPFC
-48
-4
+2
+6
+54
G)
Association with daily support provided
Temporo-occipital area
AI
Putamen
Amygdala
dACC
DMPFC
VMPFC
LOC
-44
-26
+4
+10
+22

Supplementary Figure 2. Results of whole-brain regression analysis in the $S_{NEG}P_{POS}$-$S_{NEG}P_{XX}$ contrast.

(A) BOLD activity negatively associated with affective responses in the $S_{NEG}P_{POS}$-$S_{NEG}P_{XX}$ contrast during scanning (*N* = 131).

(B) BOLD activity positively associated with post-scan inferred partner affect in the $S_{XX}P_{POS}$ condition (*N* = 129).

(C) BOLD activity positively associated with emotional similarity with the partner, operationalized as the negative value of the absolute difference between a participant's self-reported affective rating in the $S_{NEG}P_{POS}$ condition and their partner's self-reported affective rating in the $S_{POS}P_{XX}$ condition during scanning (*N* = 128).

(D) BOLD activity positively and negatively associated with scores on empathic concern subscale of the Interpersonal Reactivity Index (EC_IRI) (*N* = 131).

(E) BOLD activity positively associated with scores on perspective-taking subscale of the Interpersonal Reactivity Index (PT_IRI) (*N* = 131).

(F) BOLD activity negatively associated with scores on difficulties in emotion regulation scale (DERS-18) (*N* = 131).

(G) BOLD activity positively associated with partner-rated daily support provided to the partner (*N* = 131).

FP: frontal pole; SMG: Supramarginal Gyrus; MFG: middle frontal gyrus; rACC: rostral anterior cingulate cortex; SPL: superior parietal lobe; TP: temporal pole; dACC: dorsal anterior cingulate cortex; VMPFC: ventromedial prefrontal cortex; LOC: Lateral Occipital Cortex; AI: anterior insula; pSTS: posterior superior temporal sulcus; TPJ: temporoparietal junction; PCC: posterior cingulate cortex; DMPFC: dorsomedial prefrontal cortex; IFG: inferior frontal gyrus.

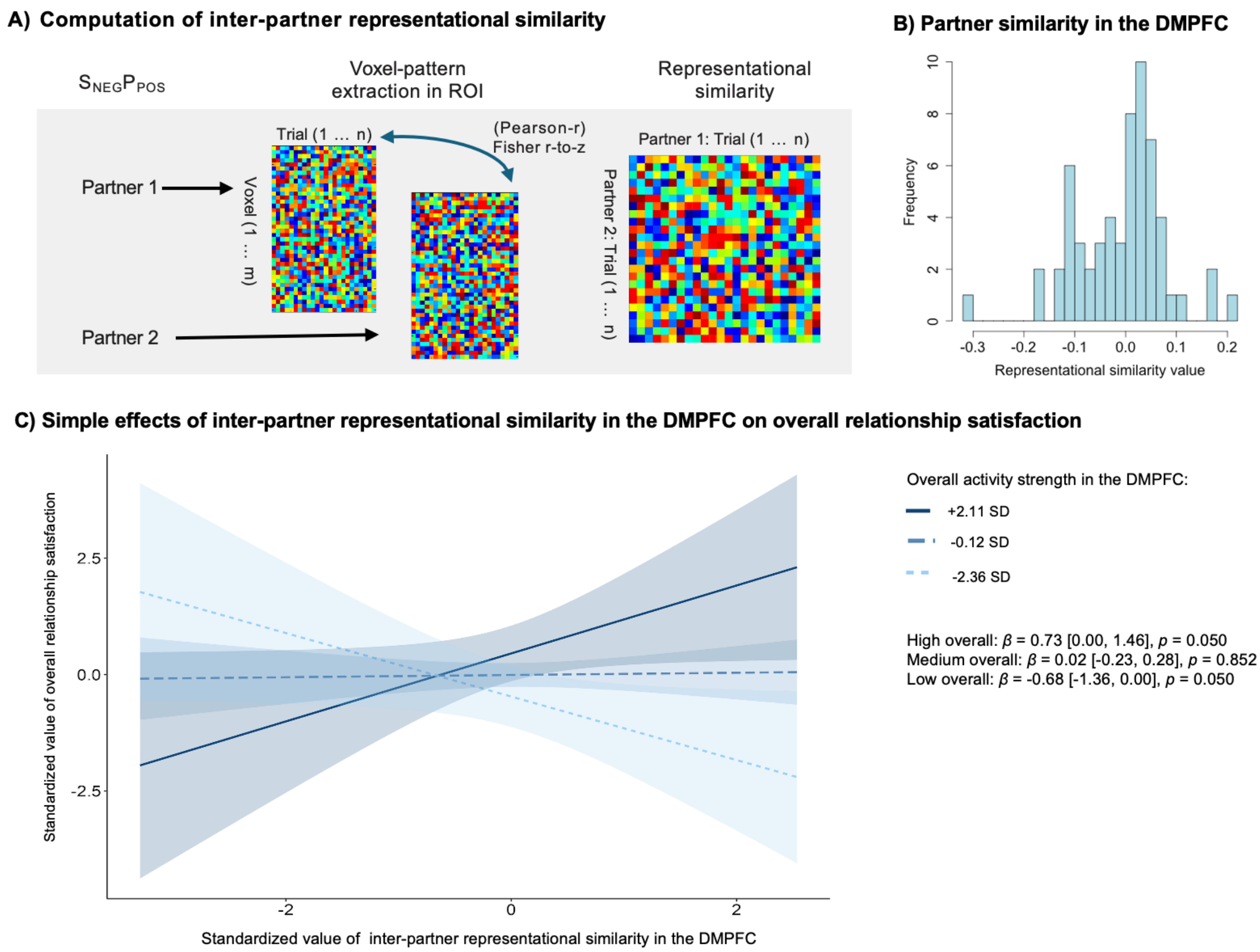

Supplementary Figure 3. The inter-partner representational similarity in the DMPFC during $S_{NEG}P_{POS}$ condition, and its association with overall relationship satisfaction.

(A) illustration of computation of representational similarity between partners.

(B) Distribution of inter-partner representational similarity in the DMPFC in the $S_{NEG}P_{POS}$ condition (N = 60 couples).

(C) Illustration of the simple effects of inter-partner representational similarity in the DMPFC in the $S_{NEG}P_{POS}$ condition on overall relationship satisfaction. The association between inter-partner representational similarity in the DMPFC and overall relationship satisfaction is significant when the overall activity strength in the DMPFC is either below -2.36 or above +2.11 standard deviations.

The overall activity strength and relationship satisfaction for each couple are calculated as the mean of the activity strength and self-reported relationship satisfaction from both partners.

Supplementary Table 1. Demographic characteristics of participants (N = 131)

	M (SD)
Age	31.8 (8.4)
Relationship duration	8.3 (6.3)
Cohabitating duration	6.5 (6.2)
Married (n)	76
Previously divorced (n)†	8
Number with children (n)	46
**Ethnicity**	
Not Hispanic or Latino	93
Hispanic or Latino	37
Not answered	1
**Race‡**	
White	106
American Indian or Alaska Native	2
Native Hawaiian or Other Pacific Islander	1
Asian	9
Black or African American	7
Other	9
Prefer not to disclose	5
**Gender**	
Woman	71
Man	51
Non-binary	6
Trans woman	0
Trans man	1
Other	1
Prefer not to disclose	1
**Sexual orientation**	
Bisexual	21
Gay/Lesbian	8
Heterosexual	89
Other	10
Prefer not to disclose	3
**Education**	
High school degree or equivalent	28
Bachelor's degree	54
Doctorate	10
Master's degree	27
Other	10
Prefer not to disclose	2

†: Two participants did not answer this question.
‡: Seven participants identified as more than one race.

Supplementary Table 2. Brain responses to partner-directed feedback alone

Contrast	Voxels	*Z value*	X	Y	Z	$p_{(cluster\text{-}GRF)}$	Brain region
$S_{XX}P_{POS}$> $S_{XX}P_{XX}$	62519	11.10	2	-64	48	< 0.001	Precuneus Cortex
		10.20	-4	10	56		Superior Frontal Gyrus
		9.65	-6	-52	28		Posterior Cingulate Gyrus
		9.32	-34	-2	62		Middle Frontal Gyrus
		9.30	-32	-2	54		Middle Frontal Gyrus
		9.24	-46	2	52		Precentral Gyrus
	1849	8.44	28	18	-18	< 0.001	Frontal Orbital Cortex
		6.29	64	-4	-22		Anterior Middle Temporal Gyrus
		5.83	32	28	2		Frontal Orbital Cortex
		5.79	46	16	-38		Temporal Pole
		5.27	56	4	-30		Anterior Middle Temporal Gyrus
		4.74	34	24	-30		Temporal Pole
	254	5.01	30	50	10	< 0.001	Frontal Pole
		3.82	36	48	18		Frontal Pole
	239	6.14	-2	-18	34	< 0.001	Posterior Cingulate Gyrus
		4.25	-4	-30	24		Posterior Cingulate Gyrus
	132	5.04	-50	-24	18	0.014	Parietal Operculum Cortex
		3.22	-62	-14	18		Postcentral Gyrus
$S_{XX}P_{POS}$< $S_{XX}P_{XX}$	388	5.39	52	44	0	< 0.001	Frontal Pole
		4.90	38	52	-14		Frontal Pole
		4.74	44	56	-12		Frontal Pole
		3.54	48	48	-12		Frontal Pole
	277	5.13	66	-28	44	< 0.001	Anterior Supramarginal Gyrus
		4.41	68	-36	38		Posterior Supramarginal Gyrus
		4.19	64	-28	28		Anterior Supramarginal Gyrus
	126	5.74	-32	-40	-10	0.017	Posterior Parahippocampal Gyrus
	117	4.63	-38	44	-14	0.024	Frontal Pole
		3.88	-36	52	-14		Frontal Pole
$S_{XX}P_{NEG}$> $S_{XX}P_{XX}$	20691	11.20	0	-64	42	< 0.001	Precuneus Cortex
		10.40	-6	-58	42		Precuneus Cortex
		10.20	-28	16	-18		Frontal Orbital Cortex
		9.59	-38	2	58		Middle Frontal Gyrus
		9.47	-34	2	58		Middle Frontal Gyrus
		8.96	-4	-50	28		Posterior Cingulate Gyrus
	18931	8.77	30	18	-16	< 0.001	Frontal Orbital Cortex
		8.03	-46	-78	-12		Inferior Lateral Occipital Cortex
		7.93	-38	-56	-20		Temporal Occipital Fusiform Cortex
		7.81	-40	-78	-16		Inferior Lateral Occipital Cortex
		7.62	-4	-28	-2		Brain-Stem
		7.37	-38	-88	-12		Inferior Lateral Occipital Cortex

	9526	9.57	-2	14	58	< 0.001	Superior Frontal Gyrus
		9.56	-4	14	54		Superior Frontal Gyrus
		8.92	-6	58	30		Frontal Pole
		8.63	-2	66	-4		Frontal Pole
		8.45	-4	58	26		Frontal Pole
		8.16	4	58	22		Frontal Pole
	557	4.87	4	-18	68	< 0.001	Precentral Gyrus
		4.58	2	-16	60		Precentral Gyrus
		4.31	-10	-34	64		Precentral Gyrus
		4.23	-4	-24	56		Precentral Gyrus
		3.99	-2	-18	54		Precentral Gyrus
		3.83	4	-24	60		Precentral Gyrus
	134	5.61	-2	-18	36	0.011	Posterior Cingulate Gyrus
	119	4.64	50	-28	44	0.019	Anterior Supramarginal Gyrus
		3.57	48	-20	40		Postcentral Gyrus
	113	4.66	32	54	10	0.024	Frontal Pole
$S_{XX}P_{NEG}$ < $S_{XX}P_{XX}$	720	6.25	62	-36	50	< 0.001	Posterior Supramarginal Gyrus
		5.68	62	-44	46		Posterior Supramarginal Gyrus
		5.43	62	-40	42		Posterior Supramarginal Gyrus
		4.54	62	-26	34		Anterior Supramarginal Gyrus
		4.49	62	-26	28		Anterior Supramarginal Gyrus
		4.20	52	-34	34		Parietal Operculum Cortex
	448	6.12	40	52	-14	< 0.001	Frontal Pole
		5.15	48	46	-6		Frontal Pole
		4.60	50	46	2		Frontal Pole
	275	4.47	-62	-34	44	< 0.001	Anterior Supramarginal Gyrus
		4.46	-62	-28	34		Anterior Supramarginal Gyrus
		4.34	-66	-28	40		Anterior Supramarginal Gyrus
		4.27	-62	-32	48		Anterior Supramarginal Gyrus
		3.80	-60	-34	52		Anterior Supramarginal Gyrus
		3.16	-48	-34	30		Anterior Supramarginal Gyrus
	267	5.01	8	-36	44	< 0.001	Posterior Cingulate Gyrus
		4.75	8	-28	42		Posterior Cingulate Gyrus
	250	5.66	-60	-58	-10	< 0.001	Middle Temporal Gyrus
	208	4.68	-40	-2	6	0.001	Insular Cortex
		4.04	-42	-10	-4		Insular Cortex
		3.97	-44	-6	-4		Insular Cortex
		3.84	-46	-10	0		Planum Polare
		3.53	-54	-2	0		Planum Polare
		3.53	-60	0	-2		Anterior Superior Temporal Gyrus
	146	5.12	-32	-38	-12	0.007	Posterior Parahippocampal Gyrus
		3.93	-28	-30	-20		Posterior Parahippocampal Gyrus
	137	4.83	-36	46	-14	0.010	Frontal Pole
		4.10	-36	40	-10		Frontal Pole

	3.73	-32	40	-8		Frontal Pole
108	4.65	54	-18	-26	0.030	Posterior Inferior Temporal Gyrus
	4.45	54	-18	-22		Posterior Middle Temporal Gyrus
	3.98	58	-28	-22		Posterior Inferior Temporal Gyrus
	3.83	58	-24	-20		Posterior Inferior Temporal Gyrus

Supplementary Table 3. Brain responses to partner-directed feedback in the presence of simultaneous incongruent self-directed feedback after regressing the belief in cover story (*N* = 129)

Contrast	Voxels	*Z value*	X	Y	Z	*p* (cluster-GRF)	Brain region
$S_{NEG}P_{POS}>S_{NEG}P_{XX}$	27853	7.38	-4	-58	50	< 0.001	Precuneus Cortex
		7.37	-4	-60	54		Precuneus Cortex
		7.16	-2	-70	58		Precuneus Cortex
		6.90	-2	-66	48		Precuneus Cortex
		6.56	6	-70	58		Precuneus Cortex
		6.44	10	-72	-6		Lingual Gyrus
	2717	5.58	36	14	-12	< 0.001	Insular Cortex
		5.33	38	18	-6		Insular Cortex
		5.24	10	12	6		Right Caudate
		5.19	-18	20	-2		Left Caudate
		5.13	42	20	-4		Frontal Orbital Cortex
		5.09	48	20	0		Frontal Operculum Cortex
	462	4.63	-10	-24	-4	< 0.001	Left Thalamus
		4.37	-14	-20	14		Left Thalamus
		4.30	14	-26	0		Right Thalamus
		4.27	-14	-32	8		Left Thalamus
		4.23	8	-22	10		Right Thalamus
		4.21	4	-22	0		Right Thalamus
	380	4.35	-30	64	12	< 0.001	Frontal Pole
		4.35	-28	58	8		Frontal Pole
		4.30	-26	54	14		Frontal Pole
		4.22	-22	64	2		Frontal Pole
	319	4.73	-60	-8	-16	< 0.001	Anterior Middle Temporal Gyrus
		4.48	-64	-12	-20		Posterior Middle Temporal Gyrus
		4.17	-62	-2	-22		Anterior Middle Temporal Gyrus
		3.98	-66	-10	-8		Anterior Middle Temporal Gyrus
		3.68	-66	-16	-8		Posterior Middle Temporal Gyrus
		3.66	-64	-12	-26		Posterior Middle Temporal Gyrus
	131	4.19	-50	44	-12	0.013	Frontal Pole
		3.88	-46	46	-4		Frontal Pole
		3.87	-40	42	-8		Frontal Pole
		3.76	-36	46	-2		Frontal Pole
	108	4.28	6	62	2	0.032	Frontal Pole
		4.16	10	64	4		Frontal Pole
		3.90	4	68	2		Frontal Pole
	107	4.11	38	-20	40	0.033	Postcentral Gyrus
		3.84	44	-18	40		Postcentral Gyrus
		3.71	52	-14	38		Postcentral Gyrus
		3.66	46	-10	30		Postcentral Gyrus

		3.51	58	-10	40		Postcentral Gyrus
SNEGPPOS<SNEGPXX	260	4.85	40	-42	-18	< 0.001	Temporal Occipital Fusiform Cortex
		4.46	46	-48	-22		Temporal Occipital Fusiform Cortex
		4.28	46	-44	-22		Temporal Occipital Fusiform Cortex
		4.21	40	-58	-16		Temporal Occipital Fusiform Cortex
		4.20	46	-56	-16		Temporal Occipital Fusiform Cortex
	192	5.85	52	44	8	0.002	Frontal Pole
		4.51	52	36	-2		Frontal Pole
		4.00	46	36	8		Frontal Pole
SPOSPNEG>SPOSPXX	3391	6.14	-52	20	2	< 0.001	Inferior Frontal Gyrus
		5.86	-40	20	30		Middle Frontal Gyrus
		5.86	-48	20	10		Inferior Frontal Gyrus
		5.78	-46	46	-8		Frontal Pole
		5.74	-50	20	36		Middle Frontal Gyrus
		5.69	-32	2	54		Middle Frontal Gyrus
	2948	6.90	-4	14	54	< 0.001	Superior Frontal Gyrus
		6.29	-6	38	56		Superior Frontal Gyrus
		5.59	-8	14	66		Superior Frontal Gyrus
		5.30	-4	28	40		Paracingulate Gyrus
		5.26	-8	22	42		Paracingulate Gyrus
		5.24	-2	56	28		Superior Frontal Gyrus
	918	5.24	-6	-62	46	< 0.001	Precuneus Cortex
		5.11	4	-66	50		Precuneus Cortex
		4.32	4	-60	36		Precuneus Cortex
		3.77	-10	-54	34		Precuneus Cortex
	757	5.11	56	-58	38	< 0.001	Angular Gyrus
		4.83	38	-48	42		Superior Parietal Lobule
		4.75	44	-58	44		Angular Gyrus
		4.52	46	-58	32		Angular Gyrus
		3.72	52	-64	46		Superior Lateral Occipital Cortex
		3.63	32	-42	36		Superior Parietal Lobule
	626	6.68	34	24	-12	< 0.001	Frontal Orbital Cortex
		4.84	32	28	0		Frontal Orbital Cortex
		4.59	46	20	8		Inferior Frontal Gyrus
		4.58	46	22	-2		Frontal Operculum Cortex
		3.94	50	38	-10		Frontal Pole
		3.58	56	28	-6		Inferior Frontal Gyrus
	480	4.67	-42	-54	48	< 0.001	Angular Gyrus
		4.63	-40	-50	48		Superior Parietal Lobule
		4.52	-34	-60	44		Superior Lateral Occipital Cortex
		3.80	-34	-46	40		Superior Parietal Lobule
		3.63	-40	-62	58		Superior Lateral Occipital Cortex

	303	5.57	54	10	-30	< 0.001	Temporal Pole
		4.61	58	0	-30		Anterior Middle Temporal Gyrus
	244	4.32	-54	-58	30	< 0.001	Angular Gyrus
		4.24	-46	-58	34		Angular Gyrus
		3.82	-56	-60	42		Superior Lateral Occipital Cortex
		3.79	-52	-60	50		Superior Lateral Occipital Cortex
		3.73	-50	-58	54		Angular Gyrus
		3.17	-64	-54	26		Angular Gyrus
	236	6.05	56	-32	-4	0.001	Posterior Middle Temporal Gyrus
		3.53	50	-22	-12		Posterior Middle Temporal Gyrus
	236	4.69	-58	-34	0	0.001	Posterior Superior Temporal Gyrus
		4.66	-50	-30	-4		Posterior Middle Temporal Gyrus
		3.47	-48	-38	-4		Posterior Middle Temporal Gyrus
	214	4.74	48	30	38	0.001	Middle Frontal Gyrus
		3.98	52	22	40		Middle Frontal Gyrus
		3.70	52	34	28		Middle Frontal Gyrus
	127	4.36	-48	2	-36	0.019	Temporal Pole
		4.27	-44	2	-38		Temporal Pole
		3.74	-50	6	-30		Temporal Pole
		3.33	-44	6	-32		Temporal Pole
$S_{POS}P_{NEG}<S_{POS}P_{XX}$	3985	6.33	30	-38	-18	< 0.001	Posterior Temporal Fusiform Cortex
		6.31	30	-58	-8		Temporal Occipital Fusiform Cortex
		6.19	50	-44	-18		Inferior Temporal Gyrus
		5.88	38	-86	16		Superior Lateral Occipital Cortex
		5.77	26	-66	-12		Occipital Fusiform Gyrus
		5.69	30	-44	-10		Temporal Occipital Fusiform Cortex
	2685	6.30	-48	-60	0	< 0.001	Middle Temporal Gyrus
		6.12	-32	-34	-16		Posterior Parahippocampal Gyrus
		5.56	-56	-66	-4		Inferior Lateral Occipital Cortex
		5.32	-28	-46	-20		Temporal Occipital Fusiform Cortex
		5.27	-46	-66	-8		Inferior Lateral Occipital Cortex
		5.27	-34	-46	-26		Temporal Occipital Fusiform Cortex
	1425	5.99	-8	-34	44	< 0.001	Posterior Cingulate Gyrus
		5.89	-12	-36	40		Posterior Cingulate Gyrus
		5.27	6	-36	40		Posterior Cingulate Gyrus
		5.15	8	-38	52		Precuneus Cortex
		4.91	16	-36	42		Precuneus Cortex
		4.75	10	-32	44		Posterior Cingulate Gyrus
	1097	5.59	62	-22	22	< 0.001	Anterior Supramarginal Gyrus
		5.44	64	-22	28		Anterior Supramarginal Gyrus
		5.12	64	-28	46		Anterior Supramarginal Gyrus

	4.95	62	-30	40		Anterior Supramarginal Gyrus
	4.62	64	-18	46		Postcentral Gyrus
	4.44	66	-14	8		Planum Temporale
1067	6.01	-34	-88	16	< 0.001	Superior Lateral Occipital Cortex
	4.92	-26	-88	16		Superior Lateral Occipital Cortex
	4.81	-34	-90	4		Inferior Lateral Occipital Cortex
	4.53	-44	-78	24		Superior Lateral Occipital Cortex
	4.23	-38	-82	26		Superior Lateral Occipital Cortex
	4.20	-20	-74	44		Superior Lateral Occipital Cortex
965	6.43	-26	36	-14	< 0.001	Frontal Orbital Cortex
	5.86	22	36	-14		Frontal Pole
	5.39	-32	38	-6		Frontal Pole
	4.99	2	44	-12		Frontal Medial Cortex
	4.77	32	36	-18		Frontal Pole
	4.69	10	36	-14		Frontal Medial Cortex
874	6.11	-8	-58	12	< 0.001	Precuneus Cortex
	5.17	-2	-56	8		Precuneus Cortex
	4.91	10	-52	12		Precuneus Cortex
	4.90	8	-58	14		Precuneus Cortex
	4.45	6	-56	4		Lingual Gyrus
	4.41	-4	-46	6		Posterior Cingulate Gyrus
796	5.58	-66	-30	30	< 0.001	Anterior Supramarginal Gyrus
	5.46	-60	-30	22		Parietal Operculum Cortex
	4.93	-58	-16	16		Central Opercular Cortex
	4.37	-66	-28	40		Anterior Supramarginal Gyrus
	4.31	-68	-22	30		Anterior Supramarginal Gyrus
	4.19	-66	-24	38		Anterior Supramarginal Gyrus
669	4.75	-42	2	6	< 0.001	Central Opercular Cortex
	4.74	-36	2	10		Insular Cortex
	4.64	-38	-14	0		Insular Cortex
	4.33	-38	-6	-4		Insular Cortex
	4.22	-40	-16	-4		Insular Cortex
	4.19	-26	-2	4		Left Putamen
549	6.74	2	4	38	< 0.001	Anterior Cingulate Gyrus
	4.19	0	14	32		Anterior Cingulate Gyrus
	3.61	8	2	48		Supplementary Motor Cortex
470	5.04	-10	-54	68	< 0.001	Superior Parietal Lobule
	4.16	-12	-52	60		Precuneus Cortex
	3.99	-26	-42	68		Superior Parietal Lobule
	3.98	-20	-46	56		Superior Parietal Lobule
	3.91	-20	-42	70		Postcentral Gyrus
	3.66	-22	-52	74		Superior Parietal Lobule
175	4.18	28	-20	-12	0.004	Right Hippocampus
	4.12	20	-20	-14		Posterior Parahippocampal Gyrus

	4.10	42	-12	-28		Posterior Temporal Fusiform Cortex
	3.93	34	-14	-20		Anterior Parahippocampal Gyrus
	3.90	32	-18	-14		Right Hippocampus
	3.54	36	-8	-38		Anterior Temporal Fusiform Cortex
174	4.62	32	-42	74	0.004	Superior Parietal Lobule
	4.32	50	-28	62		Postcentral Gyrus
	4.09	30	-46	66		Superior Parietal Lobule
	3.86	28	-38	62		Postcentral Gyrus
	3.53	40	-32	66		Postcentral Gyrus
	3.53	30	-48	58		Superior Parietal Lobule
173	4.99	38	2	4	0.004	Insular Cortex
	4.02	34	10	10		Insular Cortex
162	4.50	-46	-28	64	0.006	Postcentral Gyrus
	4.00	-38	-24	68		Postcentral Gyrus
	3.53	-32	-26	74		Postcentral Gyrus
	3.52	-32	-26	58		Precentral Gyrus
	3.41	-38	-26	60		Postcentral Gyrus
144	5.03	52	40	12	0.010	Frontal Pole
	4.89	48	40	10		Frontal Pole
141	4.33	36	-16	4	0.012	Insular Cortex
	4.12	40	-8	-4		Insular Cortex
	3.66	30	-16	-2		Right Putamen
	3.44	42	2	-20		Planum Polare
	3.34	38	-6	-12		Insular Cortex
126	4.70	18	-54	66	0.020	Superior Parietal Lobule
	3.94	18	-46	64		Superior Parietal Lobule
107	4.64	22	-74	46	0.040	Superior Lateral Occipital Cortex
	3.76	26	-72	38		Superior Lateral Occipital Cortex

Supplementary Table 4. Brain responses to partner-directed feedback in the presence of simultaneous incongruent self-directed feedback after regressing age and relationship duration

Contrast	Voxels	*Z value*	X	Y	Z	*p* (cluster-GRF)	Brain region
$S_{NEG}P_{POS}$>$S_{NEG}P_{XX}$	26564	7.45	-4	-58	50	< 0.001	Precuneus Cortex
		7.39	-4	-60	54		Precuneus Cortex
		7.23	-2	-62	58		Precuneus Cortex
		7.15	-2	-70	58		Precuneus Cortex
		6.56	2	14	54		Superior Frontal Gyrus
		6.55	6	-70	58		Precuneus Cortex
	2826	5.87	36	14	-12	< 0.001	Insular Cortex
		5.38	38	18	-6		Insular Cortex
		5.24	10	12	6		Right Caudate
		5.23	50	20	0		Inferior Frontal Gyrus
		5.23	42	20	-4		Frontal Orbital Cortex
		5.08	-18	20	-2		Left Caudate
	2488	5.60	42	-44	42	< 0.001	Posterior Supramarginal Gyrus
		5.09	62	-58	28		Angular Gyrus
		5.06	56	-60	30		Superior Lateral Occipital Cortex
		4.99	50	-38	46		Posterior Supramarginal Gyrus
		4.98	52	-58	28		Angular Gyrus
		4.95	48	-44	60		Posterior Supramarginal Gyrus
	416	4.61	-28	58	8	< 0.001	Frontal Pole
		4.53	-30	64	12		Frontal Pole
		4.53	-26	54	14		Frontal Pole
		4.18	-22	64	2		Frontal Pole
	416	4.70	-10	-24	-4	< 0.001	Left Thalamus
		4.31	-14	-20	14		Left Thalamus
		4.29	4	-22	0		Right Thalamus
		4.18	-14	-32	8		Left Thalamus
		4.17	-22	-28	0		Left Cerebral White Matter
		4.05	12	-18	14		Right Thalamus
	299	4.59	-60	-8	-16	< 0.001	Anterior Middle Temporal Gyrus
		4.30	-68	-16	-16		Posterior Middle Temporal Gyrus
		3.94	-62	-2	-22		Anterior Middle Temporal Gyrus
		3.89	-66	-10	-8		Anterior Middle Temporal Gyrus
		3.52	-64	-12	-26		Posterior Middle Temporal Gyrus
		3.49	-48	-12	-16		Posterior Middle Temporal Gyrus
	158	4.63	2	40	0	0.005	Anterior Cingulate Gyrus
		3.99	-2	30	12		Anterior Cingulate Gyrus
		3.86	4	34	10		Anterior Cingulate Gyrus
	115	4.24	-48	48	-12	0.024	Frontal Pole

		3.97	-40	42	-8		Frontal Pole
		3.68	-46	46	-4		Frontal Pole
		3.50	-36	46	-2		Frontal Pole
$S_{NEG}P_{POS}<S_{NEG}P_{XX}$	223	4.62	40	-42	-18	0.001	Temporal Occipital Fusiform Cortex
		4.22	46	-48	-22		Temporal Occipital Fusiform Cortex
		4.19	46	-56	-16		Temporal Occipital Fusiform Cortex
		4.13	40	-58	-16		Temporal Occipital Fusiform Cortex
		4.09	46	-44	-22		Temporal Occipital Fusiform Cortex
	157	5.23	52	44	8	0.005	Frontal Pole
		3.85	54	36	18		Frontal Pole
		3.73	52	36	-2		Frontal Pole
		3.70	46	36	8		Frontal Pole
$S_{POS}P_{NEG}>S_{POS}P_{XX}$	3331	6.01	-52	20	2	< 0.001	Inferior Frontal Gyrus
		5.93	-50	20	36		Middle Frontal Gyrus
		5.86	-40	20	30		Middle Frontal Gyrus
		5.85	-30	20	-12		Frontal Orbital Cortex
		5.81	-46	46	-8		Frontal Pole
		5.61	-28	28	0		Frontal Orbital Cortex
	3029	6.84	-2	14	52	< 0.001	Paracingulate Gyrus
		6.52	-8	36	58		Superior Frontal Gyrus
		5.54	-4	28	40		Paracingulate Gyrus
		5.54	-6	48	50		Frontal Pole
		5.44	-2	56	28		Superior Frontal Gyrus
		5.24	-8	22	42		Paracingulate Gyrus
	1011	5.31	-6	-62	46	< 0.001	Precuneus Cortex
		5.23	2	-66	48		Precuneus Cortex
		4.84	10	-62	50		Precuneus Cortex
		4.60	4	-60	36		Precuneus Cortex
		3.94	-12	-54	30		Posterior Cingulate Gyrus
		3.86	-10	-50	28		Posterior Cingulate Gyrus
	849	5.47	54	-56	38	< 0.001	Angular Gyrus
		4.89	44	-58	44		Angular Gyrus
		4.88	38	-48	42		Superior Parietal Lobule
		4.71	46	-58	32		Angular Gyrus
		3.94	48	-44	48		Posterior Supramarginal Gyrus
		3.93	52	-64	46		Superior Lateral Occipital Cortex
	592	6.89	34	24	-12	< 0.001	Frontal Orbital Cortex
		4.87	32	28	0		Frontal Orbital Cortex
		4.71	46	20	8		Inferior Frontal Gyrus
		4.70	44	20	0		Frontal Operculum Cortex
		3.68	56	28	-6		Inferior Frontal Gyrus
	542	4.80	-40	-52	48	< 0.001	Angular Gyrus
		4.64	-34	-60	44		Superior Lateral Occipital Cortex
		3.98	-34	-46	40		Superior Parietal Lobule
		3.67	-40	-62	58		Superior Lateral Occipital Cortex

		3.46	-48	-40	44		Anterior Supramarginal Gyrus
		3.46	-44	-40	42		Anterior Supramarginal Gyrus
	297	5.44	54	8	-32	< 0.001	Temporal Pole
		4.43	58	0	-30		Anterior Middle Temporal Gyrus
	263	6.24	56	-32	-4	< 0.001	Posterior Middle Temporal Gyrus
		3.93	50	-22	-10		Posterior Middle Temporal Gyrus
	258	4.86	48	30	38	< 0.001	Middle Frontal Gyrus
		4.20	52	22	40		Middle Frontal Gyrus
		3.98	46	28	28		Middle Frontal Gyrus
		3.75	52	34	28		Middle Frontal Gyrus
	249	4.38	-54	-58	30	< 0.001	Angular Gyrus
		4.34	-46	-58	34		Angular Gyrus
		3.73	-56	-60	42		Superior Lateral Occipital Cortex
		3.37	-62	-58	28		Angular Gyrus
		3.18	-64	-54	26		Angular Gyrus
	231	4.64	-58	-34	0	0.001	Posterior Superior Temporal Gyrus
		4.49	-50	-30	-4		Posterior Middle Temporal Gyrus
		3.48	-48	-38	-4		Posterior Middle Temporal Gyrus
	139	4.26	-48	2	-36	0.012	Temporal Pole
		4.14	-44	2	-38		Temporal Pole
		3.76	-50	6	-30		Temporal Pole
		3.35	-62	-2	-24		Anterior Middle Temporal Gyrus
		3.32	-58	2	-28		Anterior Middle Temporal Gyrus
$S_{POS}P_{NEG}<S_{POS}P_{XX}$	4345	6.48	30	-58	-8	< 0.001	Temporal Occipital Fusiform Cortex
		6.38	30	-38	-18		Posterior Temporal Fusiform Cortex
		5.96	32	-46	-10		Temporal Occipital Fusiform Cortex
		5.82	50	-44	-18		Inferior Temporal Gyrus
		5.79	38	-84	18		Superior Lateral Occipital Cortex
		5.66	42	-66	-10		Inferior Lateral Occipital Cortex
	2927	6.30	-48	-60	0	< 0.001	Middle Temporal Gyrus
		6.20	-32	-34	-16		Posterior Parahippocampal Gyrus
		5.63	-30	-46	-8		Temporal Occipital Fusiform Cortex
		5.59	-56	-66	-4		Inferior Lateral Occipital Cortex
		5.41	-28	-46	-20		Temporal Occipital Fusiform Cortex
		5.38	-34	-44	-28		Temporal Occipital Fusiform Cortex
	1938	6.13	-8	-34	44	< 0.001	Posterior Cingulate Gyrus
		5.94	-12	-36	40		Posterior Cingulate Gyrus
		5.52	-10	-56	68		Superior Parietal Lobule
		5.39	16	-36	42		Precuneus Cortex
		5.27	6	-36	42		Posterior Cingulate Gyrus
		5.23	8	-38	52		Precuneus Cortex
	1600	5.75	-66	-30	30	< 0.001	Anterior Supramarginal Gyrus
		5.57	-60	-30	22		Parietal Operculum Cortex
		5.30	-34	6	10		Insular Cortex
		5.00	-36	2	10		Insular Cortex

	4.87	-58	-16	16		Central Opercular Cortex
	4.85	-42	2	6		Central Opercular Cortex
1139	5.62	64	-22	30	< 0.001	Anterior Supramarginal Gyrus
	5.29	62	-22	22		Anterior Supramarginal Gyrus
	5.12	62	-30	40		Anterior Supramarginal Gyrus
	4.98	64	-28	46		Anterior Supramarginal Gyrus
	4.64	64	-18	46		Postcentral Gyrus
	4.55	66	-14	8		Planum Temporale
1131	6.08	-34	-88	16	< 0.001	Superior Lateral Occipital Cortex
	5.12	-26	-88	16		Superior Lateral Occipital Cortex
	4.80	-32	-90	6		Occipital Pole
	4.74	-32	-90	2		Inferior Lateral Occipital Cortex
	4.54	-42	-78	24		Superior Lateral Occipital Cortex
	4.30	-20	-74	44		Superior Lateral Occipital Cortex
1012	6.46	-26	36	-14	< 0.001	Frontal Orbital Cortex
	5.88	22	36	-14		Frontal Pole
	5.49	-32	38	-6		Frontal Pole
	5.24	2	44	-12		Frontal Medial Cortex
	4.89	-6	38	-14		Frontal Medial Cortex
	4.66	32	36	-16		Frontal Pole
885	5.93	-8	-58	12	< 0.001	Precuneus Cortex
	5.45	-6	-60	18		Precuneus Cortex
	4.89	12	-50	10		Precuneus Cortex
	4.72	8	-58	16		Precuneus Cortex
	4.43	6	-56	4		Lingual Gyrus
	4.34	18	-44	2		Posterior Cingulate Gyrus
602	6.69	2	2	38	< 0.001	Anterior Cingulate Gyrus
	3.83	-16	10	32		Left Cerebral White Matter
	3.48	10	0	48		Supplementary Motor Cortex
505	5.08	38	2	4	< 0.001	Insular Cortex
	4.64	22	0	-12		Right Cerebral Cortex
	4.36	36	-16	4		Insular Cortex
	4.12	40	-8	-4		Insular Cortex
	4.09	34	10	10		Insular Cortex
	3.92	26	-10	6		Right Putamen
213	4.61	-46	-28	64	0.001	Postcentral Gyrus
	4.06	-38	-24	68		Postcentral Gyrus
	3.52	-32	-26	58		Precentral Gyrus
	3.51	-32	-26	74		Postcentral Gyrus
	3.45	-40	-40	66		Postcentral Gyrus
192	4.30	28	-20	-12	0.002	Right Hippocampus
	4.21	34	-14	-20		Anterior Parahippocampal Gyrus
	4.19	42	-12	-28		Posterior Temporal Fusiform Cortex
	4.03	22	-20	-14		Posterior Parahippocampal Gyrus
	3.59	36	-8	-38		Anterior Temporal Fusiform Cortex

	3.34	18	-22	-20		Anterior Parahippocampal Gyrus
152	5.11	52	40	12	0.008	Frontal Pole
148	4.69	18	-54	66	0.009	Superior Parietal Lobule
	4.20	16	-50	66		Superior Parietal Lobule
	3.95	18	-46	64		Superior Parietal Lobule
133	4.45	50	-28	62	0.015	Postcentral Gyrus
	3.98	28	-38	60		Postcentral Gyrus
	3.56	40	-32	66		Postcentral Gyrus
114	4.84	22	-74	46	0.031	Superior Lateral Occipital Cortex
	3.84	26	-72	38		Superior Lateral Occipital Cortex

Supplementary Table 5. Differences in brain responses to partner-directed feedback when simultaneous incongruent self-directed feedback is present versus absent

Contrast	Voxels	*Z value*	X	Y	Z	*p* *(cluster-GRF)*	Brain region
$[S_{NEG}P_{POS}\text{-}S_{NEG}P_{XX}] > [S_{XX}P_{POS}\text{-}S_{XX}P_{XX}]$	576	5.54	62	-32	44	< 0.001	Anterior Supramarginal Gyrus
		4.20	60	-50	42		Angular Gyrus
		4.11	66	-26	28		Anterior Supramarginal Gyrus
		3.90	68	-22	32		Anterior Supramarginal Gyrus
		3.60	64	-42	38		Posterior Supramarginal Gyrus
	371	5.49	36	32	50	< 0.001	Middle Frontal Gyrus
		4.21	26	22	56		Superior Frontal Gyrus
		3.76	24	18	64		Superior Frontal Gyrus
		3.63	44	28	46		Middle Frontal Gyrus
		3.32	30	30	40		Middle Frontal Gyrus
	358	4.98	12	-92	30	< 0.001	Occipital Pole
		4.38	8	-90	20		Occipital Pole
		4.19	18	-86	32		Superior Lateral Occipital Cortex
		3.79	24	-88	34		Occipital Pole
		3.70	16	-84	40		Superior Lateral Occipital Cortex
	301	5.24	36	50	-14	< 0.001	Frontal Pole
		5.14	46	48	-12		Frontal Pole
		4.11	42	60	-8		Frontal Pole
		3.42	50	48	-2		Frontal Pole
	233	5.52	-62	-32	44	0.001	Anterior Supramarginal Gyrus
		4.35	-62	-28	34		Anterior Supramarginal Gyrus
		3.31	-58	-40	52		Posterior Supramarginal Gyrus
	226	4.63	-8	-90	26	0.001	Occipital Pole
		3.79	-12	-80	30		Cuneal Cortex
	207	4.15	14	-72	-8	0.001	Lingual Gyrus
		4.09	8	-70	-6		Lingual Gyrus
		3.96	6	-72	0		Lingual Gyrus
		3.78	8	-76	-6		Lingual Gyrus
	170	4.79	4	-34	46	0.004	Posterior Cingulate Gyrus
		4.75	6	-30	44		Posterior Cingulate Gyrus
		3.38	14	-40	46		Precuneus Cortex
	165	5.23	-38	40	-10	0.005	Frontal Pole
		4.56	-40	46	-14		Frontal Pole
		3.87	-18	42	-16		Frontal Pole
		3.54	-30	50	-10		Frontal Pole
	155	4.63	-60	-56	-6	0.006	Middle Temporal Gyrus
		4.20	-64	-60	0		Middle Temporal Gyrus
		4.02	-62	-62	-8		Middle Temporal Gyrus
		3.57	-64	-46	-8		Middle Temporal Gyrus

	105	4.41	-24	16	56	0.040	Superior Frontal Gyrus
		3.81	-20	6	64		Superior Frontal Gyrus
		3.79	-22	10	64		Superior Frontal Gyrus
[$S_{NEG}P_{POS}$-$S_{NEG}P_{XX}$] < [$S_{XX}P_{POS}$-$S_{XX}P_{XX}$]	6868	7.42	36	-82	-12	< 0.001	Inferior Lateral Occipital Cortex
		7.05	-36	-88	-12		Inferior Lateral Occipital Cortex
		7.03	42	-56	-20		Temporal Occipital Fusiform Cortex
		6.97	40	-58	-16		Temporal Occipital Fusiform Cortex
		6.93	42	-66	-14		Occipital Fusiform Gyrus
		6.92	44	-78	-10		Inferior Lateral Occipital Cortex
	2669	6.19	-46	2	52	< 0.001	Precentral Gyrus
		6.17	-28	16	-16		Frontal Orbital Cortex
		5.70	-38	22	-16		Frontal Orbital Cortex
		5.55	-42	16	28		Middle Frontal Gyrus
		5.39	-40	28	-2		Frontal Orbital Cortex
		5.33	-34	-2	50		Precentral Gyrus
	2584	6.18	4	60	34	< 0.001	Frontal Pole
		6.06	-2	66	-2		Frontal Pole
		5.93	-4	48	-18		Frontal Medial Cortex
		5.76	-4	60	34		Frontal Pole
		5.62	4	60	26		Frontal Pole
		5.26	4	34	-6		Anterior Cingulate Gyrus
	2307	6.49	-4	-50	30	< 0.001	Posterior Cingulate Gyrus
		5.94	-2	-56	42		Precuneus Cortex
		5.78	8	-52	22		Posterior Cingulate Gyrus
		5.69	2	-58	46		Precuneus Cortex
		5.40	-22	-64	48		Superior Lateral Occipital Cortex
		4.93	-30	-74	30		Superior Lateral Occipital Cortex
	1007	5.84	40	18	30	< 0.001	Middle Frontal Gyrus
		5.79	46	14	32		Middle Frontal Gyrus
		5.68	52	26	24		Middle Frontal Gyrus
		5.48	48	20	30		Middle Frontal Gyrus
		5.09	42	2	58		Middle Frontal Gyrus
		4.67	36	0	60		Middle Frontal Gyrus
	859	6.06	34	-68	34	< 0.001	Superior Lateral Occipital Cortex
		4.89	20	-64	56		Superior Lateral Occipital Cortex
		4.80	24	-62	52		Superior Lateral Occipital Cortex
		4.77	28	-62	50		Superior Lateral Occipital Cortex
		4.76	26	-62	46		Superior Lateral Occipital Cortex
		4.68	30	-62	56		Superior Lateral Occipital Cortex
	794	5.76	-56	-38	4	< 0.001	Posterior Superior Temporal Gyrus
		5.18	-46	-42	4		Posterior Superior Temporal Gyrus
		5.02	-46	-46	12		Posterior Supramarginal Gyrus

		4.54	-50	-16	-8		Posterior Superior Temporal Gyrus
		4.39	-54	-50	18		Angular Gyrus
		3.52	-62	-56	18		Angular Gyrus
	574	6.84	-4	10	58	< 0.001	Superior Frontal Gyrus
		6.61	-2	8	64		Supplementary Motor Cortex
		5.15	-8	14	48		Paracingulate Gyrus
		3.38	-8	26	62		Superior Frontal Gyrus
	179	5.29	26	18	-18	0.003	Frontal Orbital Cortex
		4.45	36	26	-18		Frontal Orbital Cortex
	147	4.37	4	-24	58	0.008	Precentral Gyrus
		4.15	-2	-28	60		Precentral Gyrus
		3.36	-10	-26	56		Precentral Gyrus
		3.31	8	-18	54		Precentral Gyrus
	101	5.76	8	-72	-20	0.047	Lingual Gyrus
		4.42	-6	-74	-20		Occipital Fusiform Gyrus
$[S_{POS}P_{NEG}-S_{POS}P_{XX}] > [S_{XX}P_{NEG}-S_{XX}P_{XX}]$	347	6.01	40	50	-12	< 0.001	Frontal Pole
		4.29	40	60	-10		Frontal Pole
	161	4.62	56	-56	40	0.004	Angular Gyrus
		4.28	52	-64	46		Superior Lateral Occipital Cortex
		3.88	62	-44	46		Posterior Supramarginal Gyrus
		3.56	60	-50	42		Angular Gyrus
		3.41	52	-46	46		Posterior Supramarginal Gyrus
$[S_{POS}P_{NEG}-S_{POS}P_{XX}] < [S_{XX}P_{NEG}-S_{XX}P_{XX}]$	30792	7.94	-2	-62	38	< 0.001	Precuneus Cortex
		7.81	-42	-80	-10		Inferior Lateral Occipital Cortex
		7.81	36	-60	-18		Temporal Occipital Fusiform Cortex
		7.76	-4	-58	38		Precuneus Cortex
		7.71	-36	-48	-22		Temporal Occipital Fusiform Cortex
		7.70	-32	-54	-20		Temporal Occipital Fusiform Cortex
	4093	7.26	0	64	-2	< 0.001	Frontal Pole
		7.05	-6	50	-14		Frontal Medial Cortex
		7.04	0	48	-18		Frontal Medial Cortex
		6.79	-6	58	-4		Frontal Pole
		6.57	6	50	-14		Frontal Medial Cortex
		6.47	0	56	-10		Frontal Pole
	3740	6.04	-42	-10	58	< 0.001	Precentral Gyrus
		5.90	-40	-4	64		Precentral Gyrus
		5.82	-32	8	32		Middle Frontal Gyrus
		5.74	-38	-24	66		Postcentral Gyrus
		5.73	-48	0	56		Precentral Gyrus
		5.71	-48	14	28		Inferior Frontal Gyrus
	475	5.47	42	0	60	< 0.001	Middle Frontal Gyrus

	4.97	28	0	48		Precentral Gyrus
	4.51	38	0	44		Precentral Gyrus
	4.31	44	0	52		Precentral Gyrus
	4.28	34	0	56		Middle Frontal Gyrus
	4.21	28	-4	60		Superior Frontal Gyrus
329	5.93	32	-22	64	< 0.001	Precentral Gyrus
	4.23	40	-30	54		Postcentral Gyrus
	4.09	48	-28	60		Postcentral Gyrus
	3.92	38	-34	58		Postcentral Gyrus
	3.67	42	-14	66		Precentral Gyrus
	3.46	48	-14	60		Postcentral Gyrus
276	5.20	54	-8	-18	< 0.001	Posterior Middle Temporal Gyrus
	4.68	66	-4	-20		Anterior Middle Temporal Gyrus
209	4.77	42	22	28	0.001	Middle Frontal Gyrus
	4.31	46	28	20		Inferior Frontal Gyrus
	3.67	50	20	30		Middle Frontal Gyrus
	3.50	36	26	22		Middle Frontal Gyrus
166	5.31	-38	28	2	0.004	Frontal Operculum Cortex
	4.07	-42	26	10		Inferior Frontal Gyrus
164	4.64	50	-22	40	0.004	Postcentral Gyrus
	3.50	62	-16	46		Postcentral Gyrus
	3.49	56	-22	38		Anterior Supramarginal Gyrus
	3.38	58	-16	46		Postcentral Gyrus
	3.20	48	-28	30		Parietal Operculum Cortex
134	4.06	-28	22	44	0.012	Middle Frontal Gyrus
	3.83	-24	38	44		Frontal Pole
	3.72	-24	40	50		Frontal Pole
	3.64	-26	34	42		Middle Frontal Gyrus
	3.63	-16	38	38		Superior Frontal Gyrus
113	4.82	32	32	-14	0.026	Frontal Orbital Cortex
	4.02	26	32	-14		Frontal Orbital Cortex
	3.57	26	38	-20		Frontal Pole
97	4.94	-56	-22	18	0.049	Central Opercular Cortex
	4.68	-60	-16	16		Central Opercular Cortex
	3.36	-46	-20	16		Central Opercular Cortex

Supplementary Table 6. ROIs selected in regression analysis

Index	Neurosynth search term	Brain region	x	y	z
1	Empathy	Temporoparietal Junction	50	-50	18
2	Empathy	Dorsomedial Prefrontal Cortex	4	56	24
3	Empathy	Anterior Insula	40	18	-8
4	Empathy	Dorsal Anterior Cingulate Cortex	-4	26	26
5	Emotion regulation	Middle Frontal Gyrus	-36	18	52
6	Overcoming emotional egocentricity bias	Supramarginal Gyrus	68	-38	36

Supplementary Table 7. Summary of ROI regression analyses

Activity in $S_{NEG}P_{POS}$-$S_{NEG}P_{XX}$	Affective ratings ($S_{NEG}P_{POS}$-$S_{NEG}P_{XX}$)	Inferred partner affect for positive partner-directed feedback ($S_{XX}P_{POS}$)	Emotional similarity with the partner ($S_{NEG}P_{POS}$)	EC_IRI	PT_IRI	DERS-18	Daily support provided
Temporoparietal Junction (50, -50, 18)	-0.06 (0.517)	0.10 (0.262[a])	0.10 (0.240)	0.15 (0.097)	0.11 (0.225)	-0.09 (0.293)	0.04 (0.636)
Dorsomedial Prefrontal Cortex (4, 56, 24)	-0.12 (0.162)	0.17 (0.052)	0.14 (0.108)	-0.03 (0.713)	0.10 (0.256)	-0.25 (0.004**)	0.23 (0.008**)
Anterior Insula (40, 18, -8)	-0.12 (0.163)	0.12 (0.174[a])	-0.04 (0.669)	0.10 (0.236)	0.05 (0.595)	-0.08 (0.361)	0.08 (0.350)
Dorsal Anterior Cingulate Cortex (-4, 26, 26)	-0.05 (0.542)	0.03 (0.782[a])	0.05 (0.521)	0.18 (0.043*)	0.11 (0.222)	-0.10 (0.227)	0.09 (0.314)
Middle Frontal Gyrus (-36, 18, 52)	-0.03 (0.741)	0.19 (0.028*[a])	-0.05 (0.576)	-0.02 (0.82)	0.10 (0.250)	-0.01 (0.879)	-0.03 (0.712)
Supramarginal Gyrus (68, -38, 36)	-0.10 (0.270)	-0.05 (0.572[a])	-0.11 (0.198)	0.10 (0.246)	0.01 (0.876)	-0.02 (0.779)	0.04 (0.686)

Note. Correlation coefficients in the table are reported as standardized beta values with corresponding *p* values in parentheses.
[a]: Bootstrap-derived *p*-values based on 5,000 iterations due to deviation from model residual assumptions.

Supplementary Table 8. Summary of whole-brain multiple regression analysis for the $S_{NEG}P_{POS}$-$S_{NEG}P_{XX}$ contrast with empathic concern, emotion regulation, and daily support provided (Voxel-wise $p < 0.001$ and cluster-level GRF-corrected $p < 0.05$)

Behavior	Association	Voxels	*Z*	x	y	z	*p*	Brain region
EC_IRI	Pos	162	4.0	-20	-62	8	0.004	Intracalcarine Cortex
			3.9	-16	-68	10		Intracalcarine Cortex
			3.8	-8	-68	10		Intracalcarine Cortex
			3.6	-6	-76	14		Intracalcarine Cortex
			3.6	-10	-80	16		Intracalcarine Cortex
		107	4.3	12	8	36	0.033	Anterior Cingulate Gyrus
			4.0	10	16	40		Paracingulate Gyrus
			4.0	14	22	34		Paracingulate Gyrus
			3.8	10	4	30		Anterior Cingulate Gyrus
			3.4	6	22	34		Anterior Cingulate Gyrus
DERS-18	Neg	226	4.1	10	54	6	0.000	Paracingulate Gyrus
			4.1	4	52	8		Paracingulate Gyrus
			3.9	6	54	20		Superior Frontal Gyrus
			3.7	-2	62	12		Frontal Pole
			3.6	2	48	-2		Paracingulate Gyrus
			3.4	10	56	30		Frontal Pole
Daily support provided	Pos	569	5.2	40	-66	8	0.000	Inferior Lateral Occipital Cortex
			4.6	52	-70	4		Inferior Lateral Occipital Cortex
			4.2	50	-46	-2		Middle Temporal Gyrus
			4.1	38	-62	18		Superior Lateral Occipital Cortex
			4.0	40	-54	12		Middle Temporal Gyrus
			3.9	54	-58	6		Middle Temporal Gyrus
		152	4.1	-50	-78	14	0.006	Inferior Lateral Occipital Cortex
			4.1	-46	-62	10		Middle Temporal Gyrus
			3.8	-40	-60	12		Middle Temporal Gyrus
			3.8	-42	-64	16		Superior Lateral Occipital Cortex
		136	4.1	20	-82	22	0.010	Superior Lateral Occipital Cortex
			3.9	22	-78	14		Superior Lateral Occipital Cortex
			3.4	22	-88	32		Occipital Pole
			3.4	22	-94	22		Occipital Pole

Supplementary Table 9. Summary of whole-brain multiple regression analysis for the $S_{NEG}P_{POS}$-$S_{NEG}P_{XX}$ contrast with behavioral empathy, trait empathy, emotion regulation, and daily support provided (Voxel-wise *p* < 0.005 and cluster size ≥ 15)

Behavior	Association	Voxels	*Z*	x	y	z	Brain region
Affective ratings ($S_{NEG}P_{POS}$-$S_{NEG}P_{XX}$)	Pos	18	3.2	-18	2	42	Left Cerebral White Matter
		15	2.9	-38	-34	-8	Posterior Temporal Fusiform Cortex
	Neg	262	3.8	-18	24	50	Superior Frontal Gyrus
			3.7	-24	14	60	Superior Frontal Gyrus
			3.7	-20	14	60	Superior Frontal Gyrus
			3.6	-24	2	62	Superior Frontal Gyrus
			3.6	-22	22	50	Superior Frontal Gyrus
			3.1	-30	4	62	Middle Frontal Gyrus
		227	4.3	54	-54	-8	Inferior Temporal Gyrus
			3.5	58	-64	-8	Inferior Lateral Occipital Cortex
			3.2	46	-50	2	Middle Temporal Gyrus
			3.2	54	-70	-4	Inferior Lateral Occipital Cortex
			3.1	62	-56	-10	Middle Temporal Gyrus
			2.9	50	-78	-2	Inferior Lateral Occipital Cortex
		106	3.5	-38	-38	40	Anterior Supramarginal Gyrus
			3.5	-46	-40	44	Anterior Supramarginal Gyrus
		101	3.5	-32	34	32	Middle Frontal Gyrus
			3.5	-32	24	26	Middle Frontal Gyrus
			3.5	-32	30	30	Middle Frontal Gyrus
			3.2	-32	26	32	Middle Frontal Gyrus
			3.2	-28	42	34	Frontal Pole
			3.0	-28	40	28	Frontal Pole
		64	3.2	38	-52	50	Superior Parietal Lobule
			2.9	32	-64	62	Superior Lateral Occipital Cortex
			2.9	36	-60	60	Superior Lateral Occipital Cortex
		62	3.3	2	36	58	Superior Frontal Gyrus
			3.2	8	30	54	Superior Frontal Gyrus
			3.0	10	34	48	Superior Frontal Gyrus
		59	3.5	28	42	36	Frontal Pole
			3.0	22	32	34	Superior Frontal Gyrus
			3.0	32	40	38	Frontal Pole
			2.8	28	24	38	Middle Frontal Gyrus
			2.8	32	30	36	Middle Frontal Gyrus
			2.8	28	30	38	Middle Frontal Gyrus
		54	3.8	-48	6	32	Precentral Gyrus
		50	3.2	34	-6	44	Precentral Gyrus

			3.1	42	-4	44	Precentral Gyrus
		48	3.7	12	64	-10	Frontal Pole
			3.2	22	58	-10	Frontal Pole
		43	3.4	24	10	64	Superior Frontal Gyrus
			3.2	22	10	68	Superior Frontal Gyrus
			2.9	24	16	60	Superior Frontal Gyrus
		39	3.6	-34	-4	-12	Left Cerebral White Matter
		35	3.5	68	-40	12	Posterior Supramarginal Gyrus
		30	3.1	-32	60	16	Frontal Pole
			3.0	-30	56	16	Frontal Pole
		27	3.1	34	-76	40	Superior Lateral Occipital Cortex
			3.0	38	-76	36	Superior Lateral Occipital Cortex
		27	3.6	-42	-20	-32	Posterior Temporal Fusiform Cortex
		26	3.1	8	-70	56	Precuneus Cortex
		25	3.4	34	-50	-22	Temporal Occipital Fusiform Cortex
		25	2.8	-46	44	6	Frontal Pole
			2.8	-42	42	2	Frontal Pole
		25	3.0	52	26	24	Middle Frontal Gyrus
			3.0	56	28	30	Middle Frontal Gyrus
		20	3.2	-32	-8	-34	Anterior Temporal Fusiform Cortex
			2.9	-28	-14	-34	Anterior Parahippocampal Gyrus
		19	3.3	14	42	-2	Paracingulate Gyrus
			2.7	8	48	2	Paracingulate Gyrus
		17	3.2	26	24	-12	Frontal Orbital Cortex
		15	2.9	28	-46	44	Superior Parietal Lobule
		15	3.2	38	22	24	Middle Frontal Gyrus
Rating for partner affect ($S_{XX}P_{POS}$)	Pos	353	4.0	-18	-48	38	Precuneus Cortex
			3.7	-16	-54	44	Precuneus Cortex
			3.4	-16	-60	28	Precuneus Cortex
			3.3	-26	-62	30	Superior Lateral Occipital Cortex
			3.3	-8	-66	40	Precuneus Cortex
			3.3	-22	-58	30	Precuneus Cortex
		301	4.0	8	-60	40	Precuneus Cortex
			3.6	8	-62	30	Precuneus Cortex
			3.2	16	-58	28	Precuneus Cortex
			3.0	16	-54	40	Precuneus Cortex
			2.8	10	-68	44	Precuneus Cortex
			2.7	16	-64	32	Precuneus Cortex
		167	3.9	-46	-62	-10	Inferior Temporal Gyrus
			3.2	-52	-50	-12	Inferior Temporal Gyrus
			3.2	-50	-58	-16	Inferior Temporal Gyrus
		162	3.3	36	-48	48	Superior Parietal Lobule

	3.2	30	-54	40	Superior Parietal Lobule
	3.2	40	-52	54	Angular Gyrus
	2.9	28	-54	44	Superior Parietal Lobule
	2.6	24	-54	46	Superior Parietal Lobule
133	3.8	40	-66	52	Superior Lateral Occipital Cortex
	3.0	50	-58	54	Superior Lateral Occipital Cortex
	3.0	42	-62	42	Superior Lateral Occipital Cortex
	2.8	34	-64	30	Superior Lateral Occipital Cortex
	2.7	34	-64	48	Superior Lateral Occipital Cortex
	2.7	36	-64	38	Superior Lateral Occipital Cortex
120	3.6	50	22	36	Middle Frontal Gyrus
	3.3	50	28	34	Middle Frontal Gyrus
	3.1	54	26	36	Middle Frontal Gyrus
	3.0	50	34	34	Middle Frontal Gyrus
95	3.6	4	-34	-8	Brain-Stem
	3.6	-8	-26	-18	Brain-Stem
	3.4	4	-30	-10	Brain-Stem
	3.3	6	-26	-18	Brain-Stem
	3.2	6	-16	-24	Brain-Stem
89	3.8	16	-26	-12	Posterior Parahippocampal Gyrus
	3.6	2	-22	-4	Right Thalamus
65	3.5	-14	42	20	Paracingulate Gyrus
	3.3	-12	42	14	Paracingulate Gyrus
64	3.5	24	44	44	Frontal Pole
	3.4	22	34	36	Superior Frontal Gyrus
	2.8	26	32	44	Middle Frontal Gyrus
56	3.1	-32	-70	44	Superior Lateral Occipital Cortex
	3.0	-30	-68	50	Superior Lateral Occipital Cortex
51	3.6	-42	-4	38	Precentral Gyrus
	3.1	-34	-6	34	Precentral Gyrus
49	3.7	32	-28	-10	Right Hippocampus
	2.9	36	-22	-8	Right Cerebral White Matter
48	3.6	14	-16	12	Right Thalamus
43	3.5	-10	-52	-24	Cerebellar lobule V
37	3.2	46	42	-12	Frontal Pole
35	3.1	6	-40	20	Posterior Cingulate Gyrus
	3.1	8	-36	18	Posterior Cingulate Gyrus
34	4.1	-42	-8	-36	Anterior Inferior Temporal Gyrus
33	3.3	42	-40	-28	Temporal Occipital Fusiform Cortex
	3.2	46	-40	-24	Inferior Temporal Gyrus
	2.9	36	-40	-30	Temporal Occipital Fusiform Cortex
33	4.0	-24	2	36	Left Cerebral White Matter
33	3.2	42	18	22	Inferior Frontal Gyrus

		29	3.8	-48	40	-18	Frontal Pole
			2.6	-44	48	-16	Frontal Pole
		28	3.6	2	-32	-28	Brain-Stem
		28	3.5	34	6	48	Middle Frontal Gyrus
			3.4	36	4	52	Middle Frontal Gyrus
		27	3.7	-8	58	-20	Frontal Pole
			3.2	-4	56	-24	Frontal Pole
			3.1	-16	58	-18	Frontal Pole
		27	3.7	38	12	-30	Temporal Pole
		23	3.8	-8	-46	14	Posterior Cingulate Gyrus
		23	3.8	-32	-80	-6	Occipital Fusiform Gyrus
		23	3.7	-12	46	-18	Frontal Pole
		22	3.7	-30	-14	-12	Left Cerebral White Matter
		18	3.1	26	-82	10	Superior Lateral Occipital Cortex
			2.8	24	-88	8	Superior Lateral Occipital Cortex
		18	3.0	-22	-20	-20	Anterior Parahippocampal Gyrus
			3.0	-28	-16	-22	Anterior Parahippocampal Gyrus
		17	3.2	30	10	30	Middle Frontal Gyrus
		16	3.3	-42	18	18	Inferior Frontal Gyrus
		16	3.3	-14	-16	-16	Left Hippocampus
		16	3.1	-12	14	42	Paracingulate Gyrus
		16	3.1	58	-50	-14	Inferior Temporal Gyrus
			2.7	52	-48	-14	Inferior Temporal Gyrus
		16	3.3	38	-2	32	Precentral Gyrus
Emotional similarity with the partner (SNEGPPOS)	Pos	629	4.1	12	50	-10	Frontal Medial Cortex
			3.9	12	50	-14	Frontal Medial Cortex
			3.7	-4	34	-4	Anterior Cingulate Gyrus
			3.6	-8	40	6	Anterior Cingulate Gyrus
			3.6	4	34	-4	Anterior Cingulate Gyrus
			3.4	-2	66	-2	Frontal Pole
		215	4.3	-14	6	-14	Frontal Orbital Cortex
			4.1	-26	8	-22	Frontal Orbital Cortex
			4.0	-26	14	-20	Frontal Orbital Cortex
			3.1	-22	-4	-12	Left Amygdala
			3.1	-22	16	-12	Frontal Orbital Cortex
		149	4.5	-8	-50	8	Posterior Cingulate Gyrus
			3.1	-12	-56	2	Lingual Gyrus
			3.1	-20	-58	18	Precuneus Cortex
			3.0	-16	-58	14	Precuneus Cortex
			2.9	-4	-58	10	Precuneus Cortex
			2.7	-2	-46	12	Posterior Cingulate Gyrus

118	3.5	-4	-64	22	Precuneus Cortex
	3.4	2	-66	24	Precuneus Cortex
115	3.7	62	-36	-2	Posterior Middle Temporal Gyrus
	3.7	58	-32	2	Posterior Superior Temporal Gyrus
	3.3	54	-46	6	Middle Temporal Gyrus
	3.3	56	-44	2	Middle Temporal Gyrus
115	4.1	-42	10	-30	Temporal Pole
	3.6	-46	16	-20	Temporal Pole
	3.3	-40	20	-20	Frontal Orbital Cortex
	3.2	-38	24	-26	Temporal Pole
	3.1	-38	20	-40	Temporal Pole
	2.9	-36	16	-28	Temporal Pole
98	4.0	-26	-62	-18	Temporal Occipital Fusiform Cortex
	3.3	-24	-54	-16	Temporal Occipital Fusiform Cortex
92	3.3	2	-14	0	Right Thalamus
	3.2	2	-6	14	Right Cerebral White Matter
	3.0	6	-12	8	Right Thalamus
	2.9	2	-16	16	Right Cerebral White Matter
90	4.0	-8	16	-8	Subcallosal Cortex
	3.4	-4	6	0	Left Lateral Ventricle
89	4.0	-14	-48	56	Precuneus Cortex
	3.3	-8	-40	52	Precuneus Cortex
	2.8	-2	-36	56	Precentral Gyrus
	2.8	-4	-40	54	Precuneus Cortex
87	3.7	-6	-34	76	Postcentral Gyrus
	3.2	6	-26	74	Precentral Gyrus
	3.2	4	-38	70	Postcentral Gyrus
	3.1	-2	-26	74	Precentral Gyrus
	2.7	6	-30	68	Precentral Gyrus
77	3.1	18	-52	10	Precuneus Cortex
	3.1	14	-48	0	Lingual Gyrus
	3.1	14	-52	4	Precuneus Cortex
71	4.7	-20	-68	32	Superior Lateral Occipital Cortex
	3.1	-22	-62	34	Superior Lateral Occipital Cortex
	3.0	-28	-62	30	Superior Lateral Occipital Cortex
71	3.8	10	6	-14	Frontal Orbital Cortex
	3.6	18	-2	-14	Right Amygdala
68	3.6	58	8	-10	Temporal Pole
	2.9	58	4	-2	Planum Polare
64	3.2	54	16	-30	Temporal Pole
	3.1	46	18	-30	Temporal Pole
	3.0	44	24	-32	Temporal Pole
	2.9	56	8	-28	Temporal Pole

62	3.4	-28	38	12	Frontal Pole
	3.4	-24	36	10	Left Cerebral White Matter
62	3.9	-62	-12	-12	Posterior Middle Temporal Gyrus
	3.0	-68	-18	-6	Posterior Superior Temporal Gyrus
56	3.5	-30	-44	-22	Posterior Temporal Fusiform Cortex
52	3.2	-14	-42	-8	Lingual Gyrus
	3.1	-4	-36	-8	Brain-Stem
	3.1	-14	-42	-14	Posterior Parahippocampal Gyrus
	3.0	-8	-34	-4	Brain-Stem
50	3.0	56	-4	-14	Anterior Superior Temporal Gyrus
	3.0	62	-4	-16	Anterior Middle Temporal Gyrus
	2.9	66	-4	-24	Anterior Middle Temporal Gyrus
46	3.6	50	-72	28	Superior Lateral Occipital Cortex
43	3.9	-24	28	-12	Frontal Orbital Cortex
42	3.2	-28	-50	58	Superior Parietal Lobule
	3.0	-26	-40	60	Postcentral Gyrus
41	3.4	-30	-90	-4	Inferior Lateral Occipital Cortex
41	3.8	22	8	-22	Frontal Orbital Cortex
41	3.7	-4	-50	-16	Cerebellar lobule I-IV
36	3.1	18	-46	60	Superior Parietal Lobule
	2.8	26	-52	62	Superior Parietal Lobule
	2.7	24	-48	58	Superior Parietal Lobule
35	3.2	40	-12	22	Central Opercular Cortex
34	3.1	-58	-66	20	Superior Lateral Occipital Cortex
	2.8	-46	-80	16	Superior Lateral Occipital Cortex
	2.8	-50	-74	18	Superior Lateral Occipital Cortex
32	3.3	62	-62	14	Inferior Lateral Occipital Cortex
	3.1	58	-64	16	Superior Lateral Occipital Cortex
	3.0	60	-60	20	Superior Lateral Occipital Cortex
30	3.1	40	34	-16	Frontal Pole
	2.8	34	36	-14	Frontal Pole
29	3.5	2	-66	-16	Cerebellar Vermis VI
28	3.2	-12	-54	38	Precuneus Cortex
27	3.0	16	48	18	Paracingulate Gyrus
	2.9	14	44	20	Paracingulate Gyrus
	2.7	4	50	16	Paracingulate Gyrus
	2.7	10	52	18	Paracingulate Gyrus
27	3.6	-18	-30	4	Left Thalamus
26	3.5	38	-10	42	Precentral Gyrus
25	3.2	16	-70	4	Intracalcarine Cortex
	2.8	18	-76	2	Intracalcarine Cortex
25	3.1	-14	8	42	Supplementary Motor Cortex
	3.0	-12	2	42	Supplementary Motor Cortex

		24	3.4	18	-60	38	Precuneus Cortex
		22	3.4	46	-12	-14	Posterior Superior Temporal Gyrus
			3.0	40	-6	-16	Planum Polare
		22	3.0	-50	-70	32	Superior Lateral Occipital Cortex
			2.9	-48	-74	32	Superior Lateral Occipital Cortex
		21	3.2	-38	32	-2	Frontal Orbital Cortex
		20	3.9	38	-36	-20	Posterior Temporal Fusiform Cortex
		20	3.3	48	-6	-4	Planum Polare
		20	3.8	-8	-62	-22	Cerebellar lobule VI
		20	3.6	-28	8	-2	Left Putamen
		18	3.2	-22	-28	48	Precentral Gyrus
		18	3.3	30	-28	18	Planum Temporale
		18	3.3	-38	-32	2	Planum Temporale
		16	3.4	-34	-16	-4	Insular Cortex
		16	3.0	-48	-46	10	Posterior Supramarginal Gyrus
		16	3.3	16	-26	-8	Posterior Parahippocampal Gyrus
		16	3.2	-8	-32	36	Posterior Cingulate Gyrus
		16	3.0	14	-38	8	Posterior Parahippocampal Gyrus
			2.7	12	-34	4	Posterior Cingulate Gyrus
		16	3.1	16	-54	-6	Lingual Gyrus
		15	3.3	34	-82	-20	Occipital Fusiform Gyrus
EC_IRI	Pos	911	4.0	-20	-62	8	Intracalcarine Cortex
			3.9	-16	-68	10	Intracalcarine Cortex
			3.8	-8	-68	10	Intracalcarine Cortex
			3.7	-16	-86	24	Superior Lateral Occipital Cortex
			3.6	-6	-76	14	Intracalcarine Cortex
			3.6	-10	-80	16	Intracalcarine Cortex
		334	4.3	12	8	36	Anterior Cingulate Gyrus
			4.0	10	16	40	Paracingulate Gyrus
			4.0	14	22	34	Paracingulate Gyrus
			3.8	10	4	30	Anterior Cingulate Gyrus
			3.4	6	22	34	Anterior Cingulate Gyrus
			3.3	10	34	30	Paracingulate Gyrus
		238	3.5	-36	-2	48	Precentral Gyrus
			3.3	-46	0	50	Precentral Gyrus
			3.2	-46	0	58	Precentral Gyrus
			3.1	-42	-2	62	Middle Frontal Gyrus
			3.1	-38	4	46	Middle Frontal Gyrus
			3.0	-24	0	46	Superior Frontal Gyrus
		222	3.7	2	16	64	Superior Frontal Gyrus
			3.4	6	8	60	Supplementary Motor Cortex
			3.3	0	6	68	Supplementary Motor Cortex
			3.2	0	10	68	Supplementary Motor Cortex

	3.2	-8	8	58	Supplementary Motor Cortex
	3.1	-6	-4	60	Supplementary Motor Cortex
217	4.0	18	-2	58	Superior Frontal Gyrus
	3.8	20	4	56	Superior Frontal Gyrus
	2.8	28	-2	50	Precentral Gyrus
	2.7	32	0	58	Middle Frontal Gyrus
214	4.2	50	6	2	Central Opercular Cortex
	3.6	36	8	2	Insular Cortex
	3.4	56	8	14	Precentral Gyrus
	3.1	48	14	-2	Frontal Operculum Cortex
	3.1	50	10	6	Inferior Frontal Gyrus
	2.9	58	0	2	Planum Polare
198	4.0	40	-8	44	Precentral Gyrus
	3.9	46	-4	54	Precentral Gyrus
	3.8	42	-2	52	Precentral Gyrus
	3.3	44	-10	50	Precentral Gyrus
164	3.6	-42	6	-2	Insular Cortex
	3.5	-36	4	-2	Insular Cortex
	3.3	-34	4	-6	Insular Cortex
	3.2	-54	12	-2	Inferior Frontal Gyrus
	3.0	-42	8	6	Central Opercular Cortex
	2.9	-32	2	4	Left Cerebral White Matter
157	3.9	14	26	0	Right Lateral Ventricle
	3.3	22	6	-6	Right Putamen
	3.2	24	14	2	Right Putamen
	2.9	20	8	0	Right Putamen
	2.8	22	22	-12	Frontal Orbital Cortex
155	3.6	-66	-36	32	Anterior Supramarginal Gyrus
	3.3	-52	-34	28	Parietal Operculum Cortex
	3.3	-54	-34	18	Parietal Operculum Cortex
	3.1	-58	-34	22	Parietal Operculum Cortex
	3.1	-56	-26	22	Parietal Operculum Cortex
	2.8	-60	-18	22	Postcentral Gyrus
117	4.4	-48	-80	8	Inferior Lateral Occipital Cortex
	3.8	-56	-72	6	Inferior Lateral Occipital Cortex
	2.8	-42	-72	12	Inferior Lateral Occipital Cortex
113	3.6	14	-58	-4	Lingual Gyrus
	3.3	22	-54	-4	Lingual Gyrus
	3.1	20	-62	12	Supracalcarine Cortex
	3.1	16	-58	4	Lingual Gyrus
	2.8	22	-54	8	Precuneus Cortex
108	3.8	-46	38	30	Frontal Pole
	2.8	-40	42	36	Frontal Pole

	2.8	-38	40	40	Frontal Pole
	2.7	-36	32	36	Middle Frontal Gyrus
98	3.4	-16	-78	46	Superior Lateral Occipital Cortex
	3.3	-20	-76	44	Superior Lateral Occipital Cortex
	3.0	-24	-84	38	Superior Lateral Occipital Cortex
92	3.7	30	-32	66	Postcentral Gyrus
	3.0	36	-32	62	Postcentral Gyrus
	2.9	26	-24	68	Precentral Gyrus
	2.9	32	-30	72	Postcentral Gyrus
88	3.4	-56	-42	6	Posterior Superior Temporal Gyrus
	3.2	-46	-42	8	Posterior Superior Temporal Gyrus
	3.0	-50	-36	2	Posterior Superior Temporal Gyrus
86	3.5	58	-24	30	Anterior Supramarginal Gyrus
	3.4	62	-22	28	Anterior Supramarginal Gyrus
	3.3	54	-24	26	Anterior Supramarginal Gyrus
	2.7	48	-24	32	Anterior Supramarginal Gyrus
77	3.8	10	-18	40	Posterior Cingulate Gyrus
	3.6	6	-18	46	Posterior Cingulate Gyrus
	2.7	2	-12	42	Anterior Cingulate Gyrus
67	3.4	22	-58	60	Superior Lateral Occipital Cortex
	3.0	14	-60	62	Superior Lateral Occipital Cortex
	2.8	10	-56	62	Precuneus Cortex
63	3.2	-40	-14	46	Precentral Gyrus
	3.2	-44	-12	46	Precentral Gyrus
	3.2	-34	-16	42	Precentral Gyrus
57	3.6	12	40	10	Anterior Cingulate Gyrus
	3.4	6	38	14	Anterior Cingulate Gyrus
56	3.5	-24	-62	64	Superior Lateral Occipital Cortex
	3.1	-20	-52	60	Superior Parietal Lobule
	3.1	-16	-50	60	Superior Parietal Lobule
	2.6	-24	-60	56	Superior Lateral Occipital Cortex
55	3.2	-34	-54	-24	Temporal Occipital Fusiform Cortex
	3.2	-18	-68	-22	Left Cerebral Cortex
	3.1	-32	-62	-22	Temporal Occipital Fusiform Cortex
	2.8	-24	-66	-20	Temporal Occipital Fusiform Cortex
55	3.9	26	-24	6	Right Cerebral White Matter
	3.8	20	-18	0	Right Cerebral White Matter
54	3.2	18	-64	52	Superior Lateral Occipital Cortex
52	3.2	-6	20	30	Anterior Cingulate Gyrus
	3.0	-6	24	18	Anterior Cingulate Gyrus
52	3.3	-34	50	-14	Frontal Pole
	3.2	-26	52	-14	Frontal Pole
	2.9	-26	40	-18	Frontal Pole

		49	3.6	16	18	16	Right Caudate
		47	3.5	22	30	8	Right Cerebral White Matter
			3.1	24	36	6	Right Cerebral White Matter
		43	3.2	-26	-24	8	Left Cerebral White Matter
			3.1	-32	-18	8	Insular Cortex
			3.1	-38	-24	4	Heschl's Gyrus (includes H1 and H2)
		41	3.8	12	-40	-6	Lingual Gyrus
		38	3.1	40	42	16	Frontal Pole
		38	3.1	-40	-18	-6	Planum Polare
			3.0	-32	-22	-4	Left Cerebral White Matter
		37	3.3	24	-80	22	Superior Lateral Occipital Cortex
			2.8	24	-84	28	Superior Lateral Occipital Cortex
		32	3.5	56	-30	2	Posterior Superior Temporal Gyrus
		28	3.1	-28	54	12	Frontal Pole
			2.7	-22	60	8	Frontal Pole
		27	3.6	32	-8	10	Insular Cortex
			2.7	24	-4	10	Right Putamen
		24	3.4	22	-26	32	Right Cerebral White Matter
		23	3.7	18	-2	6	Right Pallidum
		23	3.1	62	-44	12	Posterior Supramarginal Gyrus
			3.0	60	-46	8	Middle Temporal Gyrus
		22	3.3	18	20	54	Superior Frontal Gyrus
		22	3.4	4	-2	10	Right Lateral Ventricle
		21	2.9	-8	54	8	Paracingulate Gyrus
			2.9	-8	50	6	Paracingulate Gyrus
		19	3.0	-34	60	18	Frontal Pole
			3.0	-36	60	14	Frontal Pole
		19	3.3	48	-46	8	Middle Temporal Gyrus
			2.8	52	-48	12	Middle Temporal Gyrus
		19	3.7	-36	-42	-32	Left Cerebral Cortex
			2.9	-32	-44	-32	Cerebellar lobule VI
		18	2.9	24	-24	56	Precentral Gyrus
			2.8	22	-30	54	Precentral Gyrus
		18	3.1	-6	12	34	Anterior Cingulate Gyrus
			2.8	-8	4	36	Anterior Cingulate Gyrus
		16	3.3	-16	-36	56	Postcentral Gyrus
		15	3.6	-4	-60	-22	Cerebellar lobule V
	Neg	19	3.2	-34	-62	30	Superior Lateral Occipital Cortex
PT_IRI	Pos	357	5.4	-38	-44	-28	Temporal Occipital Fusiform Cortex
			3.9	-22	-50	-28	Cerebellar lobule VI
			3.6	-28	-56	-24	Left Cerebral Cortex
			3.6	-26	-62	-24	Cerebellar lobule VI
			3.4	-16	-64	-24	Cerebellar lobule VI

	3.1	-16	-58	-22	Cerebellar lobule VI
354	4.4	10	22	12	Right Cerebral White Matter
	4.0	22	30	8	Right Cerebral White Matter
	3.7	14	24	0	Right Caudate
	3.7	28	28	-12	Frontal Orbital Cortex
	3.6	24	26	-2	Frontal Orbital Cortex
	3.5	24	24	-10	Frontal Orbital Cortex
301	3.9	36	-44	-30	Temporal Occipital Fusiform Cortex
	3.8	40	-50	-24	Temporal Occipital Fusiform Cortex
	3.7	36	-62	-18	Temporal Occipital Fusiform Cortex
	3.7	38	-40	-30	Temporal Occipital Fusiform Cortex
	3.4	22	-36	-26	Brain-Stem
	3.1	48	-56	-14	Inferior Temporal Gyrus
176	3.8	44	-24	-10	Posterior Middle Temporal Gyrus
	3.5	34	-30	-12	Posterior Parahippocampal Gyrus
	3.3	50	-26	-6	Posterior Middle Temporal Gyrus
	3.2	44	-34	-12	Posterior Temporal Fusiform Cortex
	2.9	38	-36	-16	Posterior Temporal Fusiform Cortex
	2.8	50	-20	-12	Posterior Middle Temporal Gyrus
158	3.9	-36	34	24	Middle Frontal Gyrus
	3.2	-34	36	32	Middle Frontal Gyrus
	3.0	-36	32	36	Middle Frontal Gyrus
	2.9	-50	28	24	Middle Frontal Gyrus
156	3.6	18	-46	62	Superior Parietal Lobule
	3.5	26	-52	64	Superior Parietal Lobule
	3.3	30	-58	54	Superior Lateral Occipital Cortex
	2.9	32	-60	60	Superior Lateral Occipital Cortex
146	4.3	-54	-24	-4	Posterior Superior Temporal Gyrus
139	3.9	24	8	28	Right Cerebral White Matter
	3.4	30	4	26	Right Cerebral White Matter
	3.3	16	0	28	Right Cerebral White Matter
	2.9	12	8	28	Right Cerebral White Matter
	2.8	26	10	20	Right Cerebral White Matter
126	4.2	-24	-58	20	Supracalcarine Cortex
	3.8	-18	-54	20	Precuneus Cortex
	3.8	-24	-58	14	Precuneus Cortex
122	4.3	-2	-30	-12	Brain-Stem
	3.5	4	-20	-6	Right Cerebral White Matter
	3.4	0	-24	-8	Brain-Stem
122	4.1	20	-20	18	Right Cerebral White Matter
	3.4	14	-10	24	Right Lateral Ventricle
105	4.1	-26	66	4	Frontal Pole
	3.9	-20	60	8	Frontal Pole

	2.9	-28	58	8	Frontal Pole
	2.8	-36	62	-2	Frontal Pole
105	3.4	28	-86	10	Superior Lateral Occipital Cortex
	3.2	34	-86	14	Superior Lateral Occipital Cortex
	2.9	40	-86	2	Inferior Lateral Occipital Cortex
100	4.2	28	-34	10	Right Cerebral White Matter
	3.7	26	-30	4	Right Cerebral White Matter
	3.0	16	-26	0	Right Thalamus
96	3.7	26	-22	32	Right Cerebral White Matter
	3.1	30	-34	30	Right Cerebral White Matter
	3.0	34	-30	30	Right Cerebral White Matter
	3.0	20	-18	32	Right Cerebral White Matter
88	3.6	-6	-6	62	Supplementary Motor Cortex
	3.4	-4	-6	66	Supplementary Motor Cortex
	2.8	2	2	62	Supplementary Motor Cortex
65	4.2	36	-16	30	Right Cerebral White Matter
59	3.9	32	-50	24	Right Cerebral White Matter
53	3.8	22	-10	0	Right Pallidum
	3.4	18	-12	-2	Right Cerebral White Matter
	3.2	20	-2	6	Right Pallidum
	3.0	18	-6	-8	Right Cerebral White Matter
53	3.7	0	-48	-12	Cerebellar lobule I-IV
	2.8	0	-50	-4	Cerebellar lobule I-IV
53	4.1	-34	-4	-34	Anterior Temporal Fusiform Cortex
43	3.3	-24	-22	-12	Posterior Parahippocampal Gyrus
	2.9	-22	-22	-6	Left Cerebral White Matter
	2.8	-12	-14	-4	Left Cerebral White Matter
	2.7	-20	-20	-2	Left Cerebral White Matter
41	3.1	-44	24	36	Middle Frontal Gyrus
	3.1	-40	22	38	Middle Frontal Gyrus
	2.8	-36	22	28	Middle Frontal Gyrus
38	3.4	6	-8	12	Right Thalamus
	2.9	0	-18	10	Left Thalamus
35	3.6	-38	-26	30	Left Cerebral White Matter
35	3.5	32	-18	-10	Right Cerebral White Matter
35	3.9	10	38	12	Anterior Cingulate Gyrus
33	3.4	-18	-20	18	Left Cerebral White Matter
32	3.2	-12	8	20	Left Lateral Ventricle
	3.1	-12	2	18	Left Caudate
	2.8	-12	8	26	Left Cerebral White Matter
32	3.5	22	-22	54	Precentral Gyrus
	2.8	22	-32	52	Precentral Gyrus
32	3.2	64	-34	-6	Posterior Middle Temporal Gyrus

	2.9	56	-38	-6	Middle Temporal Gyrus
31	4.1	24	-4	38	Right Cerebral White Matter
30	4.0	-26	2	18	Left Cerebral White Matter
29	3.2	4	8	0	Subcallosal Cortex
	2.9	10	10	6	Right Caudate
27	3.8	38	-84	-18	Inferior Lateral Occipital Cortex
27	3.1	26	48	6	Frontal Pole
	3.1	22	46	6	Frontal Pole
	2.8	32	46	10	Frontal Pole
27	3.2	-66	-48	-2	Middle Temporal Gyrus
	2.7	-68	-50	6	Middle Temporal Gyrus
25	3.6	-22	-34	-6	Left Hippocampus
24	3.8	-6	-30	34	Posterior Cingulate Gyrus
24	3.7	34	44	-12	Frontal Pole
24	3.3	2	-86	-8	Lingual Gyrus
23	4.0	-4	-10	-2	Left Thalamus
23	3.4	0	-52	-24	Cerebellar lobule I-IV
	3.1	-4	-62	-22	Cerebellar lobule V
23	3.2	-16	-60	66	Superior Lateral Occipital Cortex
	3.1	-20	-58	68	Superior Parietal Lobule
23	3.4	22	-76	18	Superior Lateral Occipital Cortex
23	3.4	-48	16	-36	Temporal Pole
	2.7	-46	12	-42	Temporal Pole
22	3.3	-20	-60	-6	Lingual Gyrus
	3.1	-20	-58	-2	Lingual Gyrus
21	3.5	-40	48	-14	Frontal Pole
21	3.5	-36	-72	-10	Occipital Fusiform Gyrus
20	3.1	-32	8	50	Middle Frontal Gyrus
	2.9	-34	6	56	Middle Frontal Gyrus
20	3.3	54	4	-18	Anterior Superior Temporal Gyrus
19	3.1	-40	-88	-2	Inferior Lateral Occipital Cortex
	3.0	-46	-88	0	Inferior Lateral Occipital Cortex
19	2.9	-32	50	0	Frontal Pole
	2.9	-26	46	4	Frontal Pole
	2.8	-26	46	8	Frontal Pole
	2.7	-34	54	0	Frontal Pole
18	3.3	-50	-32	2	Posterior Superior Temporal Gyrus
17	3.3	-16	26	50	Superior Frontal Gyrus
16	3.1	-24	-40	46	Postcentral Gyrus
	2.8	-24	-44	40	Superior Parietal Lobule
16	3.0	-12	-6	8	Left Thalamus
	2.8	-18	-6	14	Left Cerebral White Matter
16	3.3	-10	-22	66	Precentral Gyrus

		16	3.3	-52	42	-6	Frontal Pole
			3.2	-48	42	-8	Frontal Pole
		16	3.0	-44	2	54	Middle Frontal Gyrus
		15	3.1	-50	2	-22	Anterior Middle Temporal Gyrus
		15	3.0	-10	52	14	Paracingulate Gyrus
		15	3.3	-24	-36	-24	Posterior Temporal Fusiform Cortex
		15	4.0	-60	-58	-16	Inferior Temporal Gyrus
	Neg	17	3.8	-14	38	-20	Frontal Pole
DERS-18	Neg	1019	4.1	10	54	6	Paracingulate Gyrus
			4.1	4	52	8	Paracingulate Gyrus
			3.9	6	54	20	Superior Frontal Gyrus
			3.7	-2	62	12	Frontal Pole
			3.7	-4	64	-6	Frontal Pole
			3.6	2	48	-2	Paracingulate Gyrus
		270	4.2	20	-62	42	Superior Lateral Occipital Cortex
			3.5	18	-62	52	Superior Lateral Occipital Cortex
			3.4	8	-54	20	Precuneus Cortex
			3.3	10	-58	36	Precuneus Cortex
			3.1	2	-46	24	Posterior Cingulate Gyrus
			3.0	-4	-48	20	Posterior Cingulate Gyrus
		218	4.1	-42	22	-18	Frontal Orbital Cortex
			3.7	-30	24	-26	Frontal Orbital Cortex
			3.2	-46	26	-6	Frontal Orbital Cortex
			3.2	-42	28	-8	Frontal Orbital Cortex
			2.8	-38	26	-26	Temporal Pole
		210	3.8	60	-64	10	Inferior Lateral Occipital Cortex
			3.7	62	-56	14	Angular Gyrus
			3.6	58	-58	22	Angular Gyrus
			3.5	62	-58	20	Angular Gyrus
			3.3	62	-56	26	Angular Gyrus
			3.3	68	-46	16	Angular Gyrus
		116	3.5	54	38	-10	Frontal Pole
			3.1	52	38	-16	Frontal Pole
			3.1	56	24	-4	Inferior Frontal Gyrus
			3.0	56	30	0	Inferior Frontal Gyrus
		113	3.4	-46	-48	22	Posterior Supramarginal Gyrus
			3.1	-52	-54	30	Angular Gyrus
		109	3.7	46	26	-18	Frontal Orbital Cortex
			3.2	38	32	-16	Frontal Orbital Cortex
			3.2	46	24	-12	Frontal Orbital Cortex
		98	4.3	28	-100	-10	Occipital Pole
			3.7	34	-96	4	Occipital Pole
			3.0	38	-92	12	Occipital Pole

	3.0	32	-90	0	Inferior Lateral Occipital Cortex
86	3.7	64	2	-18	Anterior Middle Temporal Gyrus
	3.6	54	0	-26	Anterior Middle Temporal Gyrus
	3.1	58	8	-22	Temporal Pole
81	3.6	-26	-102	-4	Occipital Pole
	3.2	-28	-100	-8	Occipital Pole
61	3.7	-30	-16	-16	Left Hippocampus
	3.0	-26	-20	-22	Anterior Parahippocampal Gyrus
	2.8	-24	-22	-16	Posterior Parahippocampal Gyrus
	2.7	-24	-24	-20	Posterior Parahippocampal Gyrus
55	4.8	32	-14	-16	Right Hippocampus
51	3.2	-40	-78	-6	Inferior Lateral Occipital Cortex
	3.2	-34	-90	-6	Inferior Lateral Occipital Cortex
	2.9	-36	-84	-6	Inferior Lateral Occipital Cortex
	2.9	-36	-94	-14	Occipital Pole
	2.9	-36	-94	-10	Occipital Pole
44	3.9	6	54	46	Frontal Pole
	3.4	12	54	42	Frontal Pole
43	3.3	-44	-24	24	Parietal Operculum Cortex
	2.7	-36	-28	24	Parietal Operculum Cortex
42	3.9	20	28	14	Right Cerebral White Matter
41	3.8	-12	-62	40	Precuneus Cortex
	3.0	-10	-56	38	Precuneus Cortex
40	3.4	-52	-12	-10	Posterior Superior Temporal Gyrus
	3.1	-56	-14	-10	Posterior Middle Temporal Gyrus
35	3.0	26	-80	30	Superior Lateral Occipital Cortex
	2.8	24	-84	36	Superior Lateral Occipital Cortex
30	3.4	-16	38	50	Superior Frontal Gyrus
29	3.3	-6	-50	10	Posterior Cingulate Gyrus
28	3.4	46	-86	-4	Inferior Lateral Occipital Cortex
	3.2	42	-90	-10	Inferior Lateral Occipital Cortex
	2.9	44	-86	-16	Inferior Lateral Occipital Cortex
26	3.6	4	36	-6	Anterior Cingulate Gyrus
25	3.8	-36	-74	30	Superior Lateral Occipital Cortex
25	3.1	-6	-42	32	Posterior Cingulate Gyrus
24	3.5	-26	38	14	Middle Frontal Gyrus
24	3.0	-6	60	34	Frontal Pole
24	3.6	-14	-22	34	Posterior Cingulate Gyrus
22	3.2	-38	-26	44	Postcentral Gyrus
22	3.9	-42	12	-30	Temporal Pole
21	3.1	54	-72	-12	Inferior Lateral Occipital Cortex
	3.0	50	-68	-10	Inferior Lateral Occipital Cortex
21	3.6	-34	-38	12	Planum Temporale

		21	3.3	-6	-22	-28	Brain-Stem
			3.2	-10	-24	-20	Brain-Stem
		21	3.8	-40	-14	-22	Posterior Temporal Fusiform Cortex
		19	3.0	4	24	16	Anterior Cingulate Gyrus
			2.8	0	22	12	Anterior Cingulate Gyrus
			2.7	-2	28	12	Anterior Cingulate Gyrus
		18	3.1	-6	-38	58	Postcentral Gyrus
		17	3.2	24	-56	-22	Cerebellar lobule VI
			2.8	14	-54	-26	Cerebellar lobule V
		16	3.2	-2	34	4	Anterior Cingulate Gyrus
		16	3.0	-28	-44	56	Superior Parietal Lobule
		15	3.3	68	-12	10	Central Opercular Cortex
		15	3.3	26	66	12	Frontal Pole
		15	3.3	26	-90	36	Occipital Pole
Daily support provided	Pos	3685	5.2	40	-66	8	Inferior Lateral Occipital Cortex
			4.6	52	-70	4	Inferior Lateral Occipital Cortex
			4.2	50	-46	-2	Middle Temporal Gyrus
			4.1	-50	-78	14	Inferior Lateral Occipital Cortex
			4.1	38	-62	18	Superior Lateral Occipital Cortex
			4.1	20	-82	22	Superior Lateral Occipital Cortex
		520	4.1	2	-40	56	Precuneus Cortex
			4.0	-6	-28	52	Precentral Gyrus
			3.9	-12	-28	34	Posterior Cingulate Gyrus
			3.6	-6	-30	34	Posterior Cingulate Gyrus
			3.3	-12	-36	54	Postcentral Gyrus
			3.3	2	-18	62	Precentral Gyrus
		467	4.1	10	48	-8	Paracingulate Gyrus
			3.8	-16	28	18	Anterior Cingulate Gyrus
			3.5	-4	38	4	Anterior Cingulate Gyrus
			3.3	6	40	8	Anterior Cingulate Gyrus
			3.0	-6	64	16	Frontal Pole
			3.0	-4	58	6	Frontal Pole
		427	3.8	8	-42	10	Posterior Cingulate Gyrus
			3.8	22	-36	-2	Posterior Parahippocampal Gyrus
			3.7	16	-38	-2	Posterior Cingulate Gyrus
			3.5	8	-40	2	Posterior Cingulate Gyrus
			3.4	18	-44	-18	Temporal Occipital Fusiform Cortex
			3.3	12	-42	0	Posterior Cingulate Gyrus
		407	4.1	-14	-22	-24	Anterior Parahippocampal Gyrus
			4.0	-32	-38	4	Left Cerebral White Matter
			4.0	-28	-38	2	Left Cerebral White Matter
			3.9	-26	-4	-16	Anterior Parahippocampal Gyrus
			3.7	-34	-10	-18	Left Cerebral White Matter

	3.5	-32	-20	-10	Left Cerebral White Matter
306	3.9	66	-32	2	Posterior Superior Temporal Gyrus
	3.8	70	-34	16	Posterior Superior Temporal Gyrus
	3.7	42	-32	12	Planum Temporale
	3.4	46	-28	12	Planum Temporale
	3.2	52	-34	14	Planum Temporale
	3.1	56	-30	16	Planum Temporale
261	4.2	-66	-20	6	Posterior Superior Temporal Gyrus
	3.5	-56	-4	0	Planum Polare
	3.4	-58	4	-4	Temporal Pole
	3.2	-48	-2	4	Central Opercular Cortex
	3.2	-40	10	-2	Insular Cortex
	3.1	-54	-20	10	Heschl's Gyrus (includes H1 and H2)
230	4.1	38	-54	62	Superior Parietal Lobule
	4.0	36	-58	62	Superior Lateral Occipital Cortex
	3.5	32	-44	56	Superior Parietal Lobule
	3.5	38	-44	62	Superior Parietal Lobule
	3.2	32	-58	54	Superior Lateral Occipital Cortex
	3.2	26	-44	50	Superior Parietal Lobule
186	4.7	-16	-8	58	Superior Frontal Gyrus
184	3.8	52	-30	38	Anterior Supramarginal Gyrus
	3.8	38	-36	26	Parietal Operculum Cortex
	3.4	46	-20	36	Postcentral Gyrus
	3.3	38	-26	26	Parietal Operculum Cortex
	3.1	34	-30	30	Right Cerebral White Matter
	3.0	52	-16	32	Postcentral Gyrus
148	3.6	60	6	-8	Anterior Superior Temporal Gyrus
	3.5	58	-8	-2	Anterior Superior Temporal Gyrus
	3.2	58	14	-8	Temporal Pole
	3.1	62	-12	4	Planum Temporale
	3.0	68	-16	-2	Posterior Superior Temporal Gyrus
125	3.5	-30	-98	0	Occipital Pole
	3.0	-32	-86	2	Inferior Lateral Occipital Cortex
	3.0	-22	-100	0	Occipital Pole
	2.9	-26	-86	4	Inferior Lateral Occipital Cortex
	2.9	-26	-90	4	Inferior Lateral Occipital Cortex
	2.8	-22	-100	6	Occipital Pole
124	3.3	24	-66	-8	Occipital Fusiform Gyrus
	3.2	24	-62	-14	Temporal Occipital Fusiform Cortex
112	3.9	56	-10	-24	Posterior Middle Temporal Gyrus
	3.7	60	-16	-18	Posterior Middle Temporal Gyrus
	3.3	50	-8	-28	Anterior Inferior Temporal Gyrus
	3.2	52	-6	-24	Anterior Middle Temporal Gyrus

	2.9	66	-16	-22	Posterior Middle Temporal Gyrus
	2.9	62	-12	-14	Posterior Middle Temporal Gyrus
103	3.7	-2	16	-2	Subcallosal Cortex
	3.5	8	20	0	Right Caudate
	3.2	-10	16	-4	Left Accumbens
99	4.3	50	-28	52	Postcentral Gyrus
96	3.6	54	10	26	Precentral Gyrus
	3.6	54	6	24	Precentral Gyrus
	2.6	46	16	24	Inferior Frontal Gyrus
92	3.3	26	14	-10	Frontal Orbital Cortex
	3.2	24	4	-12	Frontal Orbital Cortex
	2.8	12	4	-8	Right Cerebral White Matter
90	3.7	6	54	24	Superior Frontal Gyrus
89	3.7	-20	-74	46	Superior Lateral Occipital Cortex
	3.4	-14	-80	48	Superior Lateral Occipital Cortex
87	3.2	-42	-40	54	Superior Parietal Lobule
	3.1	-38	-34	60	Postcentral Gyrus
	3.1	-38	-40	48	Superior Parietal Lobule
	2.7	-48	-32	58	Postcentral Gyrus
79	3.4	34	-18	-14	Right Hippocampus
	3.4	26	-20	-12	Right Hippocampus
	3.0	22	-14	-18	Right Hippocampus
	2.7	18	-16	-20	Anterior Parahippocampal Gyrus
79	3.9	14	-72	-16	Lingual Gyrus
73	4.2	-56	-16	-14	Posterior Middle Temporal Gyrus
	3.2	-64	-14	-18	Posterior Middle Temporal Gyrus
	3.0	-66	-24	-16	Posterior Middle Temporal Gyrus
	2.9	-64	-20	-18	Posterior Middle Temporal Gyrus
68	4.1	-32	-30	22	Parietal Operculum Cortex
	3.0	-30	-38	22	Posterior Superior Temporal Gyrus
66	3.9	10	-46	30	Posterior Cingulate Gyrus
	3.2	18	-46	34	Precuneus Cortex
64	4.1	-48	2	-34	Temporal Pole
62	3.6	-4	-74	-18	Occipital Fusiform Gyrus
	3.2	-8	-74	-14	Lingual Gyrus
	3.1	-16	-72	-20	Occipital Fusiform Gyrus
59	3.7	-26	6	0	Left Putamen
59	3.5	-42	-54	-8	Inferior Temporal Gyrus
	3.2	-48	-56	-12	Inferior Temporal Gyrus
57	3.1	34	14	42	Middle Frontal Gyrus
	3.1	34	16	48	Middle Frontal Gyrus
57	3.4	-34	-50	60	Superior Parietal Lobule
	2.9	-30	-48	68	Superior Parietal Lobule

	2.7	-30	-44	72	Superior Parietal Lobule
53	3.9	6	-62	52	Precuneus Cortex
53	3.3	36	-10	48	Precentral Gyrus
	3.3	38	-8	42	Precentral Gyrus
52	3.1	16	64	12	Frontal Pole
	3.1	10	64	18	Frontal Pole
	2.9	20	64	20	Frontal Pole
	2.8	24	64	24	Frontal Pole
51	3.5	-16	-50	34	Precuneus Cortex
49	3.4	4	-38	76	Postcentral Gyrus
	3.4	4	-42	70	Postcentral Gyrus
48	4.0	26	26	54	Superior Frontal Gyrus
48	3.5	-40	6	18	Inferior Frontal Gyrus
	3.3	-38	20	18	Inferior Frontal Gyrus
	2.9	-38	26	16	Inferior Frontal Gyrus
47	3.4	42	-52	-22	Temporal Occipital Fusiform Cortex
	2.9	44	-44	-18	Temporal Occipital Fusiform Cortex
46	4.1	22	26	42	Superior Frontal Gyrus
43	3.6	-26	-36	56	Postcentral Gyrus
	2.9	-30	-34	52	Postcentral Gyrus
42	3.3	18	-24	40	Precentral Gyrus
	3.1	12	-28	36	Posterior Cingulate Gyrus
37	3.5	-46	-20	28	Postcentral Gyrus
37	3.6	-14	-50	8	Precuneus Cortex
35	3.1	20	-24	56	Precentral Gyrus
	3.0	20	-24	70	Precentral Gyrus
	3.0	22	-22	60	Precentral Gyrus
35	3.8	22	52	10	Frontal Pole
32	3.4	-8	-38	10	Posterior Cingulate Gyrus
	3.3	-14	-36	6	Left Cerebral White Matter
31	3.5	2	-30	-16	Brain-Stem
31	3.3	56	-22	6	Planum Temporale
	3.0	56	-16	6	Planum Temporale
31	3.4	-28	38	-6	Frontal Orbital Cortex
28	3.5	-16	4	40	Paracingulate Gyrus
27	3.2	-16	-54	60	Superior Parietal Lobule
27	3.1	56	-18	20	Central Opercular Cortex
	3.0	52	-14	22	Central Opercular Cortex
27	3.0	-12	-32	74	Postcentral Gyrus
	3.0	-10	-28	72	Precentral Gyrus
25	3.2	-18	-14	-22	Anterior Parahippocampal Gyrus
25	3.0	-42	-16	4	Heschl's Gyrus (includes H1 and H2)
	3.0	-34	-12	2	Insular Cortex

25	3.1	18	-4	72	Superior Frontal Gyrus
	2.8	14	-8	68	Superior Frontal Gyrus
24	3.4	20	10	16	Right Cerebral White Matter
24	3.6	54	-56	-22	Inferior Temporal Gyrus
24	3.0	-36	28	-18	Frontal Orbital Cortex
	3.0	-38	36	-14	Frontal Orbital Cortex
23	3.3	24	-50	58	Superior Parietal Lobule
	3.2	16	-50	58	Superior Parietal Lobule
22	3.0	-14	-60	8	Precuneus Cortex
	2.8	-14	-64	0	Lingual Gyrus
21	3.2	4	-10	26	Anterior Cingulate Gyrus
	2.9	-4	-8	28	Anterior Cingulate Gyrus
21	3.1	10	-18	18	Right Thalamus
	2.8	6	-6	16	Right Thalamus
	2.8	12	-8	20	Right Lateral Ventricle
20	3.0	-26	-26	6	Left Cerebral White Matter
	2.6	-22	-28	18	Left Cerebral White Matter
19	2.9	-24	-8	4	Left Putamen
	2.8	-20	-6	-2	Left Pallidum
19	3.3	-12	-12	-6	Left Cerebral White Matter
	2.8	-6	-10	-4	Left Thalamus
19	3.3	24	-36	-32	Brain-Stem
19	3.3	-50	2	24	Precentral Gyrus
18	3.2	-8	-12	38	Anterior Cingulate Gyrus
18	3.2	54	-16	58	Postcentral Gyrus
	3.0	56	-16	52	Postcentral Gyrus
17	3.1	42	44	10	Frontal Pole
17	3.4	-40	-6	28	Precentral Gyrus
17	3.5	6	20	18	Anterior Cingulate Gyrus
16	3.3	-38	-38	32	Left Cerebral White Matter
16	3.1	30	-6	-4	Right Putamen
15	3.2	10	18	46	Paracingulate Gyrus
15	3.5	26	-20	-30	Anterior Parahippocampal Gyrus
15	3.2	38	0	30	Precentral Gyrus

Supplementary Table 10. Comparisons of relationships between BOLD activity and daily support provided to the partner

ROI	$S_{NEG}P_{POS}$-$S_{NEG}P_{XX}$			$S_{XX}P_{POS}$-$S_{XX}P_{XX}$			$S_{XX}P_{NEG}$-$S_{XX}P_{XX}$			$S_{NEG}P_{POS}$-$S_{NEG}P_{XX}$ vs. $S_{XX}P_{POS}$-$S_{XX}P_{XX}$		$S_{NEG}P_{POS}$-$S_{NEG}P_{XX}$ vs. $S_{XX}P_{NEG}$-$S_{XX}P_{XX}$	
	β	*SE*	*p*	β	*SE*	*p*	β	*SE*	*p*	*t*	*p*	*t*	*p*
DMPFC (4, 56, 24)	0.23	0.09	0.008	-0.07	0.09	0.417	-0.02	0.09	0.795	2.47	0.013	2.08	0.037

β: *Standardized coefficient.*

DMPFC: dorsomedial prefrontal cortex.

Supplementary Note 1. Analyses involving Empathy Assessment Index

We initially used the Empathy Assessment Index (EAI) due to its conceptual alignment with our hypotheses and then changed to the field-standard measures (i.e., the IRI and DERS) to strengthen the methodological rigor and interpretability of the study. The EAI includes 22 items on a 6-point scale. It is built on five components of interpersonal empathy: (a) Affective Response, (b) Affective Mentalizing, (c) Self-Other Awareness, (d) Perspective-Taking, and (e) Emotion Regulation[1]. Given the self-regulatory demands inherent to the simultaneous feedback situations, the current study primarily used the emotion regulation subscale to examine brain regions involved in self-regulation during empathy. This subscale measures an individual's internal ability to change or control one's own emotional experience (e.g., “When I am upset or unhappy, I get over it quickly”).

Analyses using the EAI yielded a similar pattern of results as reported in the manuscript. Specifically, greater scores on the emotion regulation subscale of the Empathy Assessment Index were positively related to affective ratings in the $S_{NEG}P_{POS}$-$S_{NEG}P_{XX}$ contrast ($\beta = 0.23$, $p = 0.008$, $N = 131$), higher emotional similarity with the partner during the $S_{NEG}P_{POS}$ condition (($\beta = 0.19$, $p = 0.033$, $N = 128$), and increased activity in the right dorsomedial prefrontal cortex in the $S_{NEG}P_{POS}$-$S_{NEG}P_{XX}$ contrast ($\beta = 0.19$, $p = 0.027$, $N = 131$).

Supplementary Note 2. Dyadic Analyses: inter-partner representational similarity and its associations with relationship satisfaction

Backgrounds

Exploring empathy within a dyadic framework offers a new means of understanding the relational correlates of neural responses during challenging social situations. In this framework, relationship outcomes are jointly determined by both partners' neural responses. For example, an individual's relationship satisfaction may be linked to their own spontaneous neural empathic responses (actor effect), as well as those of their partner (partner effect)[2,3]. Here we aim to test the associations between inter-partner similarity in neural empathic responses to challenging social situations and

relationship satisfaction. Inter-partner similarity in empathy has been found to be associated with more positive relational outcomes[4], supporting the similarity hypothesis[5]. However, it remains unclear whether partner similarity in empathic responses is uniformly advantageous. The opposite outcome would support the complementarity hypothesis, which argues that partners are more satisfied when they differ in certain traits, as these differences allow them to complement each other[6]. For instance, if both partners show limited evidence of empathic responding, their similarity might be detrimental rather than beneficial. Alternatively, the totality hypothesis proposes that a couple's overall level of specific characteristics, rather than their similarities or differences, have the greatest influence on relationship quality[7]. Overall, further research is needed to disentangle these effects by examining the spontaneous neural responses of both partners in challenging empathy situations.

Methods

Revised Dyadic Adjustment Scale (RDAS)

The RDAS includes 14 items, each of which asks the approximate extent of agreement or disagreement between one and one's partner on a 6- or 5-point scale (item 11). The RDAS includes seven dimensions of couple relationships within three overarching categories: consensus in decision making, values and affection, satisfaction in the relationship with respect to stability and conflict regulation, and cohesion as seen through activities and discussion[8]. Sum scores on the RDAS range from 0 to 69 with higher scores indicating greater relationship satisfaction and lower scores indicating greater relationship distress. The overall romantic relationship satisfaction of each couple is calculated as the average of self-reported RDAS score of both partners. Two participants didn't complete this questionnaire (N = 129).

Data analyses

To investigate the associations between romantic relationship satisfaction, spontaneous brain activity, and inter-partner representational similarity during the challenging empathy conditions, we conducted dyadic analyses using data from a subset of 60 couples (N = 120) where both partners completed the scanning session. First, Actor-Partner Interdependence Models (APIMs)[9] were used to investigate the

interdependent and simultaneous effects of individual's and partner's spontaneous empathic responses during $S_{NEG}P_{POS}$-$S_{NEG}P_{XX}$ contrast on relationship satisfaction.

Next, we ran a representational similarity analysis (RSA) between partners in the $S_{NEG}P_{POS}$ condition, using methods outlined in Lee et al.[10]. Specifically, we estimated single-trial activation patterns using least squares single methods[11], where one model was constructed for each $S_{NEG}P_{POS}$ trial. Each single-level general linear model included one regressor for the feedback stimulus presentation phase of the given trial, one regressor for the feedback stimuli presentation phrase of the remaining $S_{NEG}P_{POS}$ trials, eight regressors for the feedback stimuli presentation phase of all trials of other conditions (e.g., $S_{NEG}P_{NEG}$ or $S_{POS}P_{POS}$ condition), and one regressor for the affective rating phase of all trials. The voxel-wise pattern of activity, represented by z-values (i.e., z-transformed parameter estimates), for each $S_{NEG}P_{POS}$ trial was extracted within each ROI region for each participant. The pairwise Pearson correlation coefficients were calculated between every possible trial pairing between partners, producing an asymmetric 24*24 similarity matrix for each ROI in each dyad (e.g., trial 1 of partner 1 with trial 1 of partner 2, trial 1 of partner 1 with trial 2 of partner 2, and so on). The Fisher's r-to-z transformed Pearson correlation coefficients were averaged to represent the inter-partner representational similarity values (Supplementary Figure 3A–B).

Additionally, the mean activity value for each $S_{NEG}P_{POS}$ trial was also extracted and averaged across all $S_{NEG}P_{POS}$ trials as the activity strength for each ROI per participant. Each couple's overall activity strength was computed by averaging the activity strength of both partners in each dyad. Finally, a regression model was constructed for each ROI at the couple level, where each couple's overall activity strength, inter-partner representational similarity, and their interaction were included to predict the overall romantic relationship satisfaction of dyads (i.e., Overall romantic relationship satisfaction = b0 + b1 Overall activity strength + b2 Inter-partner representational similarity value + b3 Overall activity strength* Inter-partner representational similarity value + e). The totality hypothesis would be supported if b1 was significantly different from zero. The similarity hypothesis would be supported if b2 was significantly positive, while the complementarity hypothesis would be supported if b2 was significantly negative. A significant b3 would suggest a significant interaction

effect between partner similarity and overall activity strength on overall romantic relationship satisfaction.

Results

Building on our prior ROI findings linking DMPFC activity to emotion regulation tendencies and empathic behaviors in daily life, we conducted a set of dyadic analyses. First, results of Actor-partner Interdependence Modelling revealed no significant associations between relationship satisfaction and either their own or partner's BOLD response in the $S_{NEG}P_{POS}$-$S_{NEG}P_{XX}$ contrast.

Next, we investigated whether inter-partner representational similarity, couple-level overall (mean) activity in these regions, or their interaction in the challenging empathy condition (i.e., $S_{NEG}P_{POS}$) was associated with the couple's overall level of romantic relationship satisfaction. No significant main effects of either inter-partner representational similarity or couple-level overall activity strength on overall relationship satisfaction were observed. However, a significant interaction emerged between couple-level overall activity strength and inter-partner representational similarity in the DMPFC in the $S_{NEG}P_{POS}$ condition (β = 0.32, SE = 0.15, 95% CI: [0.03, 0.60], t = 2.15, p = 0.036, partial η^2 = 0.08). A Johnson-Neyman stepdown analysis was conducted to determine the range of couple-level overall DMPFC activity within which inter-partner representational similarity was significantly associated with couple's overall relationship satisfaction. The analysis revealed that as couple-level overall DMPFC activity increased, the relationship between inter-partner representation similarity and overall relationship satisfaction changed from significantly negative (at the critical point of overall DMPFC activity = -2.36 SD; β = -0.68, SE = 0.34, 95% CI: [-1.36, 0.00], t = -2.00, p = 0.050) to significantly positive (at the critical point of overall DMPFC activity = +2.11 SD; β = 0.73, SE = 0.36, 95% CI: [0.00, 1.46], t = 2.00, p = 0.050; Supplementary Figure 3C). In other words, when couples' overall (mean) DMPFC activity was low, those with higher inter-partner representational similarity—indicating that both partners showed lower DMPFC activity—reported lower relationship satisfaction. In contrast, when couples' overall (mean) DMPFC activity was high, those with higher inter-partner

representational similarity—indicating that both partners showed higher DMPFC activity—reported greater relationship satisfaction.

Interpretations

As an exploratory analysis, we leveraged the dyadic nature of our study to investigate inter-partner representational similarity in neural responses. We observed that in the $S_{NEG}P_{POS}$ condition, the association between inter-partner representational similarity in the DMPFC and relationship satisfaction was moderated by overall DMPFC activity strength. Although similarity in empathic accuracy between romantic partners has been associated with more positive relational outcomes[4], our findings may suggest that similarity in how partners' respond neurally to challenging empathy contexts is not always related to better relationship outcomes[12–14], but rather depends on the overall level of neural activity. Specifically, high pattern similarity paired with low overall DMPFC activity may reflect a mutual disengagement or reduced mentalizing effort or empathic motivations, potentially indicating that both partners are less sensitive or attuned to each other's mental states. This may be particularly associated with lower relationships satisfaction. In contrast, high pattern similarity coupled with high overall activation may reflect active, reciprocal engagement in mentalizing or empathic motivations—suggesting not only alignment, but effortful, socially meaningful processing, which could support relationship quality. This aligns with prior evidence indicating that individuals with lower self-control often prefer partners with higher self-control, implying that complementary rather than similar self-regulatory capacities may be beneficial for relationship outcomes, when one partner has lower self-control[15]. While these preliminary findings require further study to replicate and further probe the mechanisms underlying this effect, we believe they highlight the importance of considering relational outcomes as a joint focus of both partners' neural responses.

References

1. Lietz, C. A. *et al.* The Empathy Assessment Index (EAI): A Confirmatory Factor Analysis of a Multidimensional Model of Empathy. *J. Soc. Soc. Work Res.* **2**, 104–124 (2011). https://doi.org/10.5243/jsswr.2011.6.
2. Kenny, D. A., Kashy, D. A. & Cook, W. L. *Dyadic Data Analysis*. (Guilford Publications, 2020).
3. Kimmes, J. G., Edwards, A. B., Wetchler, J. L. & Bercik, J. Self and Other Ratings of Dyadic Empathy as Predictors of Relationship Satisfaction. *Am. J. Fam. Ther.* **42**, 426–437 (2014). https://doi.org/10.1080/01926187.2014.925374.
4. Berlamont, L. *et al.* Couple similarity in empathic accuracy and relationship well-being. *J. Soc. Pers. Relatsh.* **41**, 1301–1323 (2024). https://doi.org/10.1177/02654075231206412.
5. Byrne, D. & Griffitt, W. Interpersonal attraction. *Annu. Rev. Psychol.* **24**, 317–336 (1973). https://doi.org/10.1146/annurev.ps.24.020173.001533.
6. Winch, R. F. The Theory of Complementary Needs in Mate-Selection: Final Results on the Test of the General Hypothesis. *Am. Sociol. Rev.* **20**, 552–555 (1955). https://doi.org/10.2307/2092563.
7. Vohs, K. D., Finkenauer, C. & Baumeister, R. F. The Sum of Friends' and Lovers' Self-Control Scores Predicts Relationship Quality. *Soc. Psychol. Personal. Sci.* **2**, 138–145 (2011). https://doi.org/10.1177/1948550610385710.
8. Busby, D. M., Christensen, C., Crane, D. R. & Larson, J. H. A Revision of the Dyadic Adjustment Scale for Use with Distressed and Nondistressed Couples: Construct Hierarchy and Multidimensional Scales. *J. Marital Fam. Ther.* **21**, 289–308 (1995). https://doi.org/10.1111/j.1752-0606.1995.tb00163.x.
9. Sened, H. *et al.* Empathic accuracy and relationship satisfaction: A meta-analytic review. *J. Fam. Psychol.* **31**, 742–752 (2017). https://doi.org/10.1037/fam0000320.

10. Lee, T.-H., Qu, Y. & Telzer, E. H. Dyadic Neural Similarity During Stress in Mother–Child Dyads. *J. Res. Adolesc.* **28**, 121–133 (2018). https://doi.org/10.1111/jora.12334.

11. Mumford, J. A., Davis, T. & Poldrack, R. A. The impact of study design on pattern estimation for single-trial multivariate pattern analysis. *NeuroImage* **103**, 130–138 (2014). https://doi.org/10.1016/j.neuroimage.2014.09.026.

12. Zhou, Y., Wang, K., Chen, S., Zhang, J. & Zhou, M. The Actor, Partner, Similarity Effects of Personality, and Interactions with Gender and Relationship Duration among Chinese Emerging Adults. *Front. Psychol.* **8**, (2017). https://doi.org/10.3389/fpsyg.2017.01698.

13. Kardum, I., Hudek-Knezevic, J., Mehic, N. & Pilek, M. The effects of similarity in the dark triad traits on the relationship quality in dating couples. *Personal. Individ. Differ.* **131**, 38–44 (2018). https://doi.org/10.1016/j.paid.2018.04.020.

14. Dryer, D. C. & Horowitz, L. M. When do opposites attract? Interpersonal complementarity versus similarity. *J. Pers. Soc. Psychol.* **72**, 592–603 (1997). https://doi.org/10.1037/0022-3514.72.3.592.

15. Shea, C. T., Davisson, E. K. & Fitzsimons, G. M. Riding Other People's Coattails: Individuals With Low Self-Control Value Self-Control in Other People. *Psychol. Sci.* **24**, 1031–1036 (2013). https://doi.org/10.1177/0956797612464890.
