# Supplementary material for: Neural responses to conflicting self- and partner-directed feedback correlate with trait emotion regulation and daily support
Source: Commun Psychol. 2026 Jul 2;4:147. doi: 10.1038/s44271-026-00493-w (PMC13690568; doi:10.1038/s44271-026-00493-w)
Supplement: Supplementary file 3 [file 44271_2026_493_MOESM3_ESM.pdf]

nature portfolio

Corresponding author(s): Jessica R. Andrews-Hanna

Last updated by author(s): Jun 1, 2026

Reporting Summary

Nature Portfolio wishes to improve the reproducibility of the work that we publish. This form provides structure for consistency and transparency in reporting. For further information on Nature Portfolio policies, see our Editorial Policies and the Editorial Policy Checklist.

Statistics

For all statistical analyses, confirm that the following items are present in the figure legend, table legend, main text, or Methods section.

n/a	Confirmed	
☐	☒	The exact sample size (*n*) for each experimental group/condition, given as a discrete number and unit of measurement
☐	☒	A statement on whether measurements were taken from distinct samples or whether the same sample was measured repeatedly
☐	☒	The statistical test(s) used AND whether they are one- or two-sided<br>*Only common tests should be described solely by name; describe more complex techniques in the Methods section.*
☐	☒	A description of all covariates tested
☐	☒	A description of any assumptions or corrections, such as tests of normality and adjustment for multiple comparisons
☐	☒	A full description of the statistical parameters including central tendency (e.g. means) or other basic estimates (e.g. regression coefficient) AND variation (e.g. standard deviation) or associated estimates of uncertainty (e.g. confidence intervals)
☐	☒	For null hypothesis testing, the test statistic (e.g. *F*, *t*, *r*) with confidence intervals, effect sizes, degrees of freedom and *P* value noted<br>*Give P values as exact values whenever suitable.*
☒	☐	For Bayesian analysis, information on the choice of priors and Markov chain Monte Carlo settings
☐	☒	For hierarchical and complex designs, identification of the appropriate level for tests and full reporting of outcomes
☐	☒	Estimates of effect sizes (e.g. Cohen's *d*, Pearson's *r*), indicating how they were calculated

Our web collection on statistics for biologists contains articles on many of the points above.

Software and code

Policy information about availability of computer code

Data collection	PsychoPy
Data analysis	fmriprep_v23.0.1<br>FSL 6.0.7.9<br>R (R Core Team, version 4.4.0)<br>The analysis was conducted using a combination of publicly available toolboxes and custom code. The custom analysis code supporting the core findings of this study (utilizing R packages such as lmer) has been deposited in the Open Science Framework and is publicly accessible at https://doi.org/10.17605/OSF.IO/63VNC.

For manuscripts utilizing custom algorithms or software that are central to the research but not yet described in published literature, software must be made available to editors and reviewers. We strongly encourage code deposition in a community repository (e.g. GitHub). See the Nature Portfolio guidelines for submitting code & software for further information.

Data

Policy information about availability of data

All manuscripts must include a data availability statement. This statement should provide the following information, where applicable:

- Accession codes, unique identifiers, or web links for publicly available datasets
- A description of any restrictions on data availability
- For clinical datasets or third party data, please ensure that the statement adheres to our policy

The minimal dataset necessary to interpret and replicate the findings reported in this article has been publicly deposited. The intermediate behavioral measures, ROI-extracted values, and whole-brain activity maps generated during this study have been deposited in the Open Science Framework and are publicly accessible at https://doi.org/10.17605/OSF.IO/63VNC.

Research involving human participants, their data, or biological material

Policy information about studies with human participants or human data. See also policy information about sex, gender (identity/presentation), and sexual orientation and race, ethnicity and racism.

Reporting on sex and gender	In our study, we asked participants to indicate their gender in a survey. The data sample comprises 71 women, 51 men, 1 trans man, and 8 individuals who identified as non-binary, other, or preferred not to disclose their gender. We reported the detailed information in Supplementary Table S1. We didn't collect the sex information, and we did not perform gender-based analyses as they were not relevant to the current objectives of the study.
Reporting on race, ethnicity, or other socially relevant groupings	The socially relevant categorization variables used in our study were race, ethnicity, sexual orientation, and education. Each variable was used independently and not as a proxy for other constructs.<br><br>Race: Participants self-identified their race by selecting from predefined categories, including White, American Indian or Alaska Native, Native Hawaiian or Other Pacific Islander, Asian, Black or African American, Other, and Prefer not to disclose.<br><br>Ethnicity: Participants indicated their ethnicity based on self-identification, specifically identifying whether they are Hispanic or Latino.<br><br>Sexual Orientation: Participants self-identified their sexual orientation by selecting from predefined categories, including Bisexual, Gay/Lesbian, Heterosexual, Other, and Prefer not to disclose.<br><br>Education: Participants reported their highest level of education completed by selecting from predefined categories, including High school degree or equivalent, Bachelor's degree, Master's degree, Doctorate, Other, and Prefer not to disclose.
Population characteristics	The final sample had a mean age of 31.8 years (SD = 8.4, range = 19.1–56.1). The mean relationship duration was 8.3 years (SD = 6.3, range = 1.7–30.0), and the mean cohabiting duration was 6.5 years (SD = 6.2, range = 0.5–29.5). Participants were also asked to indicate their marital status, including whether they are married, previously divorced, and if they have children.
Recruitment	Participants were recruited from the Tucson community using flyers and advertisements on social media.
Ethics oversight	The study was approved by the Institutional Review Board of the University of Arizona and all participants provided written informed consent prior to participation.

Note that full information on the approval of the study protocol must also be provided in the manuscript.

Field-specific reporting

Please select the one below that is the best fit for your research. If you are not sure, read the appropriate sections before making your selection.

☐ Life sciences ☒ Behavioural & social sciences ☐ Ecological, evolutionary & environmental sciences

For a reference copy of the document with all sections, see nature.com/documents/nr-reporting-summary-flat.pdf

Behavioural & social sciences study design

All studies must disclose on these points even when the disclosure is negative.

Study description	The current study employed functional magnetic resonance imaging (fMRI) data obtained using a newly developed paradigm. Additionally, it utilized self-reported empathy trait and romantic relationship satisfaction assessed using questionnaires,as well as partner-rated daily support provision data collected through daily diaries. All data are quantitative.
Research sample	The final neuroimaging sample contained 131 subjects comprising 71 women, 51 men, 1 trans man, and 8 individuals who identified as non-binary, other, or preferred not to disclose their gender. The mean age of the final sample was 31.8 years (SD = 8.4, range = 19.1–56.1).

Sampling strategy	Convenience/availability sampling (participants signed up to participate, and were included if they fulfilled the inclusion criteria).This study is part of a larger ongoing study involving the collection of neural, physiological, and behavioral data among romantic couples. Regarding the affective rating data, with a sample size of 131, our study achieves a statistical power of 99% to detect an effect size of Cohen's d = 0.34. This level of power indicates a high likelihood of identifying true effects in distinguishing affective ratings of our new contrasts compared to contrasts used in previous studies. For fMRI data, our current sample size is relatively large, which enhances the reliability and robustness of our findings. Larger sample sizes in neuroimaging studies help reduce the impact of noise and increase the generalizability of the results.
Data collection	Behavioral data were collected via laptops in our lab, and fMRI data were collected using a Siemens 3T Trio scanner. No one else was present in the testing room besides the participant. The experimenter was not blind to the study hypothesis during data collection.
Timing	7 September 2021 - 25 May 2023
Data exclusions	Fifteen participants were excluded from the subsequent analysis due to either the absence of task or structural neuroimaging scans (n=10), or compromised neuroimaging data resulting from severe coverage loss or asynchrony between the experimental procedure and brain scanning (n=5).
Non-participation	Seven participants were unable to complete the fMRI scanning due to either physical unsuitability for scanning or discomfort during the procedure, resulting in a response rate of 95% for the fMRI component. Additionally, 3 participants completed only part of the fMRI scanning (such as just the structural or resting-state scans).
Randomization	Participants were not allocated into experimental groups.

Reporting for specific materials, systems and methods

We require information from authors about some types of materials, experimental systems and methods used in many studies. Here, indicate whether each material, system or method listed is relevant to your study. If you are not sure if a list item applies to your research, read the appropriate section before selecting a response.

Materials & experimental systems

n/a	Involved in the study
☒	☐ Antibodies
☒	☐ Eukaryotic cell lines
☒	☐ Palaeontology and archaeology
☒	☐ Animals and other organisms
☒	☐ Clinical data
☒	☐ Dual use research of concern
☒	☐ Plants

Methods

n/a	Involved in the study
☒	☐ ChIP-seq
☒	☐ Flow cytometry
☐	☒ MRI-based neuroimaging

Plants

Seed stocks	*Report on the source of all seed stocks or other plant material used. If applicable, state the seed stock centre and catalogue number. If plant specimens were collected from the field, describe the collection location, date and sampling procedures.*
Novel plant genotypes	*Describe the methods by which all novel plant genotypes were produced. This includes those generated by transgenic approaches, gene editing, chemical/radiation-based mutagenesis and hybridization. For transgenic lines, describe the transformation method, the number of independent lines analyzed and the generation upon which experiments were performed. For gene-edited lines, describe the editor used, the endogenous sequence targeted for editing, the targeting guide RNA sequence (if applicable) and how the editor was applied.*
Authentication	*Describe any authentication procedures for each seed stock used or novel genotype generated. Describe any experiments used to assess the effect of a mutation and, where applicable, how potential secondary effects (e.g. second site T-DNA insertions, mosiacism, off-target gene editing) were examined.*

Magnetic resonance imaging

Experimental design

Design type	Task, event-related
Design specifications	Participants completed 6 runs of the CLOSE task, and each run consisted of 36 trials with 4 trials per condition. The trial order was counterbalanced with a 1.3-7.8 s jittered fixation inter-trial interval (ITI), which was determined using the fMRI trial timing optimization program (optseq2). In total, the duration of each run was approximately 5 minutes and 35 seconds.

Behavioral performance measures

After half of the trials within each condition, participants were asked to rate their affective state on a scale ranging from very negative to very positive.

Acquisition

Imaging type(s)

Functional MRI

Field strength

3T

Sequence & imaging parameters

Functional MR imaging was conducted with a 3-Tesla Siemens MRI scanner. Forty-two interleaved axial slices were acquired using a gradient-echo echo-planar imaging sequence with repetition time = 1300 ms, echo time = 30 ms, flip angle = 68°, field of view = 252 mm x 252 mm, voxel size = 3 x 3 x 3 mm3, matrix size = 84 x 84, and acceleration factor = 2. One hundred seventy-six high-resolution slices were obtained using a T1-weighted sagittal 3-D magnetization prepared rapid gradient echo sequence: repetition time = 2300 ms, echo time = 2.95 ms, inversion time = 900 ms, flip angle = 9°, field of view = 270 mm x 270 mm, voxel size = 1.1 x 1.1 x 1 mm3, and matrix size = 256 x 256. Forty-two axial field map slices were acquired using a gradient-echo sequence: repetition time = 467 ms, echo time1 = 4.92 ms, echo time2 = 7.38 ms, flip angle = 60°, field of view = 224 mm x 224 mm, voxel size = 3 3 3 mm3, and matrix size = 74 x 74.

Area of acquisition

Whole brain

Diffusion MRI ☐ Used ☒ Not used

Preprocessing

Preprocessing software

The functional data was preprocessed using the robust open-source preprocessing pipeline, fMRIPrep (v23.0.1). Preprocessing steps included field map-based distortion correction (FUGUE), motion correction (MCFLIRT), slice timing correction (3dTShift), co-registration to T1-weighted structural images (bbregister), normalization to the MNI152NLin6Asym template with a 2-mm isotropic resolution through nonlinear registration (antsRegistration), spatial smoothing using an isotropic Gaussian kernel with a full-width at half-maximum (FWHM) of 6 mm, and automatic removal of motion artifacts using independent component analysis (ICA-AROMA).

Normalization

Data were normalized to the MNI152NLin6Asym template with a 2-mm isotropic resolution through nonlinear registration (antsRegistration).

Normalization template

The MNI152NLin6Asym template

Noise and artifact removal

Head motion artifacts were removed using independent component analysis (ICA-AROMA).

Volume censoring

None

Statistical modeling & inference

Model type and settings

The cleaned data from fMRIPrep was passed to FSL's fMRI Expert Analysis Tool (FEAT, version 6.00, FMRIB's Software Library, www.fmrib.ox.ac.uk/fsl) for task-related general linear model analyses. The BOLD signal was modeled by convolving the onset and duration of 3.9-s feedback presentation phases for each of the 9 experimental conditions with a double-gamma hemodynamic response function. A separate regressor was also modeled to account for the affect ratings. Then, a second-level fixed-effects analysis was used to compute the average of the parameter estimates across the runs within each subject, and a third-level analysis with mixed effects (FLAME1) was used to test generalization to the population from which our sample was drawn.

Effect(s) tested

To measure spontaneous responses to partner-directed feedback under low demand for regulating responses to self-directed feedback, we compared partner-only conditions—where the self (S) received no feedback and the partner (P) received positive or negative feedback (SXXPPOS or SXXPNEG)—to a neutral control condition matched on visual properties but lacking self-directed or partner-directed feedback (SXXPXX) (see Methods for cover story behind feedback-absent conditions). These partner-alone feedback contrasts (SXXPPOS-SXXPXX and SXXPNEG-SXXPXX) are similar to conventional empathy contrasts used in previous research. Additionally, to evaluate spontaneous responses to partner-directed feedback under higher demand for regulating responses to self-directed feedback, we contrasted simultaneous incongruent conditions—where opposing positive and negative feedback was directed toward the self and partner (SPOSPNEG or SNEGPPOS)—against self-only conditions involving positive or negative self-directed feedback (SPOSPXX or SNEGPXX). We refer to these contrasts (SPOSPNEG-SPOSPXX and SNEGPPOS-SNEGPXX) as incongruent feedback contrasts in our study.

Specify type of analysis: ☐ Whole brain ☐ ROI-based ☒ Both

Anatomical location(s)

Spherical ROIs (radius = 6mm) were defined in the AI, ACC, TPJ, and DMPFC, which are linked to empathy, the MFG, associated with emotion regulation, and right SMG, identified as crucial for overcoming emotional egocentricity bias. The ROIs within the brain were defined based on the peak MNI coordinates obtained using the terms "empathy" and "emotion regulation" from Neurosynth (https://neurosynth.org), as well as the coordinates (68, -38, 36) reported by Silani et al. for overcoming emotional egocentricity bias.

Statistic type for inference (See Eklund et al. 2016)	Voxel-level
Correction	We used a cluster level threshold of pGRF < .05.

Models & analysis

n/a	Involved in the study
☒	☐ Functional and/or effective connectivity
☒	☐ Graph analysis
☐	☒ Multivariate modeling or predictive analysis

Multivariate modeling and predictive analysis	We also ran an exploratory representational similarity analysis (RSA) between partners in the SNEGPPOS condition. Furthermore, to examine the relationship between the inter-partner representational similarity and overall romantic relationship satisfaction, a regression model was constructed for each ROI, where the overall activity strength, inter-partner representational similarity, and their interaction were included to predict the overall romantic relationship satisfaction of dyads (i.e., Overall romantic relationship satisfaction = b0 + b1 Overall activity strength + b2 Inter-partner representational similarity value + b3 Overall activity strength* Inter-partner representational similarity value + e).
